# Diel rhythmicity of activity and corticosterone metabolites in Arctic barnacle geese during breeding

**DOI:** 10.1093/beheco/araf071

**Published:** 2025-06-13

**Authors:** Margje E de Jong, Annabel J Slettenhaar, Rienk W Fokkema, Marion Leh, Mo A Verhoeven, Larry R Griffin, Eva Millesi, Børge Moe, Elisabeth Barnreiter, Maarten J J E Loonen, Isabella B R Scheiber

**Affiliations:** Arctic Centre, University of Groningen, Aweg 30, 9718 CW Groningen, The Netherlands; Dept. of Behavioral and Cognitive Biology, University of Vienna, Djerassiplatz 1, 1030 Vienna, Austria; Arctic Centre, University of Groningen, Aweg 30, 9718 CW Groningen, The Netherlands; Faculty of Biosciences and Aquaculture, Nord University, Skolegata 22, 7713 Steinkjer, Norway; Conservation Ecology Group, Groningen Institute for Evolutionary Life Sciences (GELIFES), University of Groningen, Nijenborgh 7, 9747 AG, Groningen, The Netherlands; BirdEyes, Centre for Global Ecological Change at the Faculties of Science & Engineering and Campus Fryslân, University of Groningen, Wirdumerdijk 34, 8911 CE Leeuwarden, The Netherlands; Dept. of Behavioral and Cognitive Biology, University of Vienna, Djerassiplatz 1, 1030 Vienna, Austria; NIOZ Royal Netherlands Institute for Sea Research, Landsdiep 4, 1797 SZ 't Horntje (Texel), The Netherlands; Department of Animal Ecology, Netherlands Institute of Ecology (NIOO-KNAW), Droevendaalsesteeg 10, 6708 PB Wageningen, the Netherlands; ECO-LG Ltd, Crooks House, Mabie, DG2 8EY, United Kingdom; Dept. of Behavioral and Cognitive Biology, University of Vienna, Djerassiplatz 1, 1030 Vienna, Austria; Norwegian Institute for Nature Research (NINA), Høgskoleringen 9, 7034 Trondheim, Norway; Dept. of Behavioral and Cognitive Biology, University of Vienna, Djerassiplatz 1, 1030 Vienna, Austria; Arctic Centre, University of Groningen, Aweg 30, 9718 CW Groningen, The Netherlands; Dept. of Behavioral and Cognitive Biology, University of Vienna, Djerassiplatz 1, 1030 Vienna, Austria

**Keywords:** camera trap, circadian rhythmicity, GPS transmitter, hormones, fecal glucocorticoid metabolites, polar

## Abstract

Birds that migrate from temperate areas to the Arctic to breed lose their strongest *Zeitgeber* of circadian organization when they cross the Arctic circle in spring – the 24h light-dark cycle. Under continuous daylight, diverse behavioral and physiological patterns have been detected in both free-ranging and laboratory animals. To better understand the evolution of plasticity in circadian clocks, it is essential to study behavioral and physiological rhythmicity in the context of a species’ ecology. Employing a multifaceted approach, which included wildlife cameras, accelerometers, and noninvasive sampling of hormone metabolites, we investigated activity patterns and corticosterone rhythmicity in a migratory herbivore, the barnacle goose (*Branta leucopsis*), during its Arctic breeding season on Svalbard. We found that females showed a combination of both ultradian and diel rhythmicity in nest recesses and sleep during incubation. In both parents, these rhythms in activity continued also during the gosling rearing phase. During molt, many geese aligned activity with the prevailing tidal rhythm. Barnacle geese showed weak diel rhythmicity in excreted corticosterone metabolites (CORTm). This suggests that while Arctic geese may adopt an alternative *Zeitgeber* during the Arctic summer to maintain a diel rhythm, ultradian rhythmicity remains essential, allowing the geese to flexibly adjust their rhythms to environmental conditions.

## Teaser

Despite the continuous daylight of the Arctic summer, barnacle geese manage to keep rhythmic behavior. Arctic summers may pose a challenge to animals reliant on a day-night cycle. We found that migratory barnacle geese show short cycles (ultradian rhythms), daily patterns (diel rhythms) and even rhythms, which coincide with the tides, in their activity behavior. Contrastingly, stress hormones (corticosterone) showed only weak daily patterns. Our study underscores the flexibility in timekeeping mechanisms in Arctic species.

## Introduction

Various biological processes occur rhythmically, restricted to specific times of the day or season (Cassone 2014; Paajanen et al. 2025), and in many species are regulated by photoperiod—the duration of daylight—as a *Zeitgeber* (Walton et al. 2011). Maintaining these rhythms in polar regions is challenging because, above the Arctic and below the Antarctic circle (66°N and S), sunlight is entirely absent throughout the polar winter and continuously present throughout the polar summer (Vleck and Van Hook 2002). Nevertheless, various Arctic species can retain endogenous and behavioral rhythms (reviewed in Steiger et al. 2013; Williams et al. 2015).

Arctic vertebrates show variation in rhythmicity in behavioral activity between species, between individuals and within individuals. They are highly variable in their behavioral activity with some species (i) being completely *arrhythmic,* (ii) showing *circadian or diel rhythmicity,* ie displaying a recurring rhythm of approximately 24-hours even in the absence of light fluctuations, (iii) showing *ultradian rhythmicity* of recurrent periods of foraging and resting bouts less than 24-hours, or (iv) showing *free running rhythmicity,* ie not synchronized with environmental time cues (Steiger et al. 2013; Williams et al. 2015). For herbivores, Bloch *et al*. suggested that ultradian activity patterns in polar regions are linked with feeding behavior and digestive processes (Bloch et al. 2013). Between individual differences exist, for example, in migratory shorebirds displaying bi-parental care; conspecific pairs may show different incubation rhythms even if they breed in the same area (Steiger et al. 2013; Bulla et al. 2016). Furthermore, patterns may vary seasonally within individuals, eg driven by annual changes of day length (Bloch et al. 2013; Steiger et al. 2013). For example, the Svalbard rock ptarmigan (*Lagopus muta hyperborea*), the sole permanent resident bird on Svalbard, exhibits ultradian activity patterns with intermittent feeding during polar summer and winter days. In contrast, during spring and fall its activity patterns are diurnal and feeding mainly occurs within daylight hours (Stokkan et al. 1986). The diversity in behavioral responses to continuous daylight underlines the plasticity of the circadian system and indicates the relevance of the species’ biology (Steiger et al. 2013; Helm et al. 2017).

Behavioral and physiological processes are regulated through the rhythmic secretion of hormones by an endogenous network of one or several ‘Master’ clocks, which then coordinate and synchronize more peripheral clocks (reviewed in Cassone 2014; Williams et al. 2015). Crucial for tracking environmental cycles is the neurohormone melatonin. Its diel secretion pattern, ie an extended peak at night and basal secretion during the day, provides the paramount hormonal signal transducing day length for peripheral clocks (Walton et al. 2011). Melatonin secretion drives lasting changes in the hypothalamic-pituitary adrenal axis (HPA), where low levels of melatonin culminate in the increased secretion of the adrenal glucocorticoids, ie cortisol in mammals and corticosterone (CORT) in birds (Kalsbeek et al. 2012; Williams et al. 2015; Huffeldt et al. 2020). Glucocorticoids, which display robust diel rhythmicity in latitudes with year-round day-night cycles (Breuner et al. 1999; Quillfeldt et al. 2007; Romero and Remage-Healey 2000; but see Krause et al. 2015), are (i) involved in synchronizing peripheral clocks (Challet 2015), (ii) best known as being the downstream effectors of one of the two major stress response systems (Romero 2002), and (iii) interrelate with feeding, modulation of energy storage and mobilization as well as activity (Huffeldt et al. 2020). In temperate zones, the diel rhythmic pattern of baseline CORT correlates with activity; low levels of CORT during inactive phases are followed by a rise just before activity begins and CORT remains high as long as individuals are active (Huffeldt et al. 2020). Contrarily, in polar species during the summer, findings of a diel CORT rhythm are ambiguous. For example, common murres (*Uria aalge*) maintain a diel CORT rhythm in the polar summer (Huffeldt et al. 2021). In contrast, the link between activity, time of day and corticosterone is not evident in thick-billed murres (*U. lomvia*) (Huffeldt et al. 2020). Other species without a diel CORT rhythm are Adélie penguins (*Pygoscelis adeliae*) (Vleck and Van Hook 2002) and common eiders (*Somateria mollissima*) (Steenweg et al. 2015). Arrhythmicity in CORT was attributed to the suppression of melatonin secretion when photoperiod as *Zeitgeber* is absent (Steiger et al. 2013; Steenweg et al. 2015; Huffeldt et al. 2020). Also, the lack of a rhythm in CORT in common eiders was linked to CORT interfering with the need to constantly forage (Steiger et al. 2013), while in thick-billed murres it was ascribed to a stable modulation of energy storage and mobilization (Huffeldt et al. 2020).

Many bird species, which show rapid migration to their Arctic breeding grounds, experience large changes in daylight conditions en route, before they arrive in the polar regions with 24-hours of natural light. Diurnal migrants may benefit from extended daylight at higher latitudes, allowing more time for foraging and activity (Sockman and Hurlbert 2020). Nevertheless, such quick fluctuations in light conditions present a significant challenge for circadian physiology (Gwinner and Brandstätter 2001; Karagicheva et al. 2016; Eichhorn et al. 2021). There is a surprising variation in the response of migrants to continuous daylight (eg Steiger et al. 2013; Bulla et al. 2016), despite the fact that changing to a different rhythm may potentially be costly (Foster and Wulff 2005). To increase our understanding of the evolution of plasticity in circadian clocks in migratory birds, it is thus necessary to study rhythmicity in behavior and physiology in relation to the ecology of the species (eg Bloch et al. 2013; Bulla et al. 2016; Helm et al. 2017).

Here we aim to quantify behavior- and CORT-based rhythmicity in the barnacle goose, a migratory herbivore, during its Arctic breeding season, using a multifaceted approach. Earlier findings on rhythmicity in barnacle goose behavior and physiology are somewhat equivocal. A recent study showed that barnacle geese lost diel rhythmicity in body temperature once they encountered continuous daylight during their migration to the Arctic breeding sites (Eichhorn et al. 2021). During incubation in the Arctic, however, a diel rhythm in incubation recesses was described ( Prop et al. 1980; Prop and de Vries 1993; Tombre et al. 2012). Likewise, young barnacle geese retained a diel, albeit time-shifted, pattern of corticosterone metabolites (CORTm) determined from droppings, collected over 24-hours in their first summer (Scheiber et al. 2017). Here, we investigated potential diel and seasonal rhythmicity in activity and daily excretion patterns of CORTm in adult barnacle geese, using three different approaches; (i) activity and sleep during incubation in females, which were determined using photos from wildlife camera’s placed in the vicinity of the nest, (ii) activity of females and males across the breeding season, which was assessed using data from accelerometers (ACC), and (iii) CORTm concentration, which was determined from individually assigned droppings. Contrary to mammalian herbivores, where digestive processes dictate ultradian activity patterns, barnacle geese do not possess complex digestive systems (Prop and Vulink 1992; Black et al. 2014). However, their summer food retention time is increased by interrupting feeding with longer loafing spells (Black et al. 2014). We expected that barnacle geese would feed around the clock (sensu Eichhorn et al. 2021), which has been suggested to be a possible adaptation of Arctic herbivores to optimally utilize the peak in high quality food during the short summer (Hazlerigg et al. 2023). If indeed ultradian rhythmicity in activity is important, then we expected to find a weak or absent diel rhythm in CORTm (Steiger et al. 2013). In contrast, if barnacle geese rely on an evolutionary-based endogenous clock to schedule their biology or respond directly to external diel cues, then we predicted diel rhythmicity in activity and CORTm throughout the breeding season, similar to some other polar species (eg Ashley et al. 2014; Ware et al. 2020; Huffeldt et al. 2021).

## Materials and methods

### Study site and species

We collected data during the summers of 2020 to 2022 in a barnacle goose population nesting on islets in Kongsfjorden, Svalbard. Geese from this population migrate annually from their wintering grounds in the United Kingdom to Svalbard, stopping along the Norwegian coast (Black et al. 2014), and thereby experiencing a wide range of daylight conditions. Except on days with inclement weather when boating was not possible or polar bears (*Ursus maritimus)* were present, we monitored nests every other day during incubation and hatching on the two main breeding islands of the study area, Storholmen and Prins Heinrichøya, from June to the beginning of July (for details see de Jong et al. 2021). We determined the exact nest location using a handheld GPS (Garmin GPSmap 64S), noted clutch size and identified parents by their individually recognizable engraved plastic leg rings when the researcher approached the nest (de Jong et al. 2021). To minimize disturbance, we got information on possible nests of geese with transmitters (see below) on other islands, ie Midtholmen, Juttaholmen, Observasjonsholmen, from colleagues who worked there regularly. Egg laying and incubation in barnacle geese takes approximately 29 d (Lameris et al. 2019). After hatching, many geese raise their goslings in or in the direct vicinity of the village Ny-Ålesund (78°55′30″N 11°55′20″E; Loonen et al. 1998; Stahl and Loonen 1998). Here, we observed geese at least twice-daily using binoculars or spotting scopes to identify individuals by their color rings, to establish family size and record feather molt. From the first day of molt, the complete molting process lasts 35 to 40 d on average, with a flightless phase of ca. 25 d (Owen and Ogilvie 1979). In our analyses, we defined the flightless phase to start at the first observation of loss of all flight feathers, and to end 25 d later (from now on called “molt”).

In 2020, we fitted 12 females and 12 males from 24 pairs with black colored OrniTrack-NL40 3G transmitter neckbands (Ornitela, UAB, Lithuania. Mass ~20 gram, height 21 mm, diameter 38 to 40 mm). We caught ten geese on or near their nest on the islands Storholmen or Prins Heinrichøya during late incubation using two methods: (1) using a ~ 6 m hand-held noose pole to catch the bird around its neck, or (2) by catching the geese by hand, which was mainly possible for aggressive males that came close. We caught all remaining geese during annually performed catches, during which flightless molting geese are driven into corrals (Black et al. 2014). We chose the 24 individuals following a suite of criteria, including that it would be desirable if both pair partners were marked with unique color rings already (for details of criteria see Scheiber et al. 2025). We determined the sex of the birds from cloacal inspection. During the incubation phases in 2021 and 2022, we deployed additional transmitters on three birds (one female and two males) and four birds (three females and one male), respectively.

The study conforms to Directive 2010/63/EU and was conducted under FOTS ID 23358 from the Norwegian Animal Research Authority and approved by the Governor of Svalbard (RIS ID 11237).

### Activity during incubation and across the breeding season

#### Incubation patterns from wildlife camera photos.

To investigate rhythmicity during incubation, we set up wildlife cameras (Usogood TC30 Trail Camera) from 9/6/2021 to 16/7/2021 near the nests of incubating geese, which we had fitted with GPS transmitters in 2020 (n = 15). Cameras, set in time-lapse mode, took two pictures every five minutes. We ultimately retrieved the cameras either after hatching or when a nest was preyed upon or abandoned (Scheiber et al. 2025).

We analyzed pictures (n = 110739) with Timelapse2 Image Analyser (version 2.2.4.3, Greenberg et al. 2019). We quantified whether the female was (i) sitting on the nest, (ii) in ‘sleep posture’, ie head resting on back with beak often tucked under a wing (Dewasmes et al. 1985), (iii) standing right next to the nest or (iv) absent from the nest. For further analyses of nest recesses, we pooled behaviors (iii) and (iv) as *active* and behaviors (i) and (ii) as *inactive*. For the analyses of “sleep,” we contrasted being in the sleep posture with the other behaviors. Males sometimes nest sit (Scheiber et al. 2025), but here we focused on females only. We classified instances, where males nest sat, while females were on incubation recesses, as (iv) absent from nest.

In total, wildlife cameras supplied data from 2 h up to 20 d. We only used data which exceeded the recommended minimum duration of 10 d for biological rhythm analyses (Sokolove and Bushell 1978; Arnold et al. 2018). We could assess activity, ie incubation recesses, and sleep patterns, from photos taken by the wildlife cameras in 11 out of 15 females. We discarded the other four cases, because data were insufficient due to nest (n = 2) or camera failure (n = 2).

#### Seasonal activity from transmitter accelerometer data.

We used ACC data collected by the transmitters during the summers of 2021 and 2022 (2021: females n = 8, males n = 9; 2022: females n = 9, males n = 9; same individuals across 2021 and 2022: n = 12). We set the solar-powered transmitters to record a GPS-fix every 15 min when battery voltage was 75% to 100%, every 30 min at 50% to 74%, every 60 min at 25% to 49% or every 240 min at voltages lower than 25%. Immediately after each GPS-fix, the transmitter took a 2-sec ACC burst at a frequency of 20 Hz. Gravitational acceleration was measured in unit g/1000 in the three spatial axes (Schreven et al. 2021).

We used ACC data to identify activity and inactivity of individual geese during the breeding season (Dokter et al. 2018; Boom et al. 2023). For each goose, we checked the number of measurements per burst taken after each GPS-fix to ensure a complete dataset. Then, we calculated the vectorial sum of dynamic body acceleration (VeDBA) from the ACC data, a common proxy for energy expenditure (Qasem et al. 2012). For this, we first calculated static acceleration, ie the average raw measured acceleration for each dimension (x, y, z) within the bursts. Second, we subtracted the static acceleration from the raw data for each dimension, thus getting dynamic acceleration. Last, we calculated the vectorial sum of dynamic body acceleration of each burst by taking the square root of the summed dynamic accelerations of each dimension (Qasem et al. 2012). Following Boom et al. (2023), we created probability density histograms to identify peaks for activity and inactivity and used the *mix* function in the R-package *mixdist* to decompose the distribution into two gamma distribution components for active and inactive behavior (Macdonald and Du 2018). We found the threshold between the active and inactive behavior distributions by calculating the intersection point between the two (Fig. S1, Dokter et al. 2018; Boom et al. 2023).

In addition, we estimated the nesting phase based on the method described by Schreven et al. (2021) using VeDBA and GPS data. Nesting is defined as the entire duration of egg laying, nest building, incubation, and hatching. For females, we took daily median VeDBA < 1 for motionless days, as this corresponded best with observed nesting in the field (n = 14: nesting phase or part of the nesting phase could be estimated, n = 3: nesting phase could not be estimated due to transmitter attachment during hatching or when the goose was likely not breeding). We estimated the potential nest location by taking the median latitude and longitude of motionless fixes on days on which the goose was mostly stationary. We calculated nest site attendance as the distance of each GPS-fix of a goose to its potential nesting location and by subsequently calculating the daily amount of time that it spent within a 50 m radius of its potential nest site. We set the attendance threshold as the first day on which the goose spent > 75% of time within 50 m of the nest and the duration threshold at 3 d (Schreven et al. 2021).

Males were rarely motionless during nesting (n = 14), with the exception of four males. For these four, motionlessness could plausibly be used to establish the nesting phase. For nine additional males we established nesting based on field observations of the known nest location. For five males, however, neither method proved suitable, and we could not establish nesting.

In total, we obtained both an observed nest location in the field and an estimated nest location based on the transmitter data for 14 geese (males and females); the distance between the observed and estimated nest location was 2.37 m on average (SD = 0.98, range 0.85 to 3.67; we excluded an outlier of 25.45 m where the handheld GPS was likely turned on too late).

We based later breeding stages, ie when geese had goslings and/or were molting, on direct observations (see above). For 13 geese we obtained data on the phase with goslings, for 11 geese when they were molting and for three geese when they had goslings while molting (“goslings & molt” phase). Since these different breeding stages pose different demands on the geese (Black et al. 2014), we tested for rhythmicity within these three phases. Once again, we only used data, which exceeded the recommended minimum duration of 10 d for biological rhythm analyses. Therefore, we excluded four nesting phases, five gosling phases, one gosling & molt phase and one molt phase, because here data collection time was too short. We pooled the remaining two goslings & molt phases with the molt phase data to increase sample size.

### Noninvasive sampling of hormone metabolites: Sample collection and corticosterone metabolite assay

Although physiological stress in birds has been determined by measuring corticosterone levels from blood (eg Hoarau et al. 2022), sampling various other body fluids (eg sputum, semen) and excretion products (feces, urine) has become a suitable alternative, particularly if capture and blood collection may not be logistically feasible (Washburn et al. 2003). To draw valid conclusions, however, when determining corticosterone metabolite levels a careful validation is required beforehand (Touma and Palme 2005; see Scheiber et al. 2017 for details on validation in barnacle geese). Contrary to sampling blood, which shows concentrations that occur in a very short time frame, hormone metabolites from excreta show integration of corticosterone levels over a certain time, ie until the hormone has been metabolized (Scheiber et al. 2005). Activities related to catching and drawing blood may increase corticosterone levels fast, conventionally < three minutes (Romero and Reed 2005), which could mask meaningful stress determination for the research in question. In earlier studies in geese, CORTm was not only used to determine acute stress responses (Scheiber et al. 2018), effects of legacy trace metal contamination (Scheiber et al. 2018), but also diel rhythmicity (Dorn et al. 2014; Scheiber et al. 2017). Furthermore, CORTm was also used to determine seasonal changes (see Touma and Palme 2005 for a review) in mourning doves (*Zenaida macroura*) (Washburn et al. 2003), Northern bald ibis (*Geronticus eremita*, Dorn et al. 2014) and greylag geese (*Anser anser*, Kotrschal et al. 1998, 2000; Frigerio et al. 2003, 2004; Dorn et al. 2014).

To investigate diel rhythmicity of CORTm excretion, we collected droppings of ringed geese in the village of Ny-Ålesund from 1/7/2020 to 13/8/2020 (n = 349 droppings, 26 individuals; 14 females, 12 males) and 16/6/2021 to 31/7/2021 (n = 333 droppings, 52 individuals; 28 females, 24 males). For 17 individuals we collected samples in both years. We attempted to collect a minimum of three samples (Scheiber et al. 2005) per 3-hour time intervals, ie from 0:00 to 2:59, 3:00 to 5:59, . . . , 21:00 to 23:59 and intended to cover one complete 24-hour cycle every week for each individual. This was often not possible, as pairs sometimes left the area and could not be located. We collected only unambiguously assigned samples and froze these at −20°C within 1 h (for methodology on how to collect droppings, see Supporting Information). We shipped the droppings frozen to the Dept. of Behavioural and Cognitive Biology, University of Vienna, Austria, for analyses.

We quantified dropping samples for determining CORTm using an enzyme immunoassay validated for barnacle geese (Scheiber et al. 2017) and applied successfully in previous studies (eg Scheiber et al. 2018). We defrosted samples in the lab and weighed them in at 0.5 g of wet dropping material. We further followed the protocol as described in Scheiber et al. (2017). Cross-reactivities of the assay are provided in Frigerio et al. (2004). CORTm was below the detection limit in three samples (total n = 679). Determined from homogenized pool samples, intra- and inter-assay coefficients of variation (% CV) were 11.91% (< 15%) and 13.03% (< 25%), respectively.

### Statistical analyses 

We performed all analyses in R version 4.4.0 (R Core Team 2024) .

#### Rhythmicity in activity.

We plotted actograms to visualize overall activity patterns during incubation, as assessed from wildlife camera data, and across the entire breeding season as determined from transmitter data (*ggetho* package: Geissmann et al. 2019). We double-plotted actograms, which show rhythmicity or a lack thereof, to facilitate inspection. We tested for periodicity in actograms using Lomb-Scargle periodogram (LSP) analysis (*periodogram* function, *zeitgebr* package: Ruf 1999; Geissmann et al. 2019) during specific times: ie the limited time frame between camera placement during incubation until we spotted the first gosling on an image, and the entire nesting phase (defined as the total time of nest building, egg laying, incubation, and hatching) as well as gosling rearing and molt for the transmitter data. The LSP analysis was suitable to discover periodicity in our dataset as it can handle unequally sampled time-series with missing data (Ruf 1999). Periodicity, ie the regular recurrence of a behavior or an event over fixed, equal time intervals, is a common but not universal trait of rhythmicity, which in itself emphasizes the repetitive pattern of changes rather than precise timing (Hogan and Sternad 2007). For each dataset we ran two LSP analyses; one focused on ultradian rhythmicity with a period range between 1 and 18 h and another focused on diel rhythmicity with a period range between 18 and 36 h (following Arnold et al. 2018; van Beest et al. 2020; Ware et al. 2020). Data were re-sampled every 5 minutes in the case of data from nest cameras and every 15 minutes for data provided by the transmitters. The oversampling rate was set at 100. We used the *find_peaks* function from the *zeitgebr* package to extract significant peaks (p < 0.05). Time-series data can contain two separate peaks, which may either point to a true combination of ultradian and diel rhythmicity or it can be a data artifact (Aschoff 1966). We followed the methods of van Beest et al. (2020) to distinguish between these two: if we found two peaks in the normalized power for the same individual with an 18 h window we rejected the smaller peak if it was less than one-third the height of the larger one.

We investigated possible differences in ultradian and diel peak periods between breeding stages (nesting, gosling and molt) using linear mixed-effects models (*lme* from the package *nlme*: Pinheiro and Bates 2000, 2024) with peak period in hours as the response variable and breeding stage (categorical) as a predictor variable. In addition, we also considered the predictor variables sex (categorical: female, male), the interaction between sex and breeding stage, and year (categorical). We accounted for repeated measures by adding ID as a random intercept. We used automated model selection using the function *dredge* (package *MuMIn*) and AICc (Bartoń 2024). In the Supporting Information we give the 95% confidence set of models (Symonds and Moussalli 2011) and statistics including 85% confidence intervals for the full model in the text (Sutherland et al. 2023). The 85% interval is consistent with how variables are selected using AIC. When present in the top model, we computed estimated marginal means for specific factors and comparisons among levels (package *emmeans*: Lenth 2024).

#### Diel patterns of immune-reactive corticosterone metabolites.

To investigate the association between predictor variables and CORTm concentration over the day, we fitted linear fixed-effects models (*lme*). We log-transformed CORTm concentration to adhere to model assumptions. The predictor variables in all models include time of day (continuous), year (categorical), day of the year (continuous) and sex (categorical). As time of day is a circular variable, we changed it into two linear variables by first transforming hour of day to radians and then calculating the sine and cosine of those radians. We included sine and cosine as continuous predictor variables in our models (Pewsey et al. 2013; Huffeldt et al. 2021). To account for repeated measures of the same individuals, we added individual identity as a random intercept in all models. A more complex random slope model could not be explored, because this fitted mixed model was singular. As above, we used model selection using the function *dredge* (package *MuMIn*) and AICc and give the same model information.

#### Post hoc analysis on locations of geese during the gosling and molt phase.

In the above-mentioned analyses, we detected patterns in activity behavior during molt that seemed to resemble tidal rhythms (see Results). Ny-Ålesund has a semi-daily tide with a period of ~12.4 h (Norwegian Hydrographic Service; see Figure S47 for a rhythmicity plot of the observed water level over the season). We also increasingly observed geese foraging in the intertidal area during molt (pers. obs.). To investigate in more detail whether geese rhythms coincided with the tides, we estimated the number of GPS coordinates on land and in the intertidal area/on sea during the gosling and molt phases, to see if geese indeed switched to this area. We downloaded the land shapefile of a 1:100 000 map of Svalbard (Norwegian Polar Institute 2014) and set a buffer zone of 25 m inside the polygon to account for geese resting on the beach during high tide after they foraged in the intertidal zone at low tide (package *sf*: Pebesma 2018). After reprojecting the goose GPS data to match the shapefile coordinate reference system, we calculated if GPS points fell within or outside the buffered polygon and counted the number of points. We used a generalized linear mixed model (GLMM: *glmer* function from the package *lme4*: Bates et al. 2015) with a binomial error distribution to investigate whether the likelihood of an individual being located outside the buffer differed between molt and gosling phases. The response variable was defined as the number of GPS points outside vs inside the buffer (“cbind(outside points, inside points)”). The model included the breeding stage (molt vs goslings) as a fixed effect and individual ID as a random intercept to account for repeated measures within individuals. We checked model assumptions and calculated 95% confidence intervals (CIs) for the fixed effects using parametric bootstrapping with 1,000 iterations. For each iteration, we resampled the dataset with replacement, refitted the model, and extracted the fixed-effect estimates. We set a random seed (“function set.seed(123)”) before bootstrapping, to ensure reproducibility.

## Results

### Rhythmicity in activity

#### Rhythmicity during incubation.

**Fig. 1.**
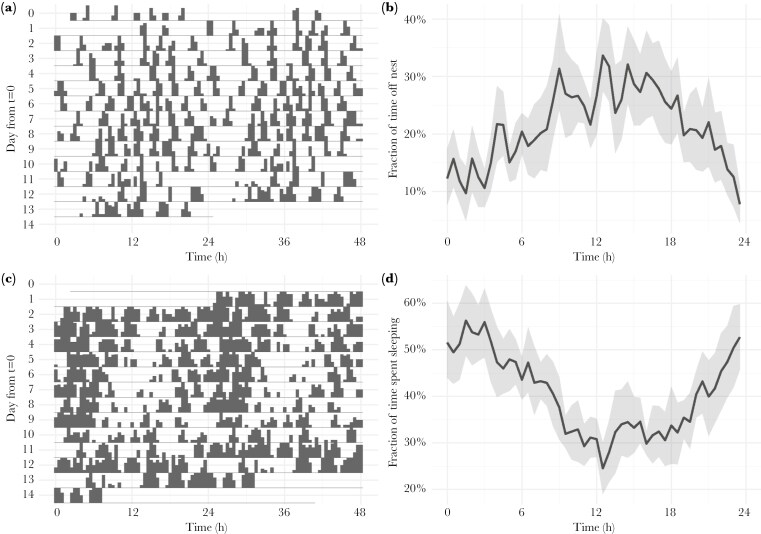
Rhythmicity of females during incubation recesses [panels (A) and (B)] and ‘sleep’ [panels (C) and (D)] based on wildlife camera pictures. Double plotted actograms of female ID CA41113 as an example show in black when she is away from the nest during incubation recesses (A) or when she is on the nest in the sleeping posture (C), while transparency indicates inactivity on the nest in (A) or any behavior other than sleep in (C). The height of the bars indicates how often the specific behaviors are observed during that time. The x-axis displays two consecutive days, and these consecutive days are also shown from top to bottom on the y-axis. On the y-axis t = 0 is the day the first pictures were taken by the wildlife camera on 2021-06-18 from 03:22:20 onwards. Population level (N = 11) graphs show the fraction of time, averaged over 24 h, spent on incubation recesses (B) and in sleep posture (D) in percentages (y-axis). The x-axis displays time over 24 h, and the black line represents the mean, while in gray bootstrapped 95% confidence intervals are displayed.

All females showed rhythmicity in incubation recesses (Fig. 1A, for actograms of all females see Figure S2). Ten females showed a combination of both ultradian and diel rhythmicity while one female showed ultradian rhythmicity only (Table S1, Figure S3). The mean ultradian period in incubation recesses was 3.21 h (95% CI: 2.41, 3.89) and the mean diel period was 24.03 h (95% CI: 23.73, 24.38). The highest fraction of activity across all individuals occurred between ~ 12:00 and 16:00, when the average activity level reached approximately 29% (Fig. 1B). In addition, in nine females we found both ultradian and diel rhythmicity when resting in a sleep posture (Fig. 1C, for actograms of all females see Figure S4), while two females showed diel rhythmicity only (Table S2, Figures S5). The mean peak ultradian period was 8.17 h (95% CI: 5.14,10.64) and the diel period was 24.96 h (95% CI: 23.57,1 26.12). Overall, the highest fraction of sleep across all individuals occurred between ~ 01:00 and 03:00, when this was approximately 53% on average (Fig. 1D).

#### Seasonal rhythmicity in activity.

**Fig. 2.**
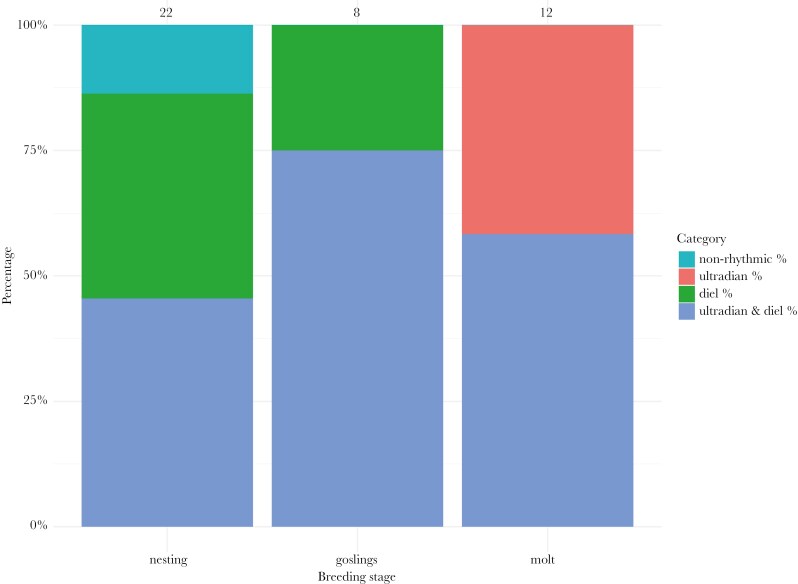
Percentages of rhythmicity types based on transmitter accelerometer data for 18 individual geese over the course of two summer seasons. Over the three breeding stages either ultradian or diel rhythmicity alone, a combination of ultradian and diel rhythmicity, or no rhythm were observed. Numbers above the stacked bars indicate sample size per breeding stage.

**Fig. 3.**
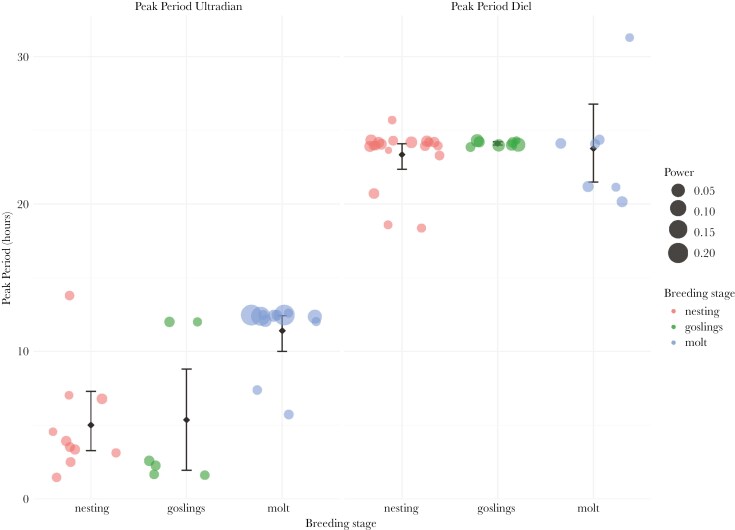
Diel and ultradian average peak periodicity during different breeding stages based on transmitter accelerometer data. The values of the peak period are for rhythmic geese only, ie a peak above the significance threshold at α = 0.05. Individual geese are shown as dots. The size of the dots represents the power of the peak period; a representation of the strength of the signal. The error bars are 95% bootstrap confidence intervals on the population mean.

**Fig. 4.**
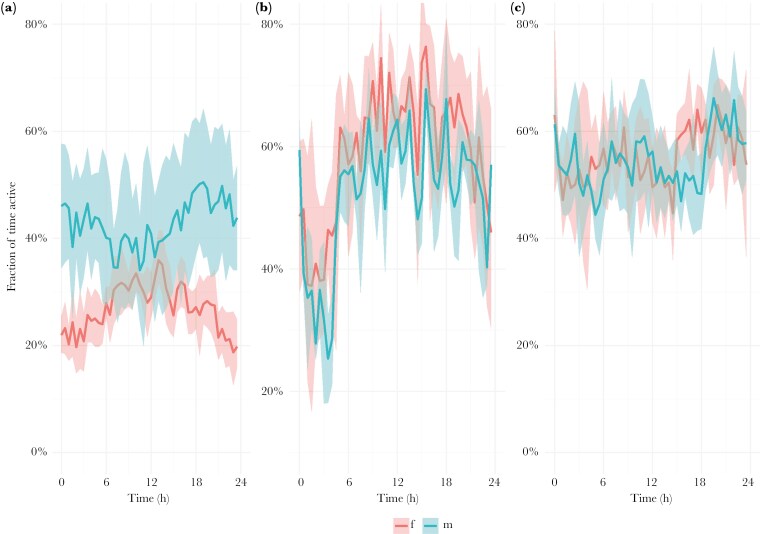
Population level graphs, based on transmitter accelerometer data, showing the fraction of time active, averaged over 24 hrs., during nesting (A), when geese had goslings (B), and during molt (C) in percentages (y-axis). Females (f) and males (m) are indicated in different colors. The x-axis displays time over 24 h, and the solid lines represent the mean, while in transparent color bootstrapped 95% confidence intervals are displayed.

We detected rhythmicity in all geese over the course of the entire breeding season (for actograms of all geese see Figures S6-40). Except for three females, which showed no rhythmicity in activity patterns during nesting in 2022, most geese showed diel rhythmicity or a combination of ultradian and diel rhythmicity during nesting and when they had goslings (Fig. 2 left and center panel, for periodograms of all geese see Figures S41-46). Furthermore, we also detected either ultradian rhythmicity only or a combination of ultradian and diel rhythmicity during molt (Fig. 2 right panel). The mean ultradian period in activity was 5.01 h (95% CI: 2.67, 6.75) during the nesting phase, 5.36 h (95% CI: 1.90, 8.76) during the gosling phase and 11.41 h (95% CI: 10.39, 12.83) during the molt phase (Fig. 3 left panel, see Figures S41-43 for individual differences). In addition, the mean diel period in activity was 23.35 h (95% CI: 22.58, 24.22) during the nesting phase, 24.11 h (95% CI: 24.00, 24.21) during the gosling phase and 23.76 h (95% CI: 20.76, 25.90) during the molt phase (Fig. 3 right panel, Figures S44-46). We found evidence that breeding stage correlated with the ultradian peak period, as this variable was present in all models within the 95% confidence set (Table 1A, Table S3). The top model included breeding stage only (Table 1A) and post-hoc testing revealed that the ultradian peak period during molt was significantly higher than when geese nested or had goslings (Fig. 3, gosling—molt; estimate = −6.05, SE = 1.75, p = 0.015, gosling—nesting; estimate = 0.35, SE = 1.80, p = 0.9794, molt—nesting; estimate = 6.40, SE = 1.49, p = 0.004). There was no strong indication that breeding stage influenced diel peak period, with the top model being the null model (Table 1B, Fig. 3, see also Table S4). The fraction of time when geese were active, varied between males and females during nesting, with females being more active around noon, while males showed an opposite pattern (Fig. 4A). When geese had goslings or were molting, females and males did not differ in the fraction of time they were active (Fig. 4B & C). During the gosling phase there was a sharp decrease in activity after midnight until early morning. This pattern was not present during molt.

**Table 1.** Intercept and coefﬁcient estimates from the full models investigating differences in (A) ultradian peak period in hours and (B) diel peak period in hours with corresponding standard errors (SE), 85% Confidence intervals (lower, upper), and if the variable was selected in the top AIC model. For (A) the random intercept for individual ID had a standard deviation of 0.0001 and the residual standard deviation was 2.96. For (B) the random intercept for individual ID had a standard deviation of 0.87 and the residual standard deviation was 1.76.

Variable	(A) Estimate	SE	Lower	Upper	AIC top	(B) Estimate	SE	Lower	Upper	AIC top
**Intercept**	7.30	2.42	4.11	10.49		24.03	1.12	22.52	25.53	
**Breeding stage—molt**	5.79	2.89	1.74	9.83	Yes	−2.26	1.62	−4.50	−0.03	No
**Breeding stage—nesting**	−2.18	2.96	−6.33	1.97	Yes	−0.43	1.29	−2.21	1.35	No
**Sex—Male**	−2.91	2.96	−6.82	0.99	No	−0.22	1.56	−2.32	1.89	No
**Year—2022**	−3.53	1.93	−6.23	−0.84	No	−0.22	0.86	−1.39	0.96	No
**Breeding stage—molt: sex—male**	1.04	3.58	−3.97	6.05	No	3.84	2.21	0.79	6.89	No
**Breeding stage—nesting: sex—male**	4.49	3.82	−0.85	9.84	No	−0.22	1.80	−2.70	2.26	No

### Diel patterns of immune-reactive corticosterone metabolites (CORTm)

**Fig. 5.**
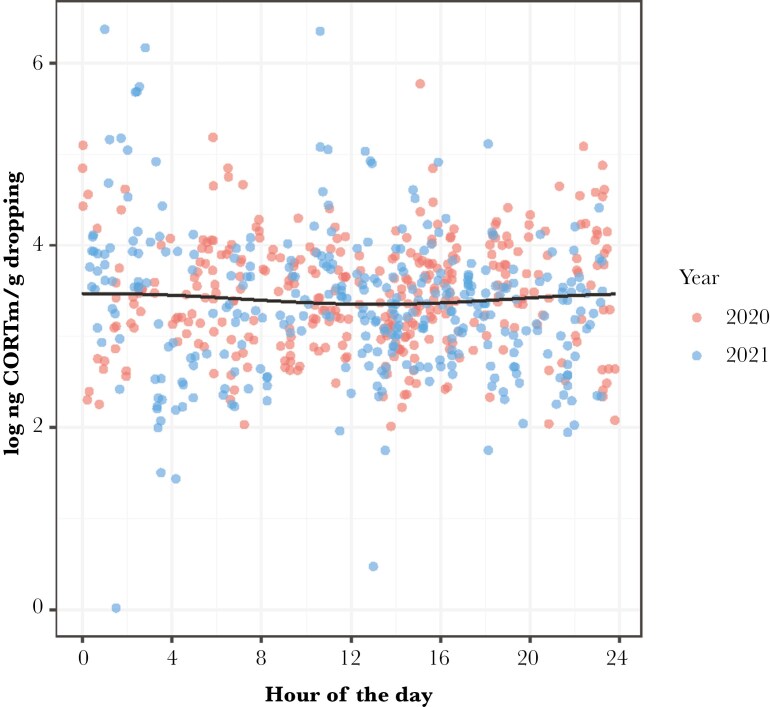
Daily rhythmicity in log-transformed corticosterone metabolite (CORTm) concentration measured in barnacle goose droppings in 2 yr. The dots represent individual measurements, and the solid line represents the predicted values from the model. 2020: n = 349 droppings, 26 individuals. 2021: n = 333 droppings, 52 individuals.

Our results revealed that CORTm concentration was marginally influenced by time of day (Table 2), with both cosine and sine of time of day being present in the top model as well as in many models within the 95% conﬁdence set (Table S5). CORTm concentrations slightly increased during the night, peaking around 02:00. Concentrations declined throughout the daytime, reaching a minimum around 14:00 (Fig. 5). CORTm concentrations were slightly different between the years (Table 2; 2020 median: 32.13 ng/g dropping, range: 7.49 to 320.9 ng/g; 2021 median: 28.56 ng/g dropping, range: 1.02 to 583.56 ng/g). In addition, over the course of the season CORTm concentration slowly declined (Table 2). Collected mostly late at night on one specific day, several samples contained very high levels of CORTm, ie > 200 ng CORTm/g droppings (n = 7). Omitting these data points in a second analysis revealed that the outcome did not differ substantially, except that the top model now only included the cosine of time of day and year (Tables S6, S7).

**Table 2.** Intercept and coefﬁcient estimates from the full model investigating rhythmicity in log-transformed corticosterone metabolite concentration with corresponding 85% CIs, and if the variable was selected in the top AIC model. The random intercept for individual ID had a standard deviation of 0.23 and the residual standard deviation was 0.67.

Variable	Estimate	Standard error	Lower	Upper	AIC top
**Intercept**	5.03	0.45	4.38	5.67	
**Cosine**	0.1	0.04	0.05	0.15	Yes
**Sine**	0.06	0.04	0.003	0.11	Yes
**Sex—male**	−0.08	0.09	−0.21	0.05	No
**Year—2021**	−0.2	0.06	−0.29	−0.11	Yes
**Year day**	−0.007	0.002	−0.01	−0.004	Yes

### Post hoc analysis on locations of geese during the gosling and molt phases

When comparing the locations of the geese during the gosling and molt phases, we found that, on average, 7.05% (SD 4.99%) of goose locations were in the intertidal area during the gosling phase, while this increased to 70.2% (SD 27.6%) during molt (GLMM: intercept β = −3.02, SE = 0.59, z = −5.11, 95% CI [−3.76, −2.29]; breeding phase molt β = 4.51, SE = 0.79, z = 5.69, 95% CI [3.32, 5.73]. Random intercept variance was 2.76 (SD = 1.66). For maps see Figure S48).

## Discussion

Svalbard barnacle geese maintain rhythmicity in behavior and physiology during their Arctic breeding season. We detected that most females showed a combination of both ultradian and diel rhythmicity in incubation recesses and sitting in a sleep posture on the nest. Most females and males also showed a combination of ultradian and diel rhythmicity or diel rhythmicity only in activity during their entire nesting phase or when they had goslings. During molt, geese exhibited either ultradian rhythmicity alone or a combination of ultradian and diel rhythmicity in their activity, possibly indicating a response to tidal rhythms (discussed further below). We detected no differences in peak periodicity between females and males across the various breeding stages. However, during the nesting phase, females and males exhibited contrasting activity patterns, with females leaving their nest more often around noon, while males were less active then. Furthermore, the geese showed weak diel rhythmicity in excreted corticosterone metabolites with concentrations increasing during the night and decreasing during the day. Below we discuss our findings in more detail.

### Ultradian rhythmicity in goose activity behavior

Our results indicate the significance of ultradian rhythmicity, ie periods of less than 24 h, in activity behavior of barnacle geese over the breeding season. During nesting and when geese had goslings, they showed ultradian activity rhythms with a period of ~ 5 h (Fig. 3). This ultradian rhythm might display a foraging activity—resting pattern (Prop et al., 1978), as is also shown by other Arctic herbivores such as Svalbard ptarmigan, Svalbard reindeer (*Rangifer tarandus platyrhynchus*) and muskoxen (*Ovibos moschatus*) around the summer solstice (eg Bourguignon and Storch 2017; Arnold et al. 2018; van Beest et al. 2020; Appenroth et al. 2021). Such an activity pattern could be endogenously generated by ultradian oscillators (Bourguignon and Storch 2017) or controlled by the interplay of feeding and digestion (Bloch et al. 2013; Hazlerigg and Tyler 2019).

### Diel rhythmicity in goose activity behavior

Many geese also showed diel activity patterns in behavior, ie periods of around 24 h, throughout the breeding season. The presence of a diel rhythm does not inherently imply, however, that circadian systems are functional and/or entrained as the geese could be reacting directly to external cues, also known as “masking” (Aschoff and Tokura 1986; Williams et al. 2015). For example, during nesting, female geese may respond adaptively to subtle environmental rhythms, such as temperature differences between the ‘subjective day and night’, with slightly higher temperatures typically occurring during the day despite 24h daylight. This might limit egg cooling rates during incubation recesses, as shown in snow geese (*Anser caerulescens*; Poussart et al. 2001). Males do not incubate, although they sometimes sit on the nest (Scheiber et al. 2025), but often stand guard close by the nest when the female is on incubation recesses (Prop et al. 1980), which may explain the opposite pattern in activity in males (Fig. 4A). Nests, however, are more vulnerable to predation if the female is on recess (Prop et al. 1984) with the most significant egg predators, glaucous gulls (*Larus hyperboreus*) and Arctic skuas (*Stercorarius parasiticus*), being more active during subjective daytime (Tombre et al. 2012). This conflicts with females being on recesses then. Arctic foxes (*Aleopex lagopus*), on the other hand, are usually more active during the subjective night (Tombre et al. 2012; pers.obs.). While foxes have been present in the gosling rearing areas during the study years, no foxes have been observed on the islands—where nests were monitored—for more than a decade (pers. obs.). Although barnacle geese may not be able to protect their nests against foxes even if both parents are present (Madsen et al. 1992; Tombre et al. 1998), fox predation was suggested to be the selective force of the observed diel rhythmicity during incubation (Tombre et al. 2012) as a ‘ghost of competition past’. If the day-active behavior of female geese during nesting is shaped by an expected daily rhythm in fox predation risk, it suggests the activity pattern is regulated by an endogenous, innate circadian mechanism (Kronfeld-Schor et al. 2017). It is feasible that a goose’s circadian clock then continues to function by employing alternative potential *Zeitgeber*s, such as diel changes in light intensity, polarization patterns, solar azimuth, UV radiation, changes in the spectral composition of light or slight changes in ambient temperature, which exist under polar summer conditions (Williams et al. 2017).

### Ultradian and diel rhythmicity in sleep

Studies on sleep patterns under natural conditions are rare due to challenges in measuring sleep in wild free-moving animals (Rattenborg et al. 2017). During incubation, we scored sleep posture, which has been associated with rapid eye movement (REM) sleep and non-REM (NREM) sleep in domesticated geese (Dewasmes et al. 1985). We, therefore, assume that our behavioral scoring of sleep was valid overall, although misclassifications may have occurred if individuals fell asleep while sitting with their beaks pointing forwards or were awake in the typical sleep posture. In addition, various bird species, such as mallards (*Anas platyrhynchos*) for example, perform unihemispheric sleep (Rattenborg et al. 1999), which we could not measure here. The diel sleep pattern contrasts distinctly with the diel pattern observed in nest recesses (Fig. 1), with females sleeping more between ~ 01:00 and 03:00. In addition, we detected ultradian rhythmicity, with a mean peak period of ~ 8 h. Awakening bouts could serve as a periodic screening of the environment for danger (Rattenborg et al. 1999; Voss 2004). Captive barnacle geese kept under a natural day—night cycle also showed scattered sleep over the course of the day during summer (van Hasselt et al. 2021), and the authors proposed that barnacle geese might have an attenuated circadian organization and may profit from becoming arrhythmic during the polar summer. We found no strong evidence for arrhythmia in incubating females, however. In future studies, it would be interesting to study sleep patterns throughout the season in both sexes.

### Tidal rhythm in goose activity during wing molt

During wing molt, barnacle geese are most vulnerable to fox predation (Prop et al. 1984). At times, when goslings are still present, parents are forced to forage in areas with high quality food even during molt, thereby risking higher predation (Stahl and Loonen 1998). When goslings are preyed upon, adults can retreat to areas with lower-quality food but higher safety, such as tundra lake shores with mossy vegetation (Stahl and Loonen 1998). Based on GPS data and observations, we found molting geese without young increasingly utilizing the intertidal area to rest and/or forage on algae in comparison to when they still had goslings (Figure S48). The ~ 12.4 h ultradian rhythmicity patterns of most geese during molt might thus reflect the falling and rising tides (Fig. 3; Bulla et al. 2017). The tides pose a constraint on an all-day activity (Klaassen et al. 2010; Pokrovsky et al. 2021), but the nearby sea provides safety from foxes. Although barnacle geese were shown to avoid salt-marsh vegetation in spring when it was experimentally sprayed with seawater, because they presumably are less capable of physiologically coping with very high salt loads in their environment (Stahl et al. 2002), molting geese foraged on algae in our study, possibly benefitting from fresh river water nearby. Molt in geese is energetically costly, because they molt wing coverts, some body feathers and flight feathers simultaneously. To compensate, geese become less active overall (Fig. 4C, Owen and Ogilvie 1979). Unfortunately, sample sizes of geese with goslings were too small to study if these, in contrast, retain their activity rhythm throughout molt.

### Weak diel rhythmicity in corticosterone metabolites (CORTm)

Besides rhythmicity in activity, geese also maintained an, albeit weak, rhythmic pattern of excreted CORTm (Fig. 5), similar to what we found in human-raised barnacle goslings over 24 h (Scheiber et al. 2017). Glucocorticoids are integral in providing physiological signals to regulate biological rhythms in sync with daily environmental cycles linked with activity and feeding (Kalsbeek et al. 2012; Challet 2015). During the gosling phase, when we collected most samples for CORTm measurements, our finding contradicts this general assumption, because values dropped from midnight to being lowest around midday, when geese were most active. As discussed in an earlier study (Scheiber et al. 2017), increased fox activity at night, during the time when the goose families preferentially rest, could have affected the CORTm rhythm. Furthermore, we detected a slight difference in overall CORTm concentrations between years, but we can only speculate of why this variation has arisen. CORTm can be influenced by eg weather (Krause et al. 2016) and predation pressure (Noreikiene et al. 2021), but the season in which we measured slightly higher CORTm concentration in 2020, seems to have been relatively milder in these aspects than 2021 (pers. obs.). One possibility is that in 2020 there were fewer people in the village due to COVID-19 restrictions. This may have reduced potential disturbance by humans. However, we lack detailed data to investigate whether this was indeed a factor. In sum, whether the attenuated rhythm in CORTm is sufficient to regulate activity patterns and other functions during the polar summer or whether it plays only a minor role in endogenous timekeeping (Huffeldt et al. 2021) needs to be investigated in the future. On a methodological issue, we like to note that fecal cortisol/corticosterone metabolites reflect biologically active circulating free glucocorticoids (Sheriff et al. 2010; Fauteux et al. 2017; Palme 2019), which are mostly metabolized in the liver. Glucocorticoids, which are bound to corticosteroid-binding globulin (CBG) are biologically inactive, but may serve as a repository to be quickly converted into active hormones in case of need (Palme et al. 2005; Malisch and Breuner 2010). If the influence of CBGs or receptors varies over the diel cycle (eg Breuner and Orchinik 2002; Dickmeis 2009) along with changes in the metabolism and excretion of metabolites (Palme 2019), these mechanisms may ensure that corticosterone metabolite levels in feces remain stable across the diel cycle, despite fluctuations in their physiological impact (Huffeldt et al. 2021).

### Suitability of the methods used

Our use of a multifaceted approach, combining behavioral observations via wildlife cameras and accelerometers with corticosterone measurements, allowed for a comprehensive understanding of rhythmicity in barnacle geese during their Arctic breeding season in 24h daylight. We found accelerometers to be a suitable alternative for investigating activity, where time consuming direct behavioral observations are difficult. They are a valid substitute also for pictures obtained from wildlife cameras, where a multiplicity of photos needs to be analyzed in detail afterwards. For example, we found that the peak period in activity of five females during incubation, identified from both wildlife cameras and accelerometers corresponded very well. Except for one female, where we found diel rhythmicity retrieved from the ACC data, which we did not detect in data from the wildlife cameras, the ACC-based activity patterns gave reliable results in the four remaining individuals, despite the slightly longer 15 min intervals of ACC measurements relative to the 5 min intervals of subsequent photos (ultradian: camera data; mean = 3.5 h, SD = 1.7, ACC data; mean = 3.8 h, SD = 1.7. diel: camera data; mean = 23.8 h, SD = 0.7, ACC data; mean = 24.2 h, SD = 0.1). This indicates that ACC-based data described activity very reliably. This is supported by another study in captive barnacle geese, which investigated sleep—wake rhythms. Here, activity measurements from ACC data correlated well with patterns retrieved from electroencephalograms (van Hasselt et al. 2021). Contrary to our study, where ACCs were mounted in neck collars, these ACCs were head mounted. They took high-frequency measurements at a high sampling rate of 100 Hz, and could, thus, record even small head movements. In our study data were less fine-tuned, because they were collected at a lower rate, ie every 15 min during 2-sec bursts of 20 HZ.

## Conclusion

Over the course of their Arctic breeding season, barnacle geese show some degree of plasticity in their daily rhythms. Such intra-individual shifts in rhythms between sexes and breeding stages also exist in shorebirds (Steiger et al. 2013; Bulla et al. 2016). One possible explanation is that in barnacle geese the circadian clock mechanism keeps ticking, but the control, which it exerts over behavioral output, is plastic and is applied only when it provides some advantage, possibly similarly to what was found in reindeer (Meier et al. 2024). Alternatively, the observed rhythms are not endogenously controlled, and geese respond directly to environmental or social cues, such as temperature, predation pressure, tides and/or their mates’ or goslings’ activity. The investigation of individual plasticity and consistency in behavioral and hormonal rhythmicity of Arctic migrants in the light of rapid environmental change, and their relationship with fitness, are promising avenues for future work.

## Supplementary Material

araf071_suppl_Supplementary_Materials

## Data Availability

Analyses reported in this article can be reproduced using the data provided by de Jong et al. (2025).
