# Supplementary material for: Diel rhythmicity of activity and corticosterone metabolites in Arctic barnacle geese during breeding
Source: Behav Ecol. 2025 Jun 13;36(4):araf071. doi: 10.1093/beheco/araf071 (PMC12211741; doi:10.1093/beheco/araf071)
Supplement: araf071_suppl_Supplementary_Materials [file araf071_suppl_supplementary_materials.pdf]

Supplement: Material and methods

Dropping collection

We collected individual droppings for determination of corticosterone metabolites (CORTm) by following individual geese from a distance with a telescope. The scope was fixed on the exact spot at which a goose defecated. As soon as geese voluntarily left the area, the observer was guided to this spot by a second person using handheld VHF radios. It was relatively easy to locate the correct droppings, because of the shortly grazed tundra vegetation with small rocks, flowers *etc.* as reference points, and because new droppings are still wet relative to other dried droppings in the vicinity. In cases of uncertainty to assign a dropping correctly, it was left behind.

Supplement: Tables

Incubation behavior

Table 1: Results of the Lomb-Scargle periodogram analysis on ***incubation recesses****, extracted from wildlife camera pictures. Level of significance is set to $P < 0.05$. One goose did not show a significant diel peak period.*

**Goose ID**	**Peak period ultradian (h)**	**Peak power ultradian**	**Significance threshold ultradian**	**Peak period diel (h)**	**Peak power diel**	**Significance threshold diel**
CA41113	3.63	0.02	0.005	23.70	0.03	0.003
CA41238	2.78	0.02	0.004	24.22	0.03	0.003
CA41400	6.48	0.02	0.004	22.78	0.02	0.003
CA44228	3.49	0.01	0.004	24.37	0.02	0.003
CA45632	3.60	0.03	0.005	23.74	0.05	0.004
CA45701	3.62	0.02	0.004	24.01	0.02	0.003
CA45736	1.76	0.03	0.006			
CA45744	3.43	0.02	0.005	24.32	0.02	0.004
CA45891	2.27	0.03	0.005	23.81	0.04	0.003
CA46848	1.94	0.02	0.005	24.64	0.02	0.003
CA45997	2.27	0.02	0.005	24.67	0.02	0.004

Table 2: Results of the Lomb-Scargle periodogram analysis on ***sleep posture****, extracted from wildlife camera pictures. Level of significance is set to P < 0.05. Two geese did not show significant ultradian peak periods.*

Goose ID	Peak period ultradian (h)	Peak power ultradian	Significance threshold ultradian	Peak period diel (h)	Peak power diel	Significance threshold diel
CA41113	3.63	0.02	0.004	23.75	0.05	0.003
CA41238	2.78	0.01	0.003	23.07	0.02	0.002
CA41400	6.45	0.01	0.004	23.87	0.03	0.003
CA44228	12.37	0.02	0.004	23.82	0.02	0.003
CA45632	5.98	0.03	0.005	26.46	0.03	0.003
CA45701	12.45	0.02	0.004	23.87	0.03	0.003
CA45736	4.64	0.02	0.006	26.66	0.03	0.004
CA45744	9.96	0.01	0.004	30.87	0.02	0.003
CA45891				23.12	0.07	0.003
CA46848				23.87	0.04	0.003
CA45997	15.37	0.03	0.004	25.21	0.05	0.003

Seasonal rhythmicity in activity

Table 3: The 95% confidence set of models investigating differences in ***ultradian peak period in hours in activity behavior during three breeding stages*** *(nesting, gosling, molt periods) within a cumulative Akaike weight of ≥ 0.95 from the top model. The '+' indicates which variables are present in the different models, delta gives the difference in AICc between the models, and weight represents the relative probability that a certain model is best compared with a set of models, based on the data and taking model complexity into account.*

Intercept	sex	breeding stage	year	sex : breeding stage	df	logLik	AICc	delta	weight
5.36		+			5	-73.14	159.01	0.00	0.39
5.36		+	+		6	-71.55	159.11	0.09	0.37
6.28	+	+			6	-72.55	161.10	2.08	0.14

Table 4: The 95% confidence set of models investigating differences in ***diel peak period in hours in activity behavior during three breeding stages*** *(nesting, gosling, molt periods) within a cumulative Akaike weight of ≥ 0.95 from the top model. The '+' indicates which variables are present in the different models, delta gives the difference in AICc between the models, and weight represents the relative probability that a certain model is best compared with a set of models, based on the data and taking model complexity into account.*

**Intercept**	**sex**	**breeding stage**	**year**	**sex : breeding stage**	**df**	**logLik**	**AICc**	**delta**	**weight**
23.61					3	-74.04	154.89	0.00	0.49
23.82			+		4	-73.72	156.82	1.93	0.19
23.35	+				4	-73.82	157.02	2.13	0.17
23.55	+		+		5	-73.48	159.10	4.21	0.06

Rhythmicity in corticosterone metabolites

Table 5: The 95% confidence set of linear fixed-effects models investigating rhythmicity in log-transformed ***corticosterone metabolite (CORTm) concentrations*** *over the course of the day within a cumulative Akaike weight of ≥ 0.95 from the top model. The '+' indicates which variables are present in the different models, delta gives the difference in AICc between the models, and weight represents the relative probability that a certain model is best compared with a set of models, based on the data and taking model complexity into account.*

**Intercept**	**cosine**	**sine**	**sex**	**year**	**day of the year**	**df**	**logLik**	**AICc**	**Delta**	**weight**
4.99	0.10	0.06		+	-0.007	7	-708.88	1431.93	0.00	0.31
4.89	0.11			+	-0.007	6	-710.02	1432.16	0.23	0.28
5.03	0.10	0.06	+	+	-0.007	8	-708.51	1433.23	1.30	0.16
4.93	0.10		+	+	-0.007	7	-709.66	1433.50	1.56	0.14
4.85		0.06		+	-0.007	6	-712.57	1437.26	5.33	0.02
4.75				+	-0.006	5	-713.87	1437.82	5.89	0.02
4.90		0.06	+	+	-0.007	7	-712.16	1438.48	6.55	0.01
4.35	0.10				-0.005	5	-714.46	1439.01	7.07	0.01

Table 6: The 95% confidence set of linear fixed-effects models investigating rhythmicity in a ***subset of log-transformed corticosterone metabolite concentrations*** *over the course of the day, i.e. excluding samples from one specific day, where CORTm values were unusually high, i.e. concentrations of >200 ng CORTm/g droppings (n=7), within a cumulative Akaike weight of ≥ 0.95 from the top model. The '+' indicates which variables are present in the different models, delta gives the difference in AICc between the models, and weight represents the relative probability that a certain model is best compared with a set of models, based on the data and taking model complexity into account.*

Intercept	cosine	sine	sex	year	day of the year	df	logLik	AICc	delta	weight
3.51	0.07			+		5	-658.32	1326.74	0.00	0.18
4.05	0.08			+	-0.003	6	-657.50	1327.13	0.39	0.15
3.55	0.07		+	+		6	-657.83	1327.78	1.05	0.10
4.09	0.07		+	+	-0.003	7	-657.00	1328.17	1.43	0.09
3.49				+		4	-660.25	1328.57	1.83	0.07
3.51	0.07	0.01		+		6	-658.25	1328.63	1.89	0.07
4.09	0.07	0.02		+	-0.003	7	-657.33	1328.83	2.09	0.06
3.54			+	+		5	-659.71	1329.52	2.78	0.04
3.93				+	-0.002	5	-659.71	1329.52	2.78	0.04
3.55	0.07	0.01	+	+		7	-657.75	1329.67	2.94	0.04
4.13397	0.07	0.02	+	+	-0.003	8	-656.82	1329.86	3.12	0.04
3.49		0.02		+		5	-660.14	1330.37	3.64	0.03
3.97			+	+	-0.002	6	-659.16	1330.46	3.72	0.03

Table 7: Intercept and coefficient estimates from the full model investigating rhythmicity in a ***subset of log-transformed corticosterone metabolite concentrations****, i.e. excluding samples with concentrations of >200 ng CORTm/g droppings with corresponding 85% CIs, and if the variable was selected in the top AIC model.*

Variable	estimate	SE	Lower	upper	AIC top
Intercept	4.13	0.43	3.52	4.75	
cosine	0.073	0.04	0.02	0.13	Yes
sine	0.02	0.04	-0.03	0.07	No
sex - male	-0.09	0.09	-0.22	0.04	No
year - 2021	-0.22	0.06	-0.31	-0.13	Yes
year day	-0.003	0.002	-0.006	0.0001	No

Supplement: Figures

Seasonal rhythmicity in activity

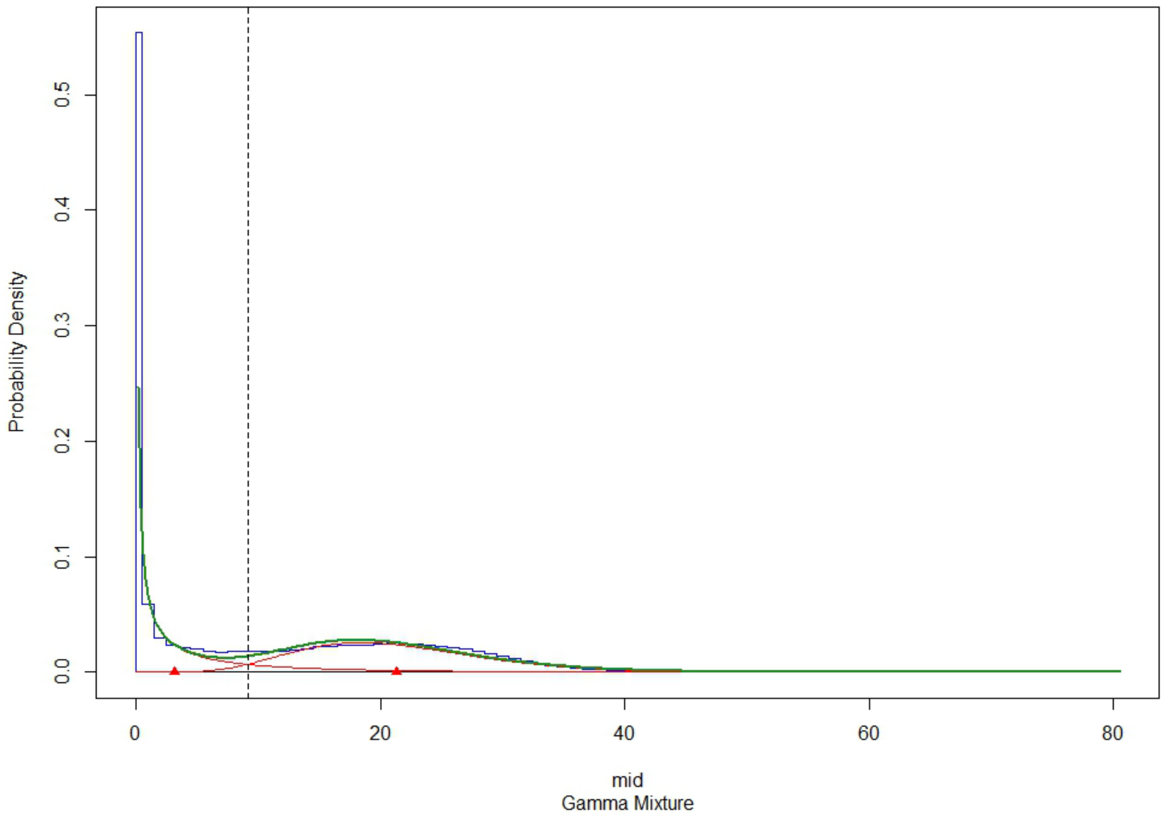

Figure 1: Probability density histogram for ***vectoral dynamic body acceleration (VeDBA).*** *The blue line shows the probability density histogram, the green lines show the corresponding probability density functions, and the red lines depict the fitted gamma distributions. The red triangles give the mid points of the gamma distributions for inactive and active behavior. The dashed vertical line is the intersection point between the distributions and as such gives the threshold (9.15) used to distinguish VeDBA values indicating active and inactive behavior.*

Incubation behavior

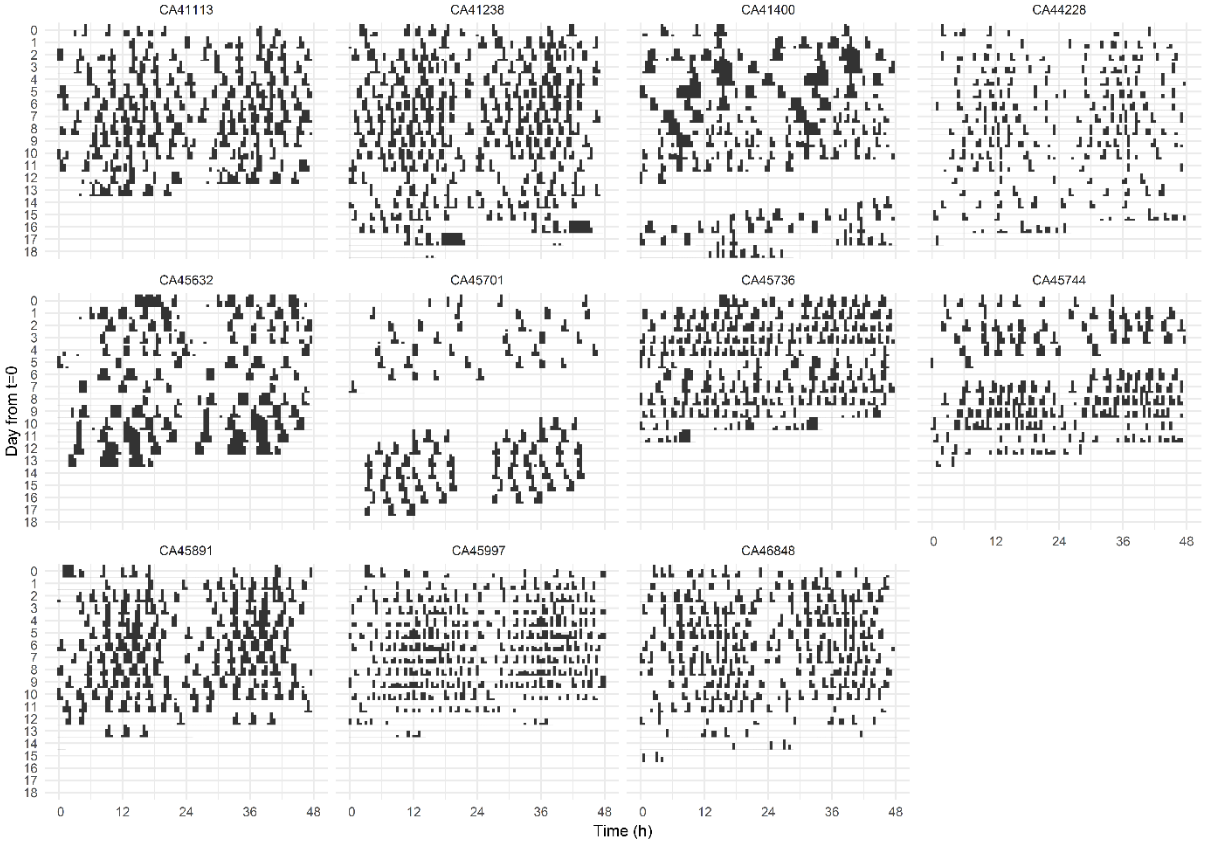

Figure 2: Double-plotted actograms of ***incubation recesses*** *for all individual females. Day t = 0 is measured from when the camera was set up near the nest. Most cameras were placed during the early morning of 2021-06-18, except for geese with the IDs CA45632 (2021-06-25 14:52:13), CA45701 (2021-06-09 15:17:49), and CA45736 (2021-06-25 15:26:05). All but one female CA45701 (second panel middle row), whose nest failed for unknown reasons, had successful nests. For three females, data are incomplete due to camera malfunction: for CA41400 data of two days are missing, for CA45701 data of three days are missing, and for CA45744 data of one day are missing.*

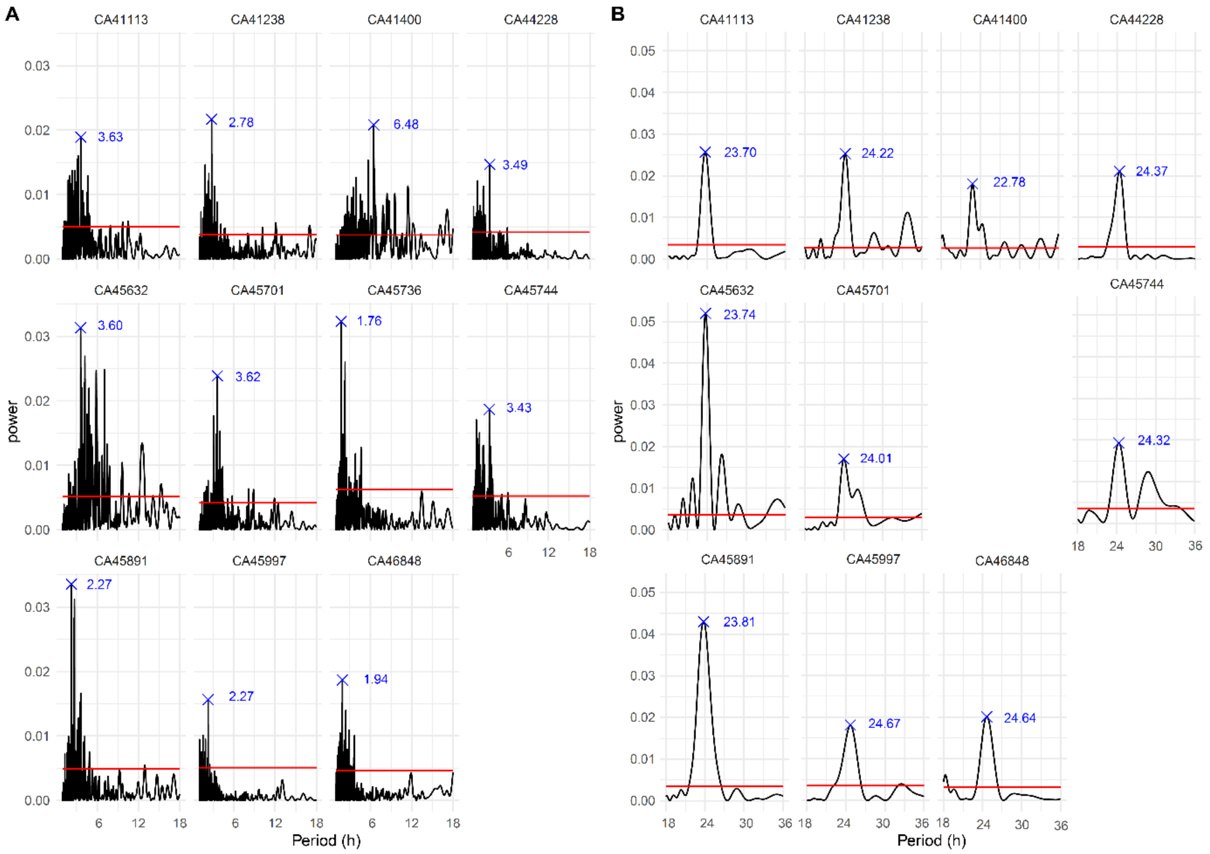

Figure 3: Lomb-Scargle periodograms of ***incubation recesses*** *for all individual geese. The analysis identified periodicity between A) 1 and 18 hours to focus on ultradian rhythmicity and B) between 18 and 36 hours to focus on diel rhythmicity. Peaks above the significance threshold of P < 0.05 (red line) are shown in blue. Apart from CA45736, who only showed ultradian rhythmicity (third panel middle row), all geese showed both ultradian and diel rhythmicity. All geese had successful nests except for CA45701 whose nest failed because of unknown reasons.*

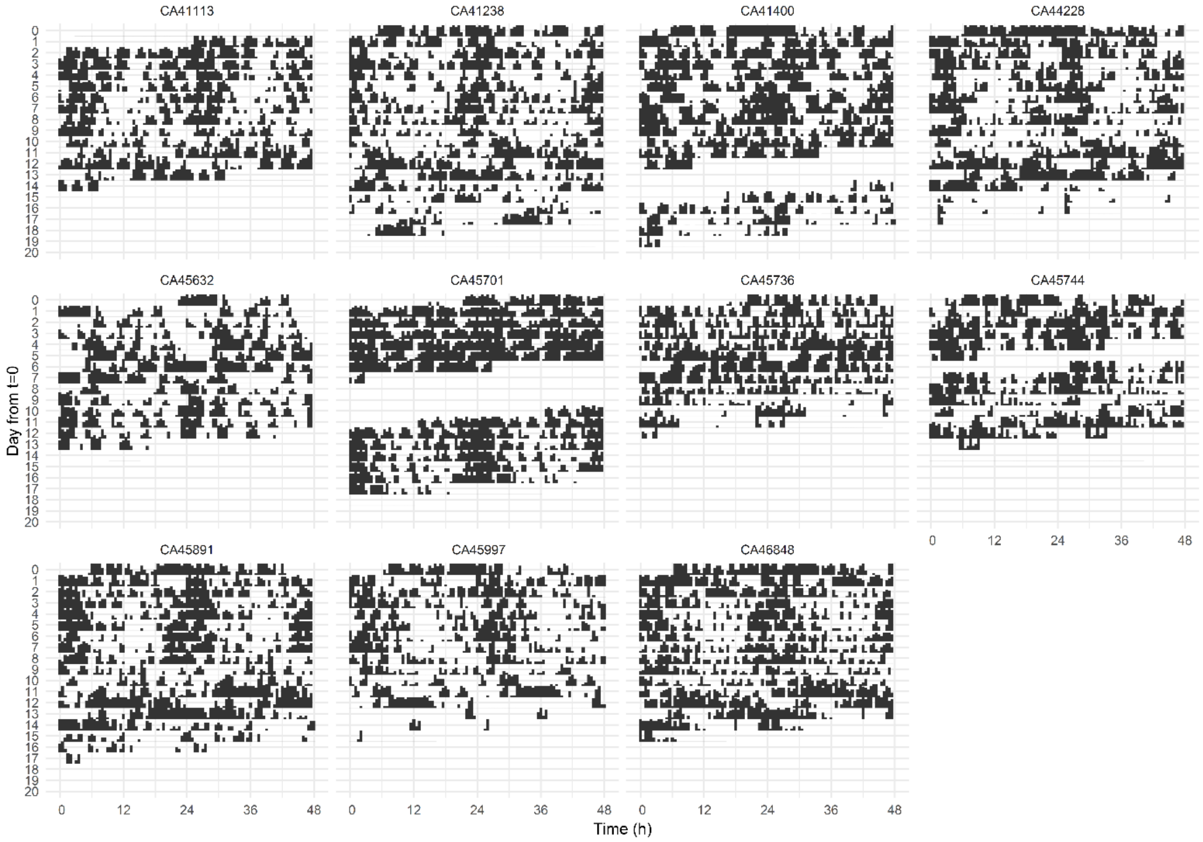

Figure 4: Double-plotted actograms of ***sleep posture*** *for all individual females. Day t = 0 is measured from when the camera was set up near the nest. All but one female CA45701 (second panel middle row), whose nest failed for unknown reasons, had successful nests. For three females, data are incomplete due to camera malfunction: for CA41400 data of two days are missing, for CA45701 data of three days are missing, and for CA45744 data of one day are missing.*

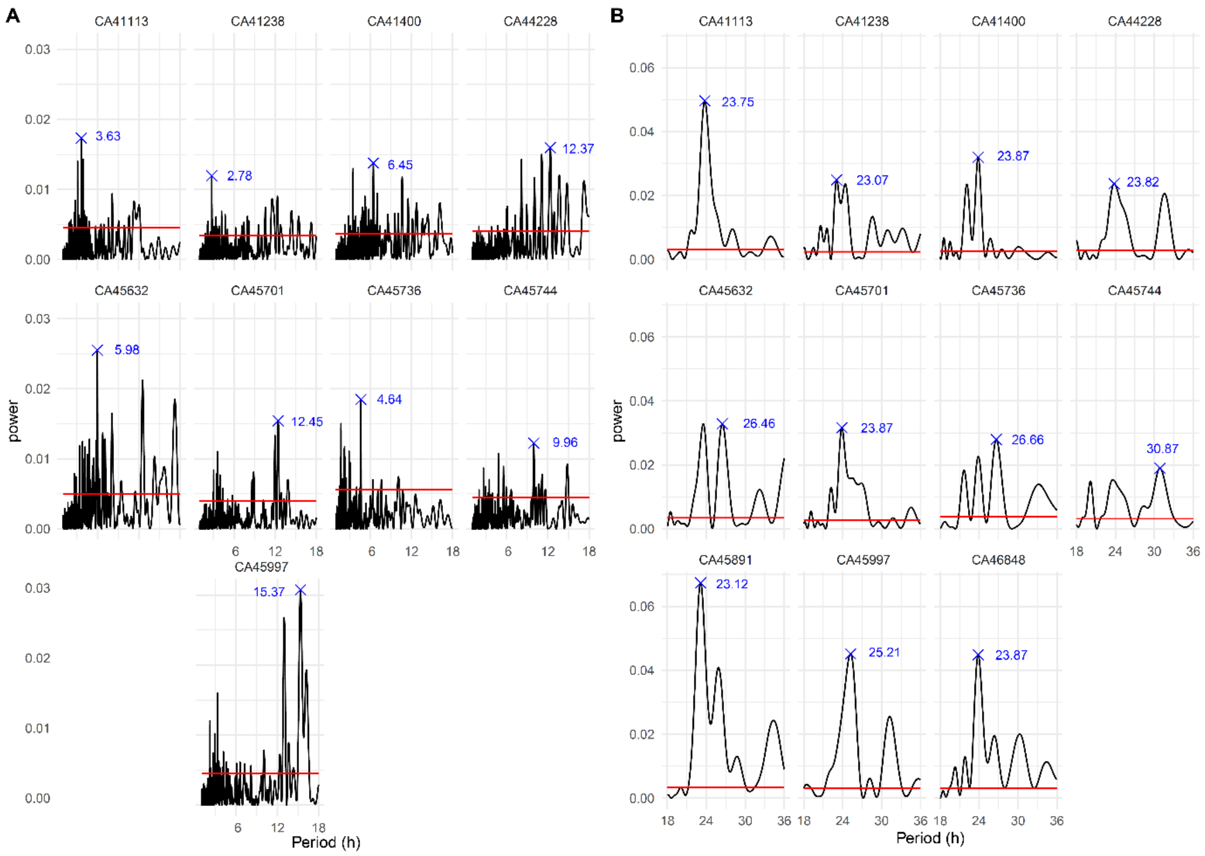

*Figure 5: Lomb-Scargle periodograms of **sleep posture** for all individual geese. The analysis identified periodicity between (A) 1 and 18 hours to focus on ultradian rhythmicity and (B) between 18 and 36 hours to focus on diel rhythmicity. Peaks above the significance threshold of P < 0.05 (red line) are shown in blue. Apart from CA45891and CA46848, who only showed diel rhythmicity (first and third panel bottom row), geese showed both ultradian and diel rhythmicity. All geese had successful nests except for CA45701 whose nest failed because of unknown reasons.*

Seasonal rhythmicity in activity: Actograms

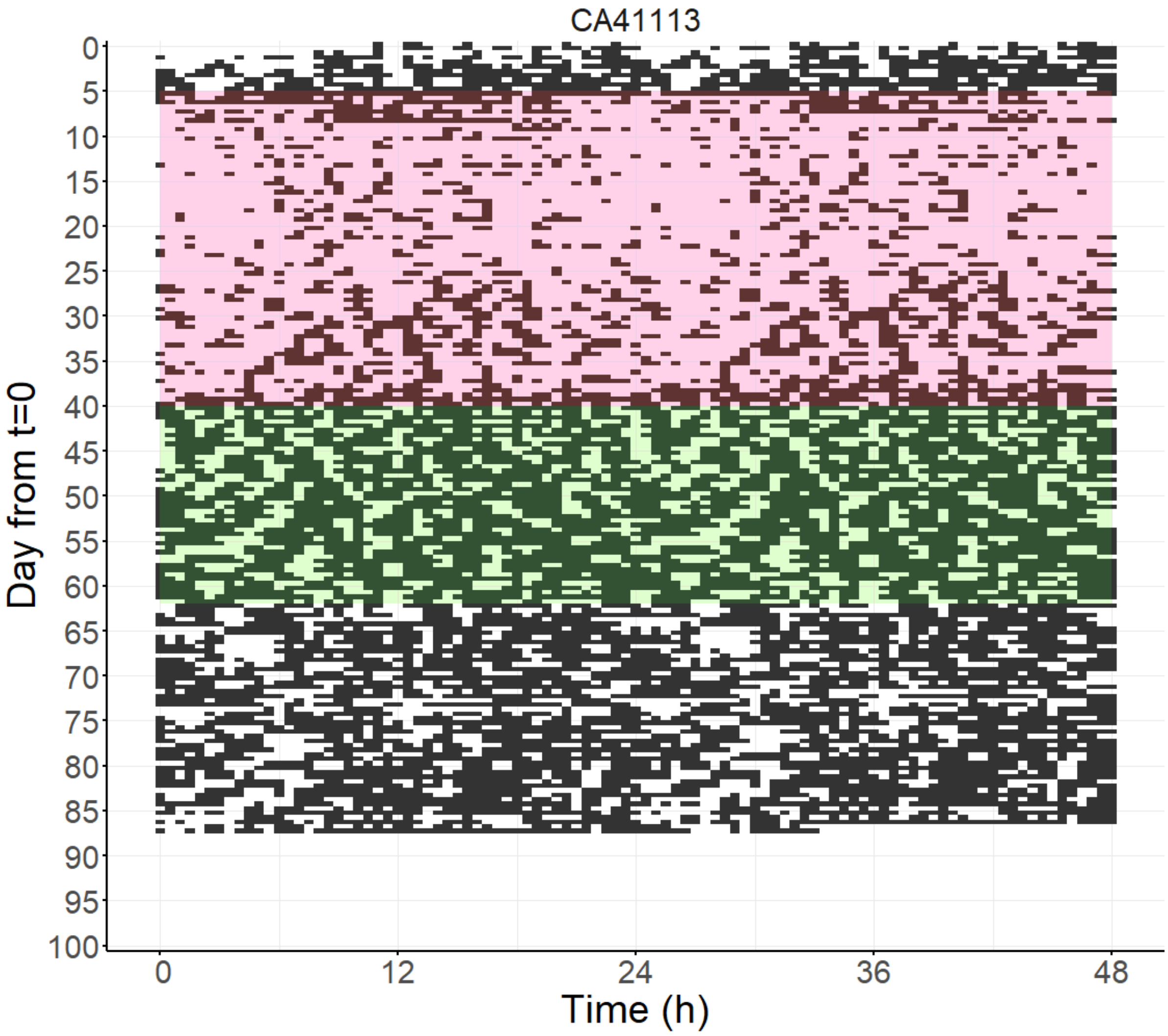

Figure 6: ***Rhythmicity in activity across the 2021 season*** *in female goose CA41113. Day t = 0 was 2021-05-24 10:31:11. Double-plotted actogram with the nesting phase in pink and the gosling phase in green. The nesting phase was based on accelerometer and GPS data from neckband transmitters and the gosling phase was based on observations. This female was not observed during molt. In double-plotted actograms, the x-axis displays two consecutive days, and these consecutive days are also shown from top to bottom on the y-axis. Activity is shown in black, while transparency indicates inactivity.*

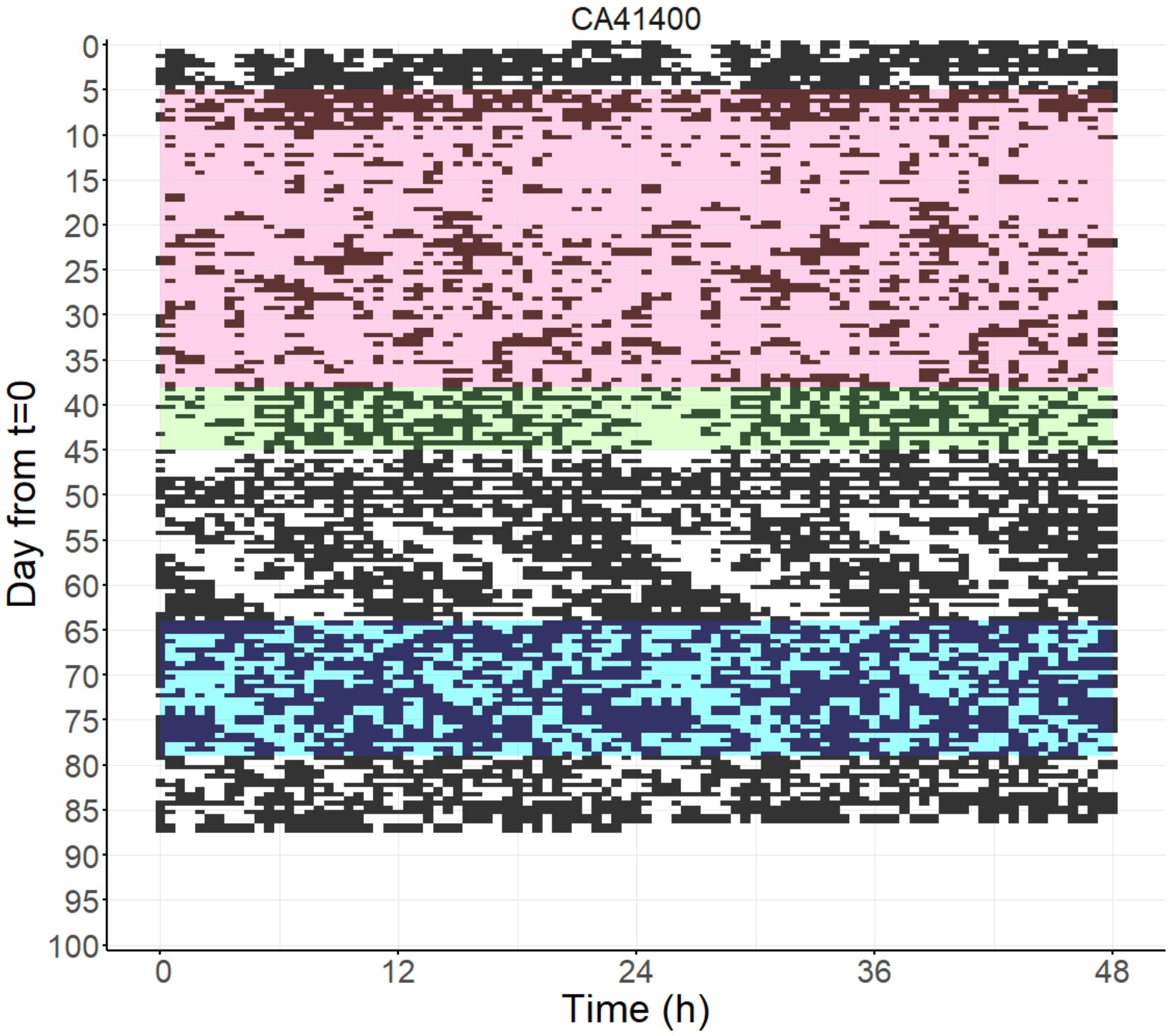

Figure 7: ***Rhythmicity in activity across the 2021 season*** *in female goose CA41400. Day t = 0 was 2021-05-30 21:41:23. Double-plotted actogram with the nesting phase in pink, the gosling phase in green and the molting phase in blue. The nesting phase was based on accelerometer and GPS data from neckband transmitters, the gosling and molt phases were based on observations. In double-plotted actograms, the x-axis displays two consecutive days, and these consecutive days are also shown from top to bottom on the y-axis. Activity is shown in black, while transparency indicates inactivity.*

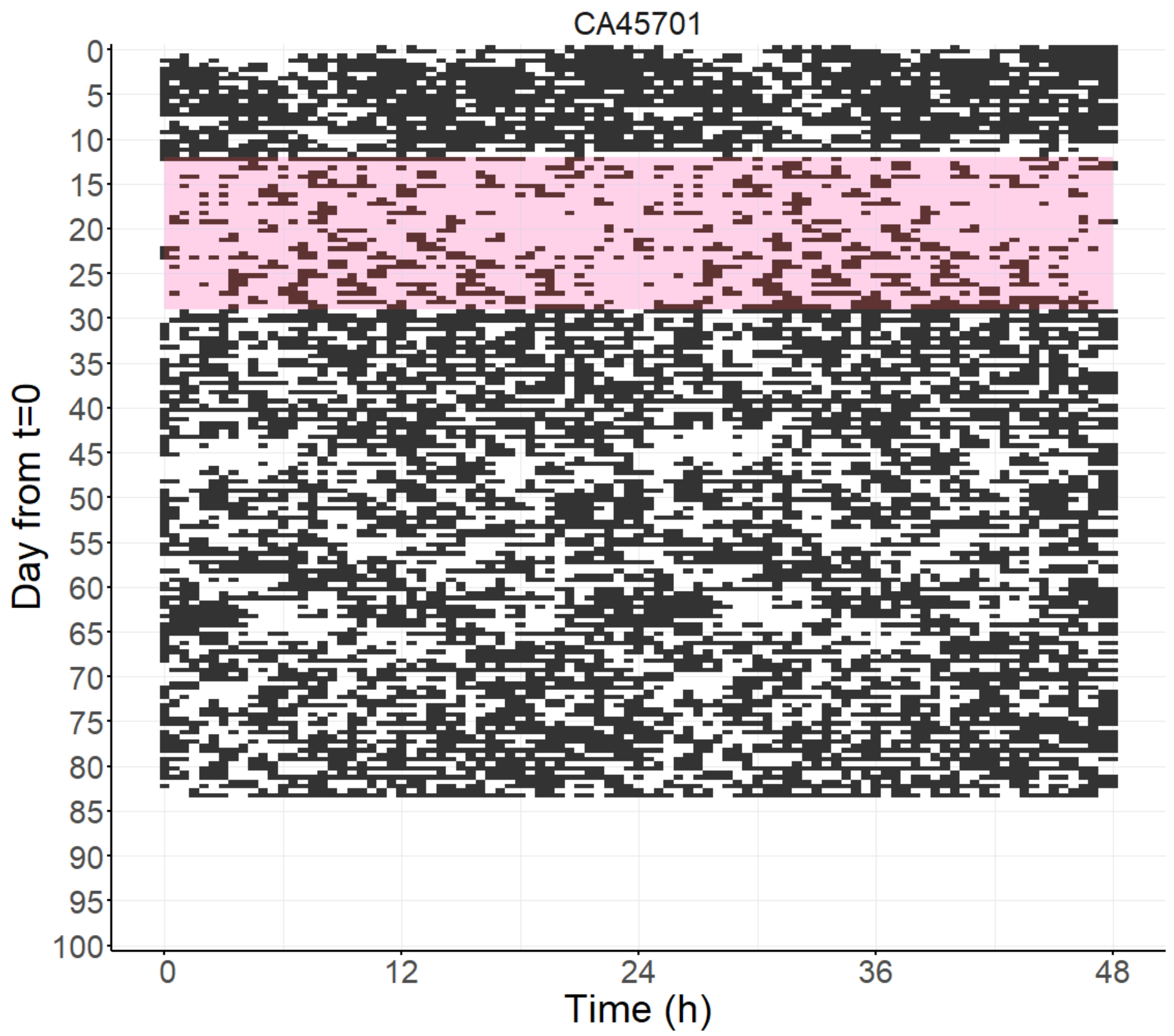

Figure 8: ***Rhythmicity in activity across the 2021 season*** *in female goose CA45701. Day t = 0 was 2021-05-28 10:45:38. Double-plotted actogram with the nesting phase in pink. The nesting phase was based on accelerometer and GPS data from neckband transmitters. In double-plotted actograms, the x-axis displays two consecutive days, and these consecutive days are also shown from top to bottom on the y-axis. Activity is shown in black, while transparency indicates inactivity.*

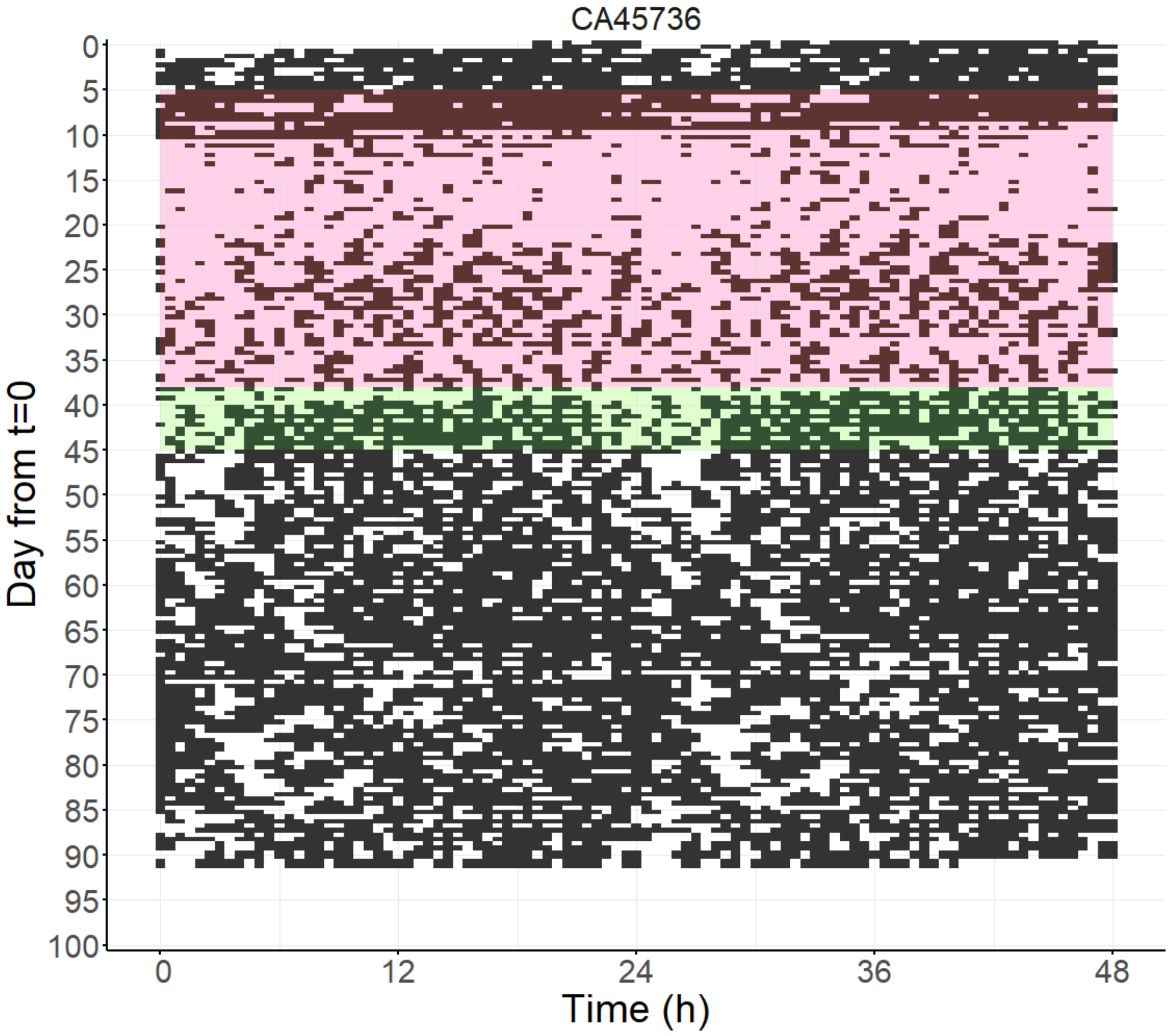

Figure 9: ***Rhythmicity in activity across the 2021 season*** *in female goose CA45736. Day t = 0 was 2021-05-27 19:31:11. Double-plotted actogram with the nesting phase in pink and gosling phase in green. The nesting phase was based on accelerometer and GPS data from neckband transmitters, while the gosling phase was based on observations. In double-plotted actograms, the x-axis displays two consecutive days, and these consecutive days are also shown from top to bottom on the y-axis. Activity is shown in black, while transparency indicates inactivity.*

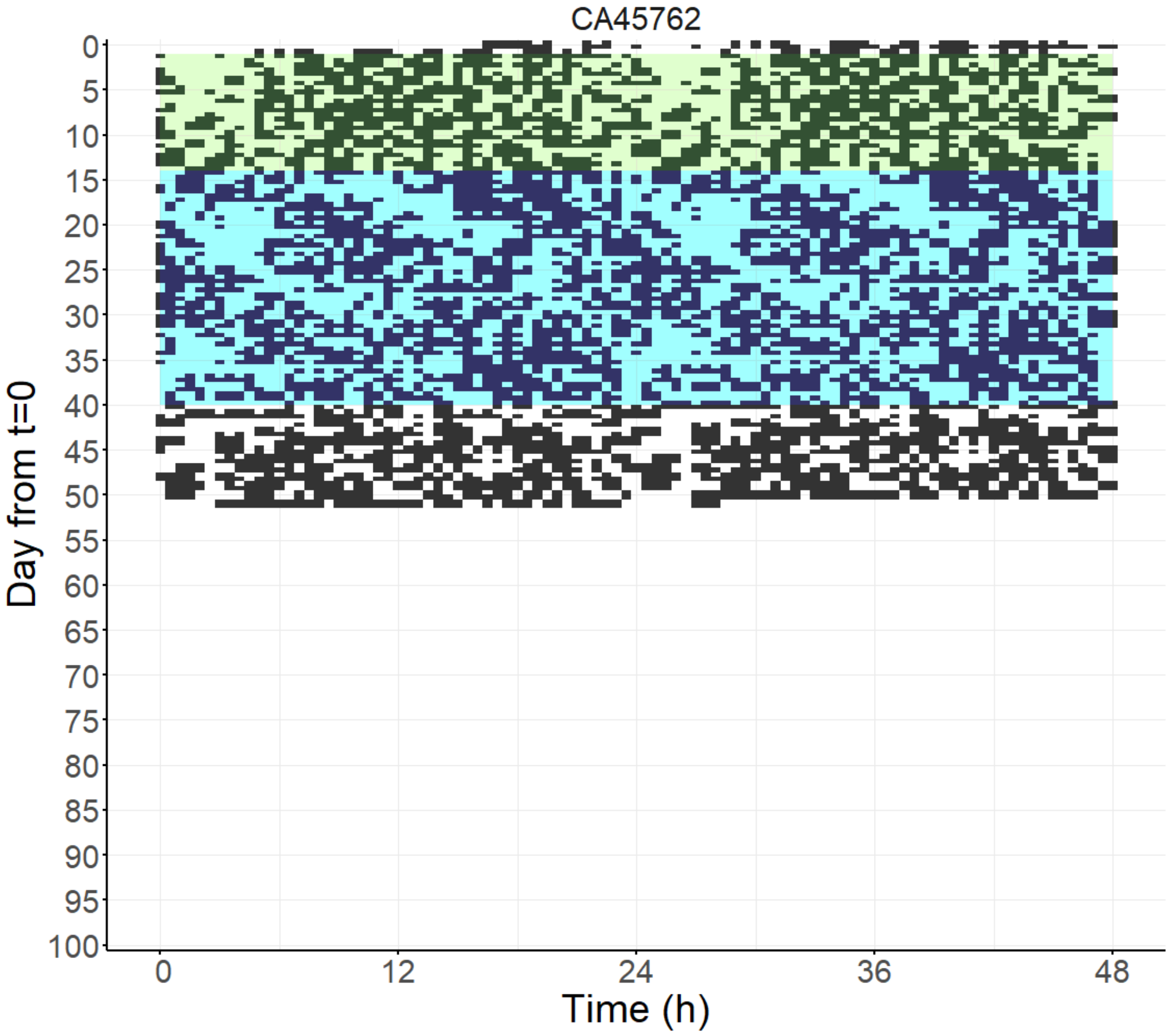

Figure 10: ***Rhythmicity in activity across the 2021 season*** *in female goose CA45762. Day t = 0 was 2021-07-05 17:19:58. Double-plotted actogram with the gosling phase in green and the molt phase in blue. The gosling and molt phases were based on observations. In double-plotted actograms, the x-axis displays two consecutive days, and these consecutive days are also shown from top to bottom on the y-axis. Activity is shown in black, while transparency indicates inactivity.*

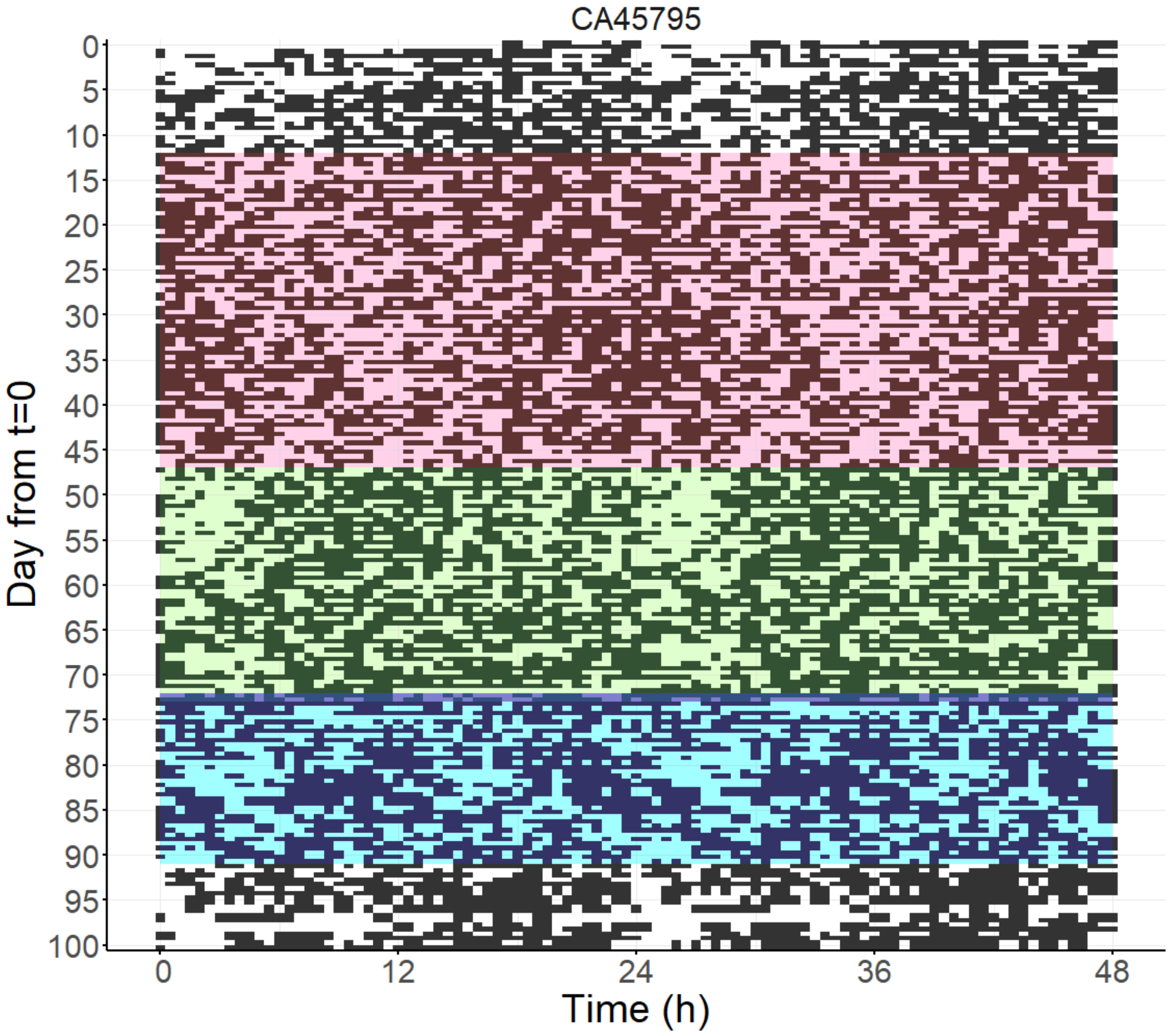

Figure 11: ***Rhythmicity in activity across the 2021 season*** *in male goose CA45795. Day t = 0 was 2021-05-21 18:16:46. Double-plotted actogram with the nesting phase in pink, the gosling phase in green, the gosling & molt phase in dark blue and the molting phase in light blue. The nesting phase was based on accelerometer and GPS data from neckband transmitters, the gosling and molt phases were based on observations. In double-plotted actograms, the x-axis displays two consecutive days, and these consecutive days are also shown from top to bottom on the y-axis. Activity is shown in black, while transparency indicates inactivity.*

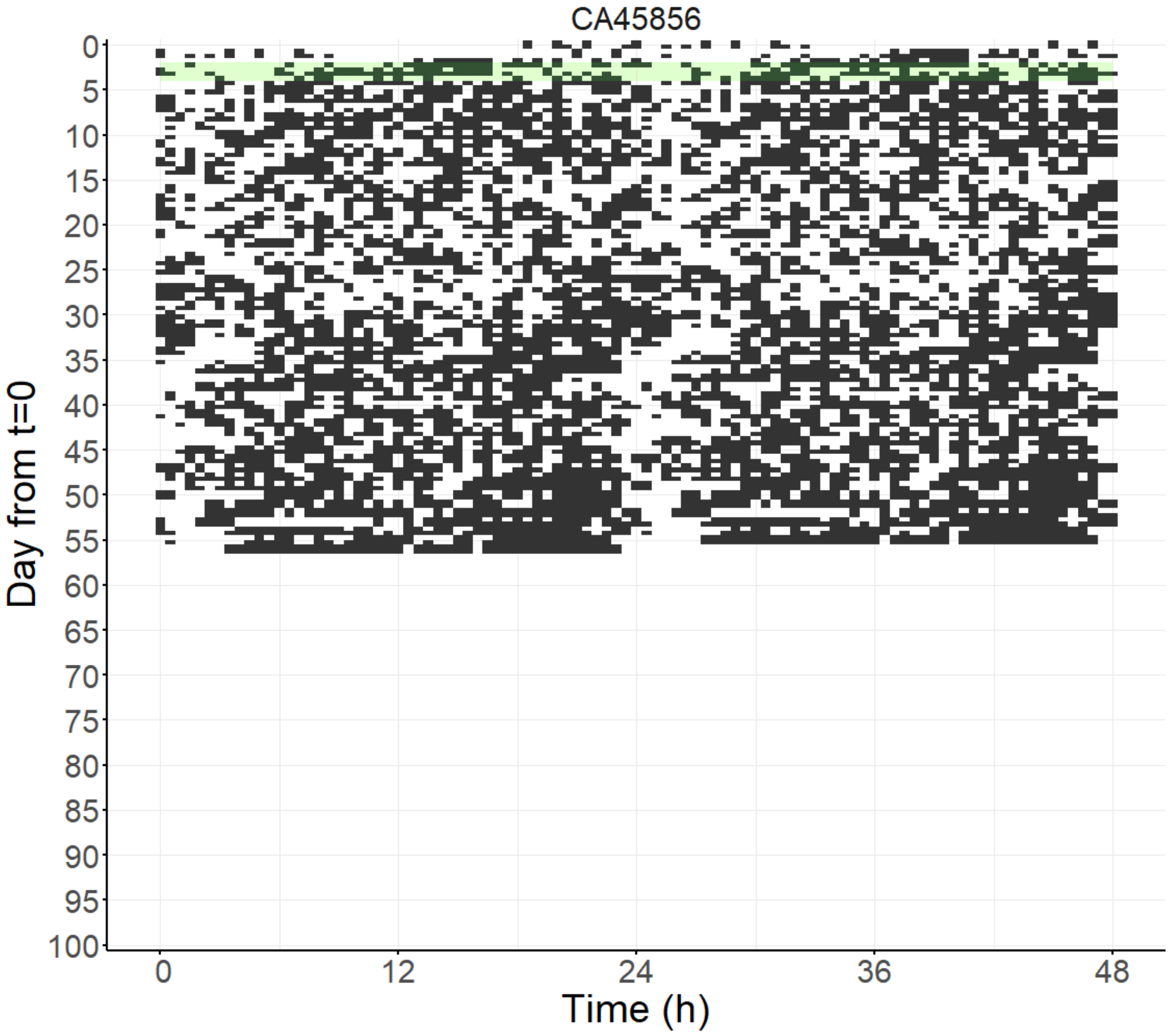

Figure 12: ***Rhythmicity in activity across the 2021 season*** *in male goose CA45856. Day t = 0 was 2021-07-05 17:15:17. Double-plotted actogram with the gosling phase in green. The gosling phase was based on observations. In double-plotted actograms, the x-axis displays two consecutive days, and these consecutive days are also shown from top to bottom on the y-axis. Activity is shown in black, while transparency indicates inactivity.*

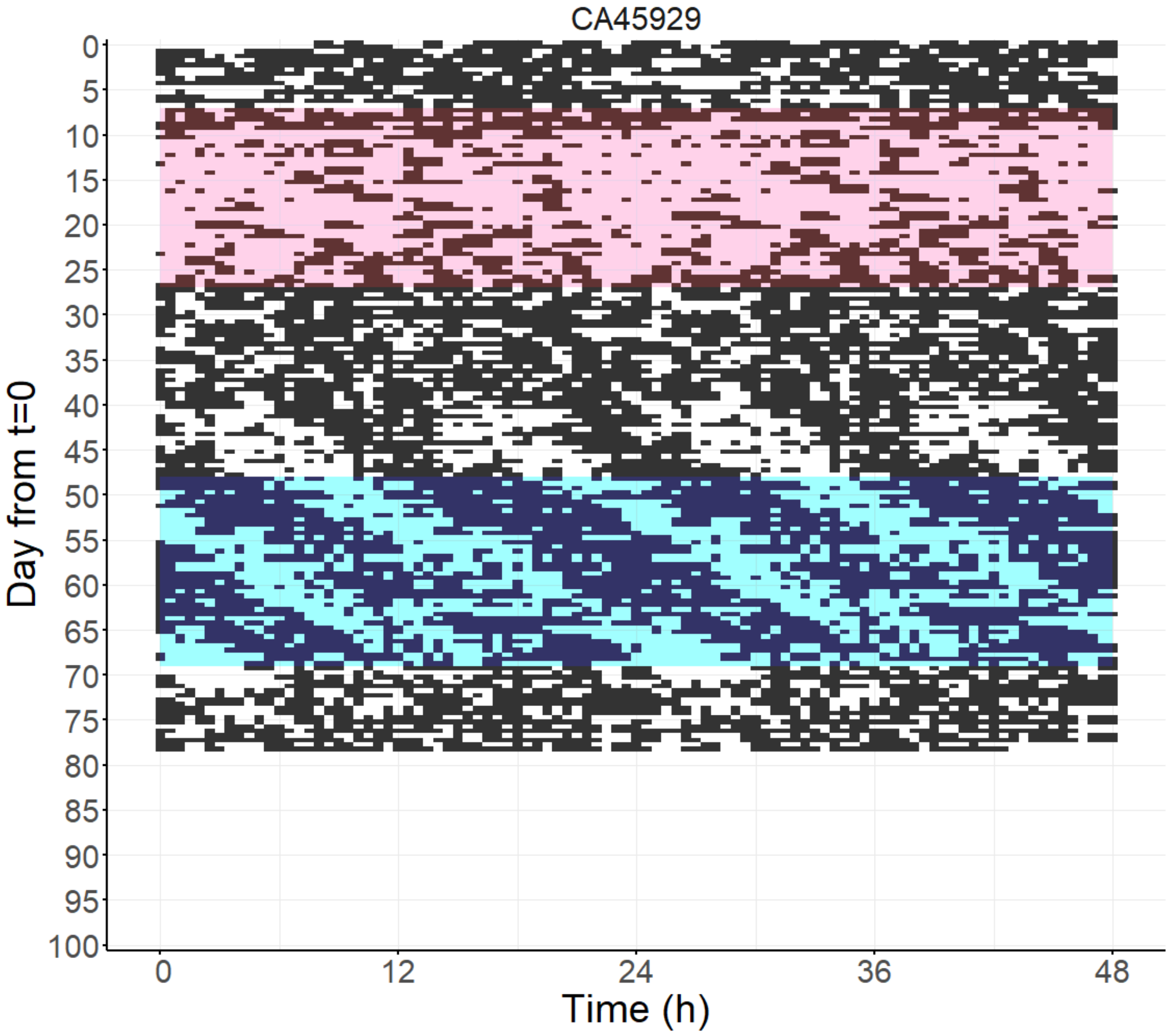

Figure 13: ***Rhythmicity in activity across the 2021 season*** *in female goose CA45929. Day t = 0 was 2021-05-31 08:48:49. Double-plotted actogram with the nesting phase in pink and the molt phase in light blue. The nesting phase was based on accelerometer and GPS data from neckband transmitters, molt phase was based on observations. In double-plotted actograms, the x-axis displays two consecutive days, and these consecutive days are also shown from top to bottom on the y-axis. Activity is shown in black, while transparency indicates inactivity.*

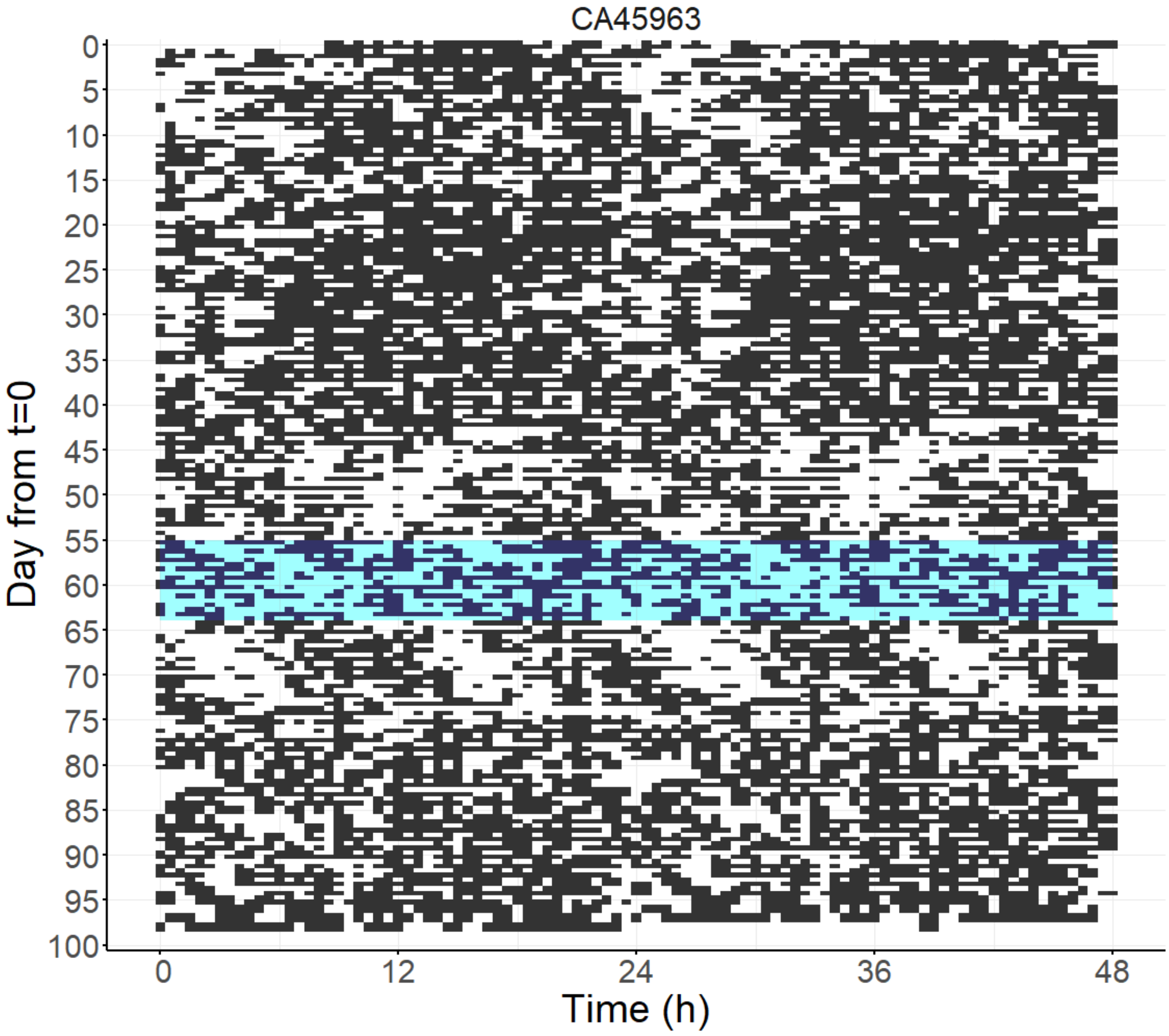

Figure 14: ***Rhythmicity in activity across the 2021 season*** *in female goose CA45963. Day t = 0 was 2021-05-19 09:20:48. Double-plotted actogram with the molt phase in light blue. The molt phase was based on observations. In double-plotted actograms, the x-axis displays two consecutive days, and these consecutive days are also shown from top to bottom on the y-axis. Activity is shown in black, while transparency indicates inactivity.*

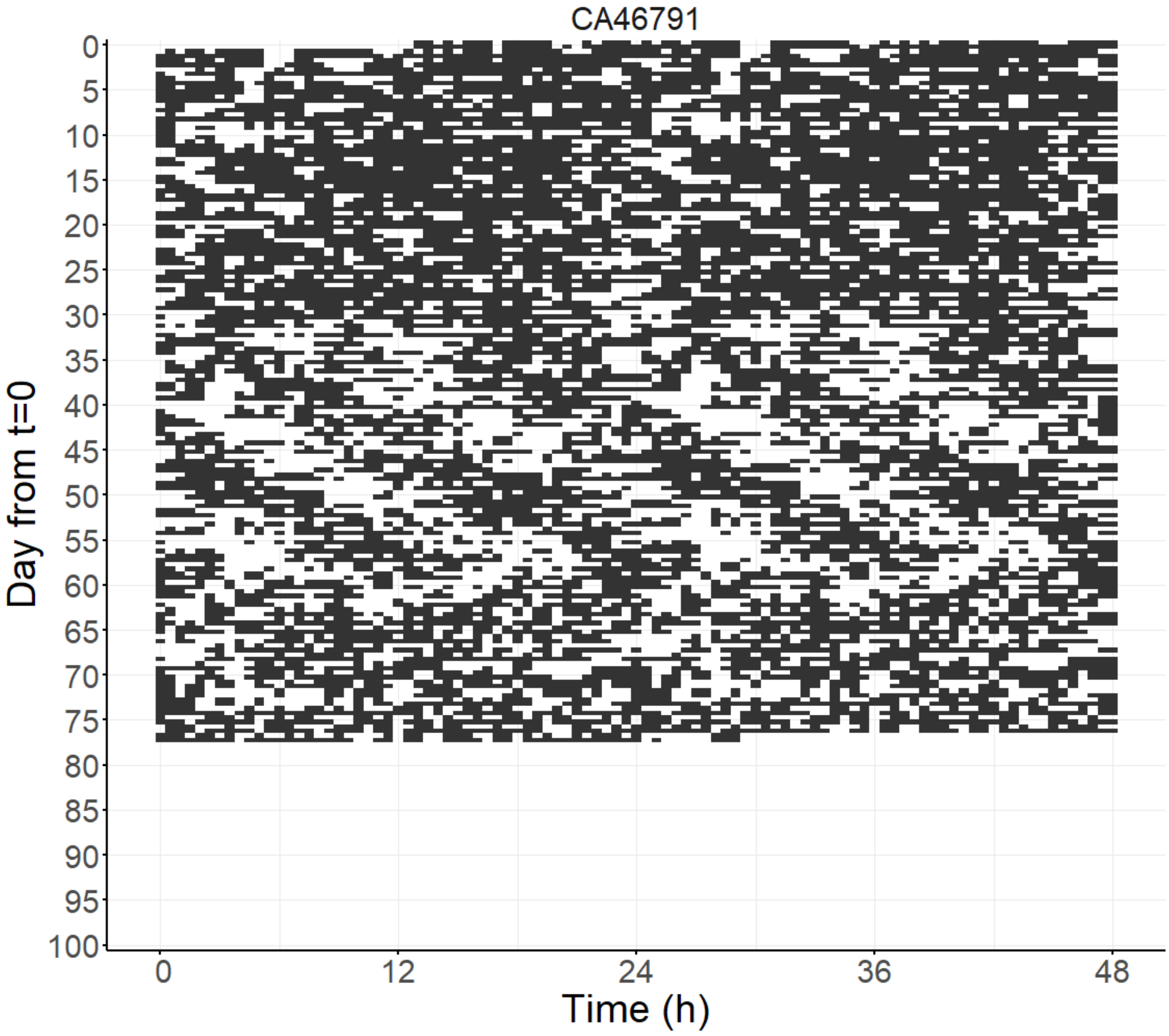

Figure 15: ***Rhythmicity in activity across the 2021 season*** *in male goose CA46791. Day t = 0 was 2021-06-02 13:58:22. Double-plotted actogram with no observations on the nesting, gosling or molt phase. In double-plotted actograms, the x-axis displays two consecutive days, and these consecutive days are also shown from top to bottom on the y-axis. Activity is shown in black, while transparency indicates inactivity.*

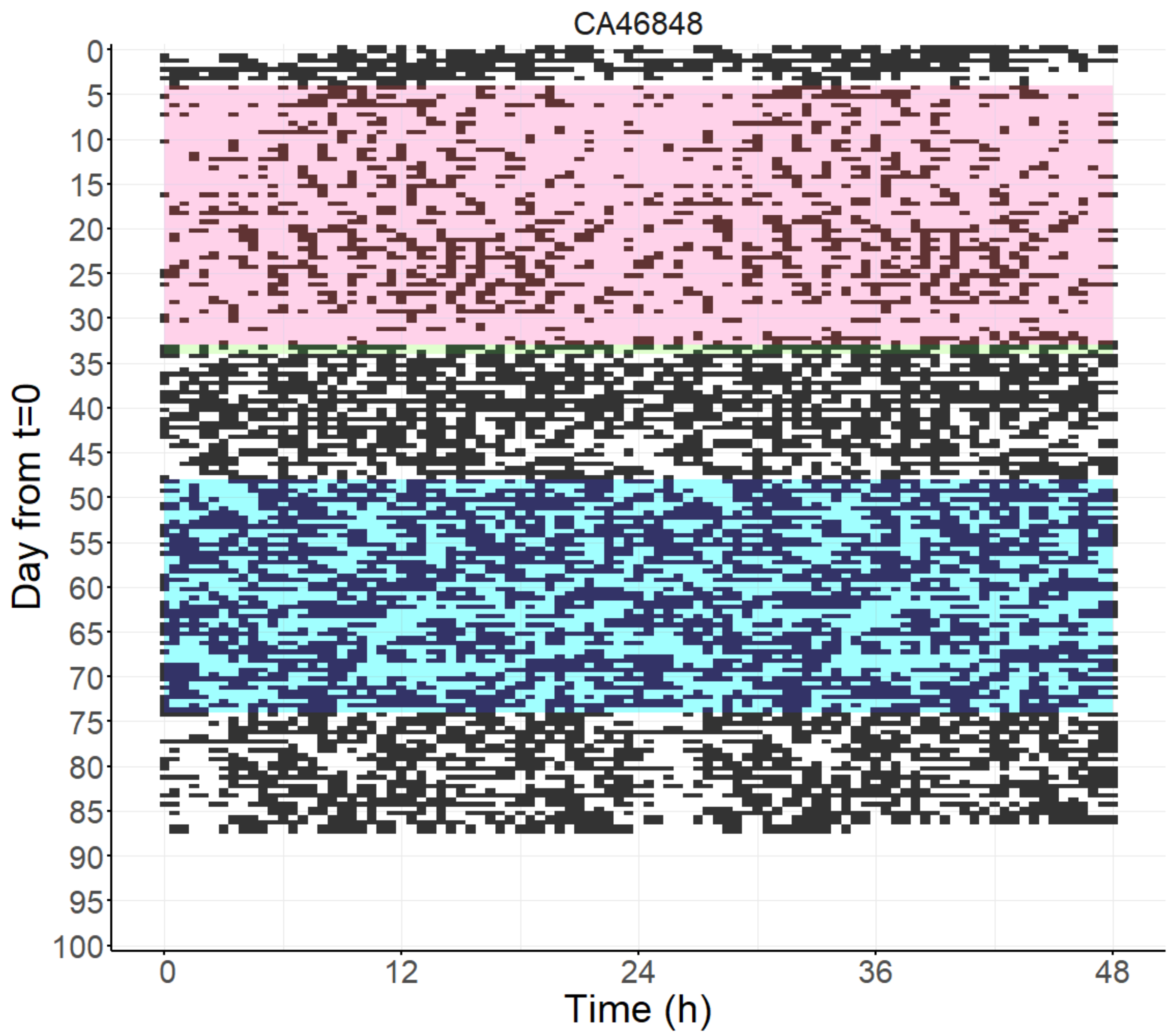

Figure 16: ***Rhythmicity in activity across the 2021 season*** *in female goose CA46848. Day t = 0 was 2021-05-31 06:50:13. Double-plotted actogram with the nesting phase in pink, the gosling phase in green and the molt phase in light blue. The nesting phase was based on accelerometer and GPS data from neckband transmitters, while the gosling and molt phases were based on observations. In double-plotted actograms, the x-axis displays two consecutive days, and these consecutive days are also shown from top to bottom on the y-axis. Activity is shown in black, while transparency indicates inactivity.*

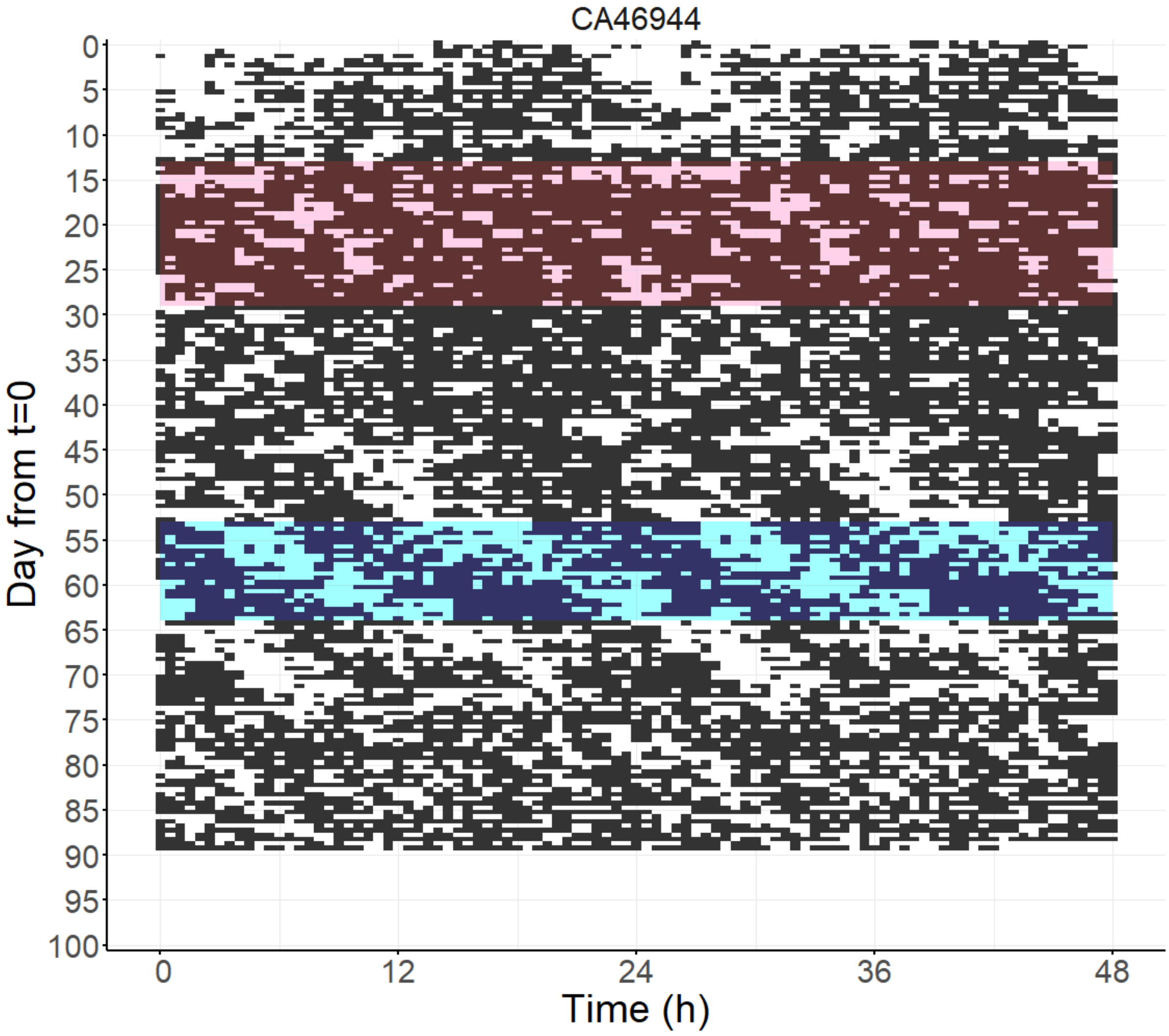

Figure 17: ***Rhythmicity in activity across the 2021 season*** *in male goose CA46944. Day t = 0 was 2021-05-21 13:39:18. Double-plotted actogram with the nesting phase in pink and the molt phase in light blue. The nesting phase was based on accelerometer and GPS data from neckband transmitters, while the molt phase was based on observations. In double-plotted actograms, the x-axis displays two consecutive days, and these consecutive days are also shown from top to bottom on the y-axis. Activity is shown in black, while transparency indicates inactivity.*

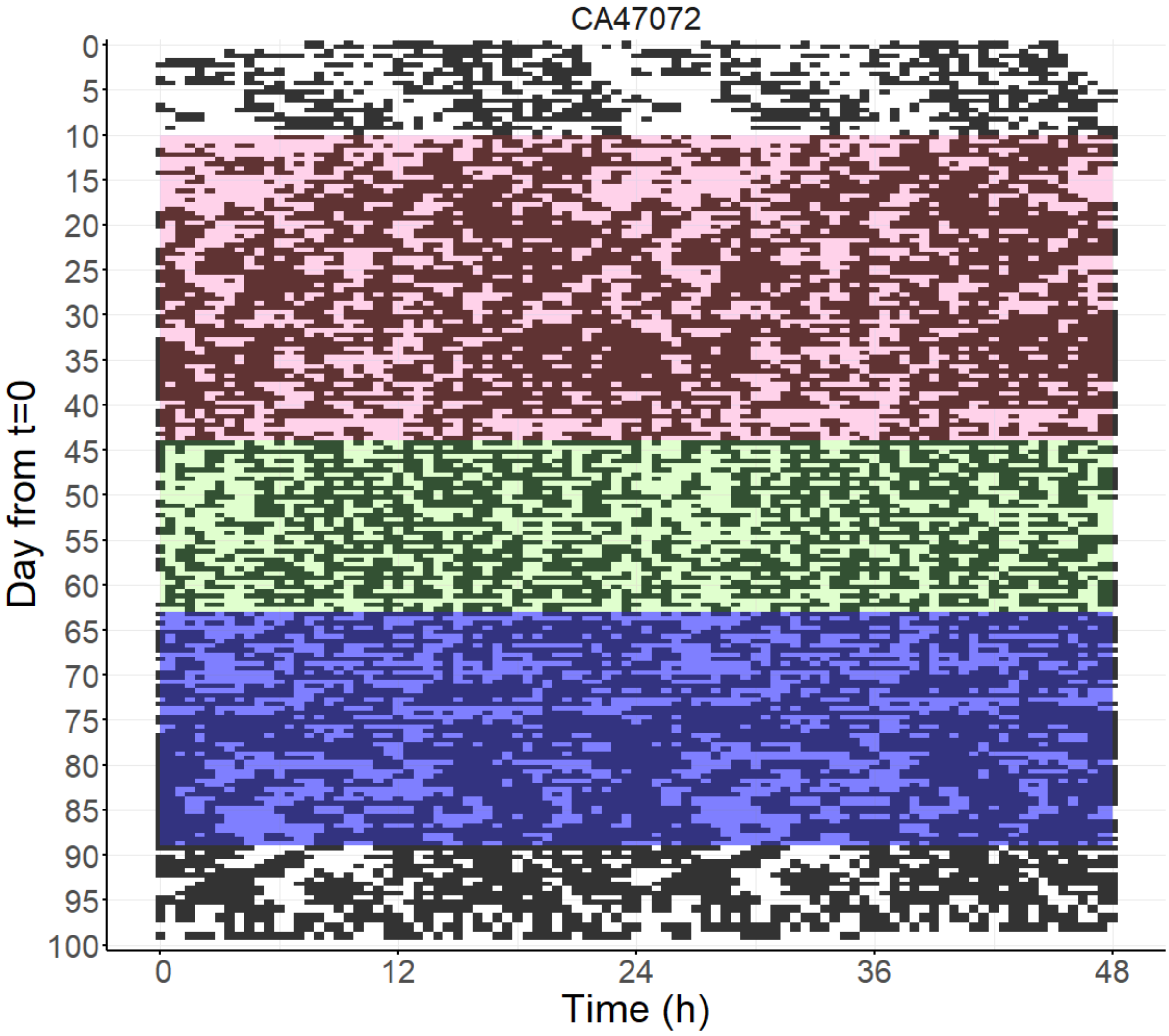

Figure 18: ***Rhythmicity in activity across the 2021 season*** *in male goose CA47072. Day t = 0 was 2021-05-19 08:07:07. Double-plotted actogram with the nesting phase in pink, the gosling phase in green and the gosling & molt phase in dark blue. The nesting phase was based on accelerometer and GPS data from neckband transmitters, while the gosling and molt phases were based on observations. In double-plotted actograms, the x-axis displays two consecutive days, and these consecutive days are also shown from top to bottom on the y-axis. Activity is shown in black, while transparency indicates inactivity.*

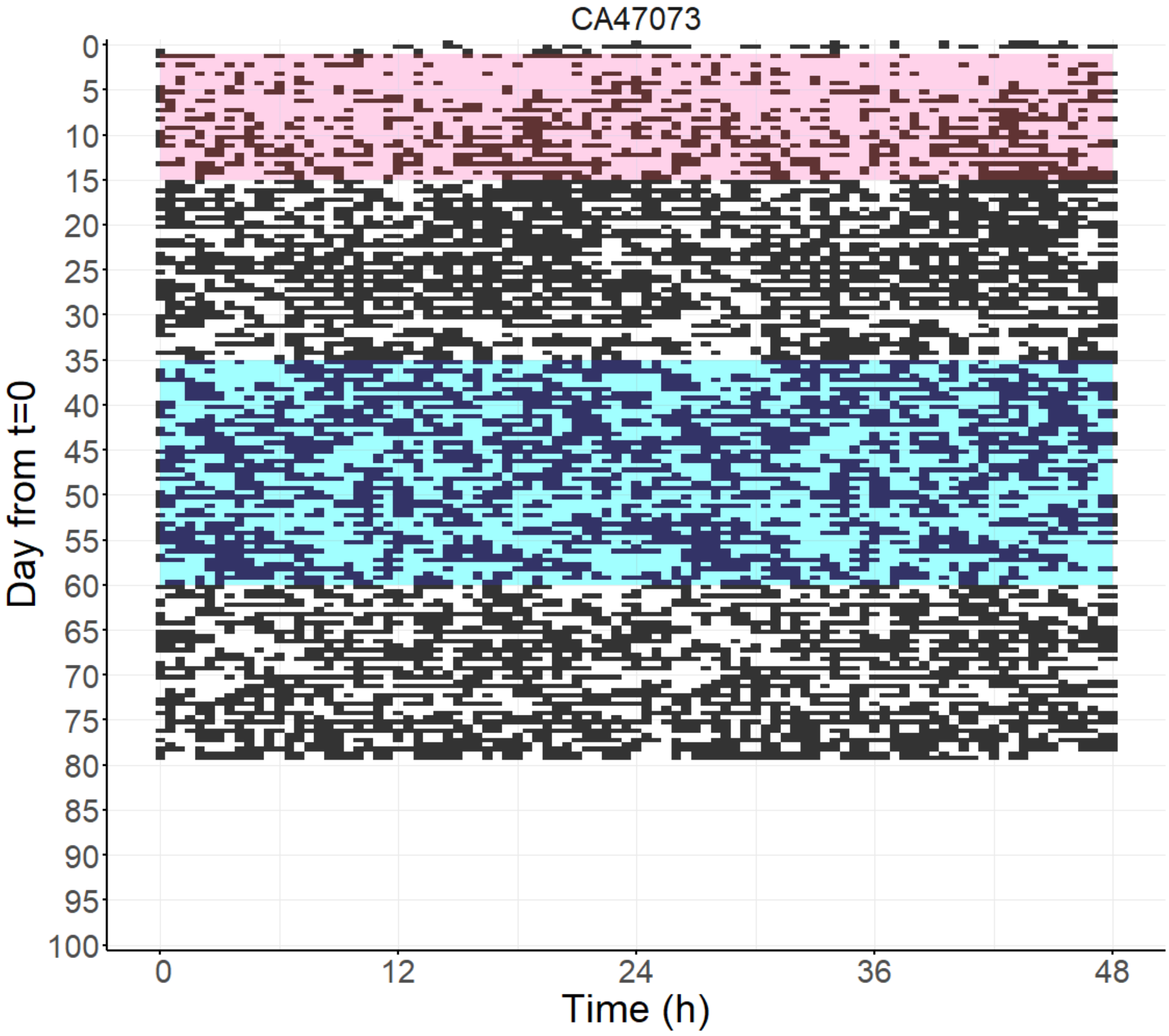

Figure 19: ***Rhythmicity in activity across the 2021 season*** *in male goose CA47073. Day t = 0 was 2021-06-08 12:40:59. Double-plotted actogram with the nesting phase in pink and the molt phase in light blue. The nesting phase was based on accelerometer and GPS data from neckband transmitters, while the molt phase was based on observations. In double-plotted actograms, the x-axis displays two consecutive days, and these consecutive days are also shown from top to bottom on the y-axis. Activity is shown in black, while transparency indicates inactivity.*

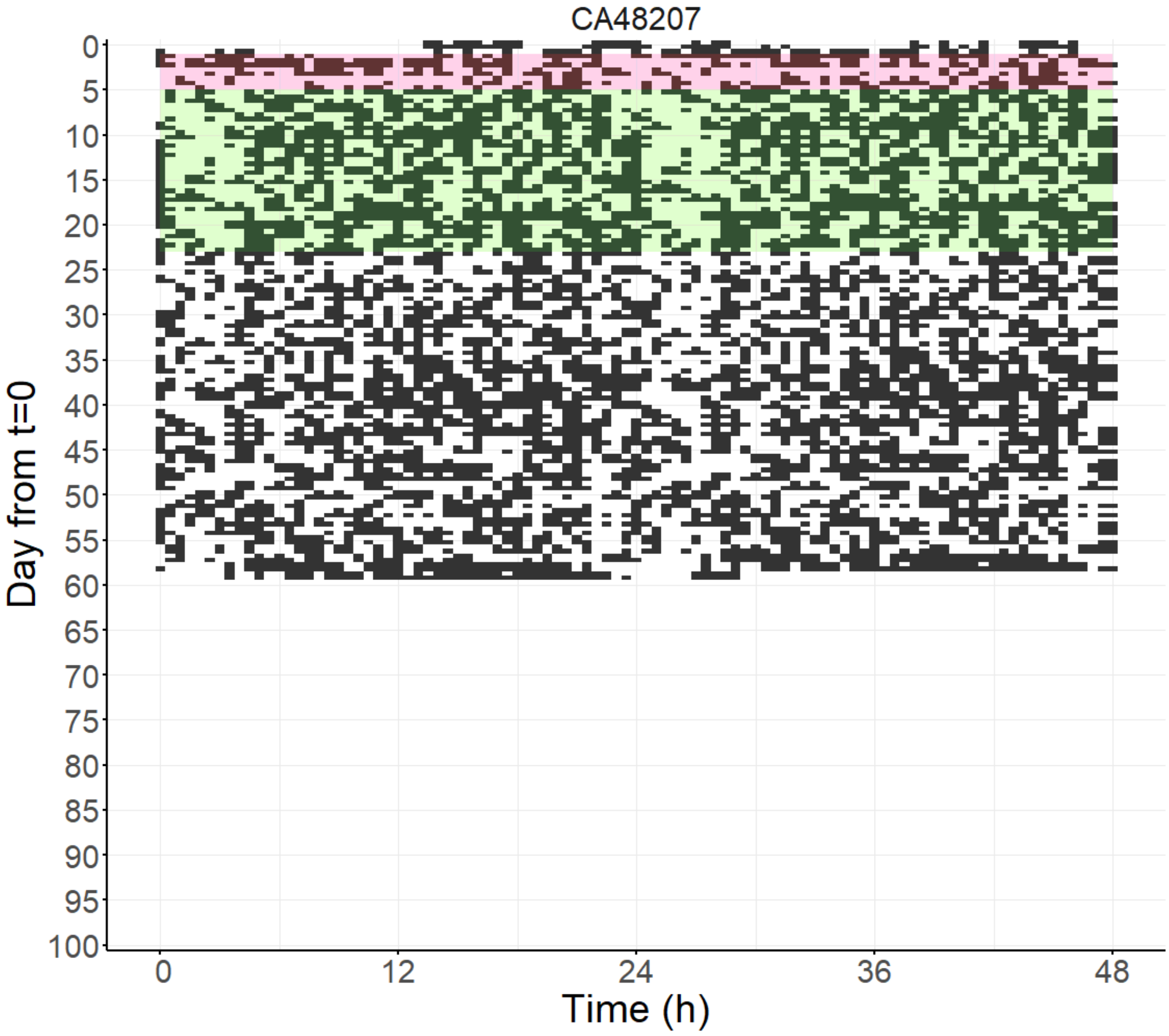

Figure 20: ***Rhythmicity in activity across the 2021 season*** *in male goose CA48207. Day t = 0 was 2021-06-29 12:12:09. Double-plotted actogram with the nesting phase in pink and the gosling phase in green. The nesting phase was based on accelerometer and GPS data from neckband transmitters, while the gosling phase was based on observations. In double-plotted actograms, the x-axis displays two consecutive days, and these consecutive days are also shown from top to bottom on the y-axis. Activity is shown in black, while transparency indicates inactivity.*

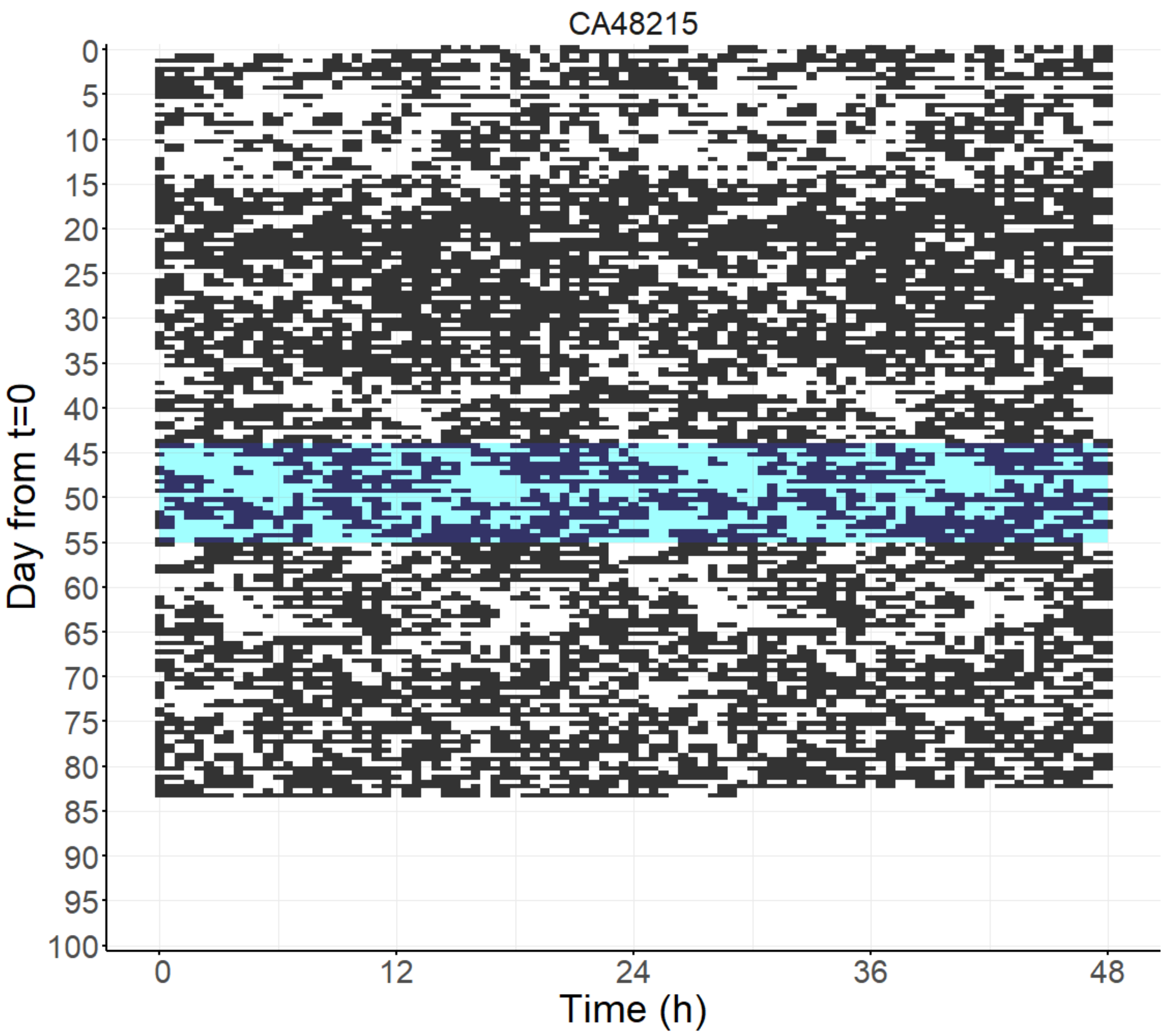

Figure 21: ***Rhythmicity in activity across the 2021 season*** *in male goose CA48215. Day t = 0 was 2021-05-27 09:40:35. Double-plotted actogram with the nesting phase in pink and the molt phase in light blue. The nesting phase was based on accelerometer and GPS data from neckband transmitters, while the molt phase was based on observations. In double-plotted actograms, the x-axis displays two consecutive days, and these consecutive days are also shown from top to bottom on the y-axis. Activity is shown in black, while transparency indicates inactivity.*

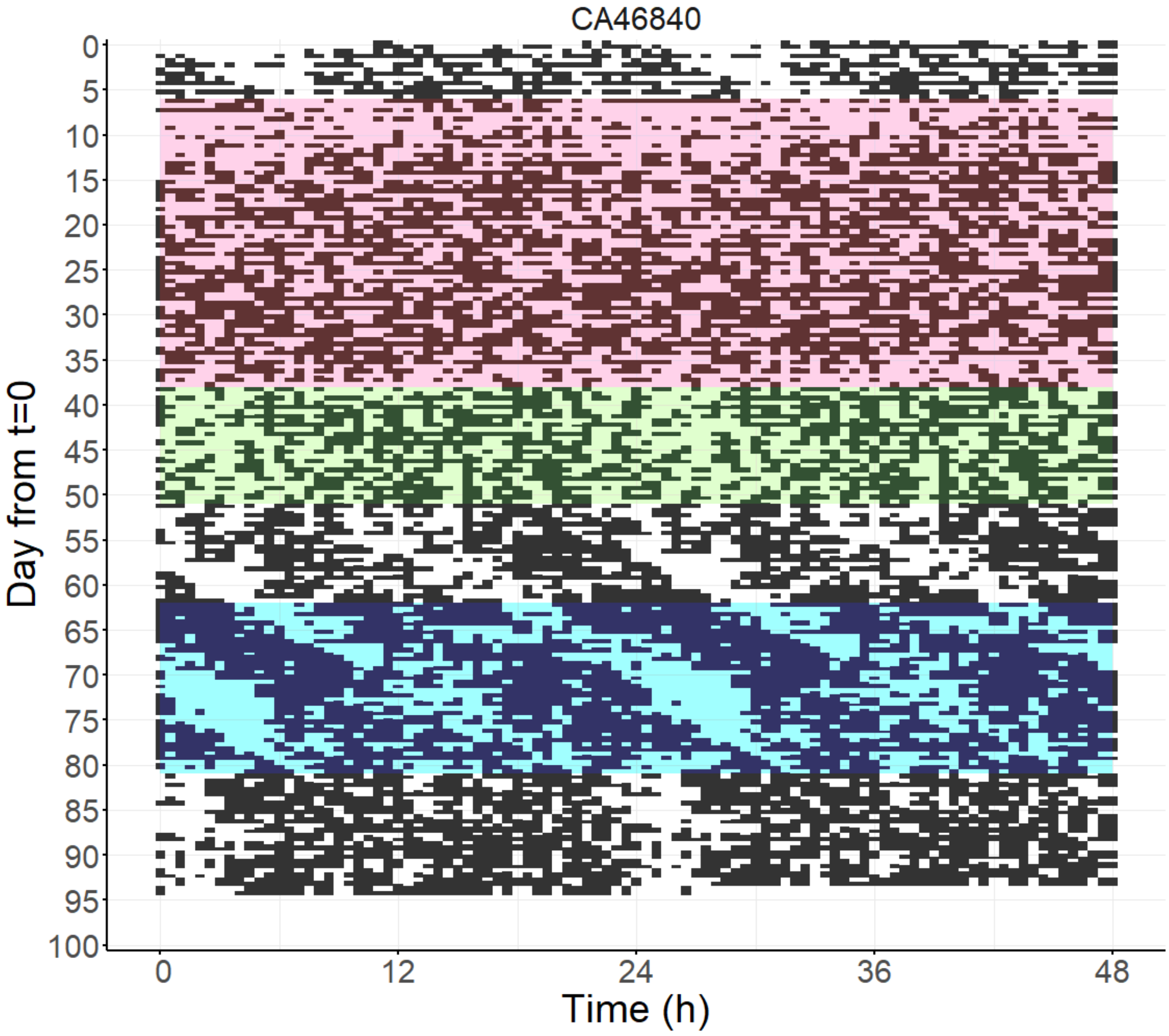

Figure 22: ***Rhythmicity in activity across the 2021 season*** *in male goose CA46840. Day t = 0 was 2021-05-28 11:58:35. Double-plotted actogram with the nesting phase in pink, the gosling phase in green and the molt phase in light blue. The nesting phase was based on accelerometer and GPS data from neckband transmitters, while the gosling and molt phases were based on observations. In double-plotted actograms, the x-axis displays two consecutive days, and these consecutive days are also shown from top to bottom on the y-axis. Activity is shown in black, while transparency indicates inactivity.*

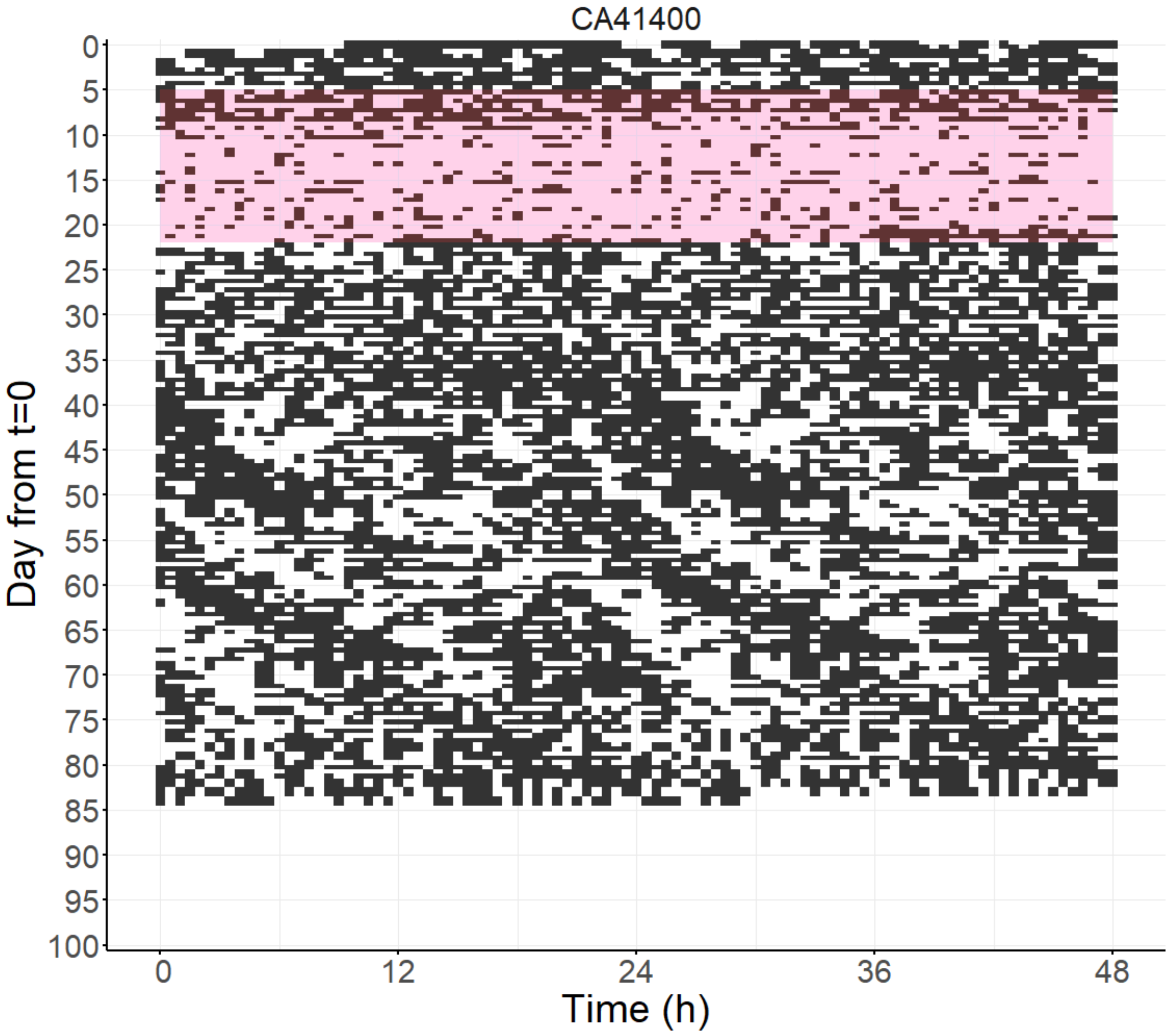

Figure 23: ***Rhythmicity in activity across the 2022 season*** *in female goose CA41400. Day t = 0 was 2022-05-23 10:17:53. Double-plotted actogram with the nesting phase in pink, the gosling phase in green and the molt phase in light blue. The nesting phase was based on accelerometer and GPS data from neckband transmitters, while the gosling and molt phases were based on observations. In double-plotted actograms, the x-axis displays two consecutive days, and these consecutive days are also shown from top to bottom on the y-axis. Activity is shown in black, while transparency indicates inactivity.*

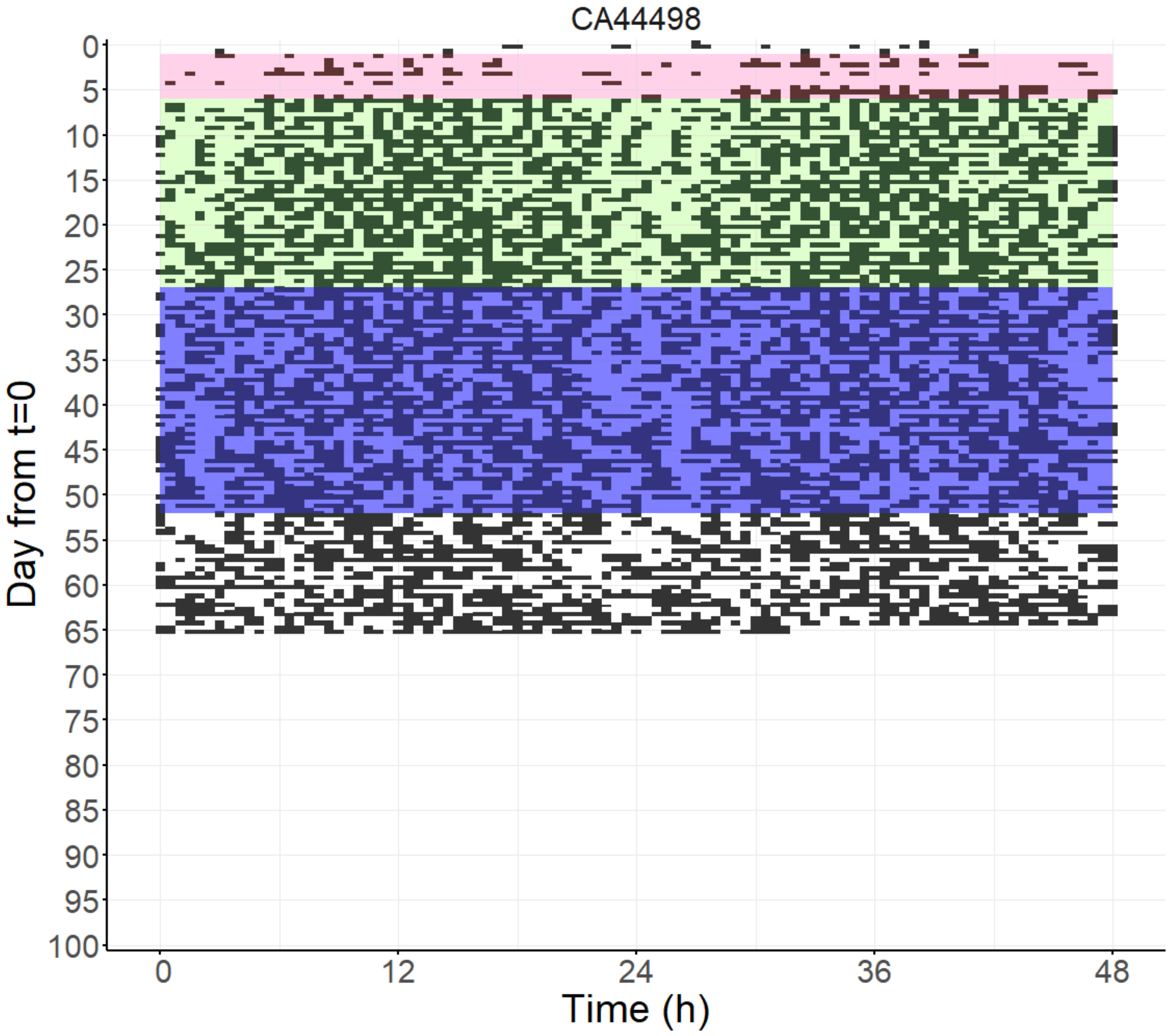

Figure 24: ***Rhythmicity in activity across the 2022 season*** *in female goose CA44498. Day t = 0 was on 2022-06-22 17:11:05. Double-plotted actogram with the nesting phase in pink, the gosling phase in green and the gosling & molt phase in dark blue. The nesting phase was based on accelerometer and GPS data from neckband transmitters, while the gosling and molt phases were based on observations. In double-plotted actograms, the x-axis displays two consecutive days, and these consecutive days are also shown from top to bottom on the y-axis. Activity is shown as black bars, while transparency indicates inactivity.*

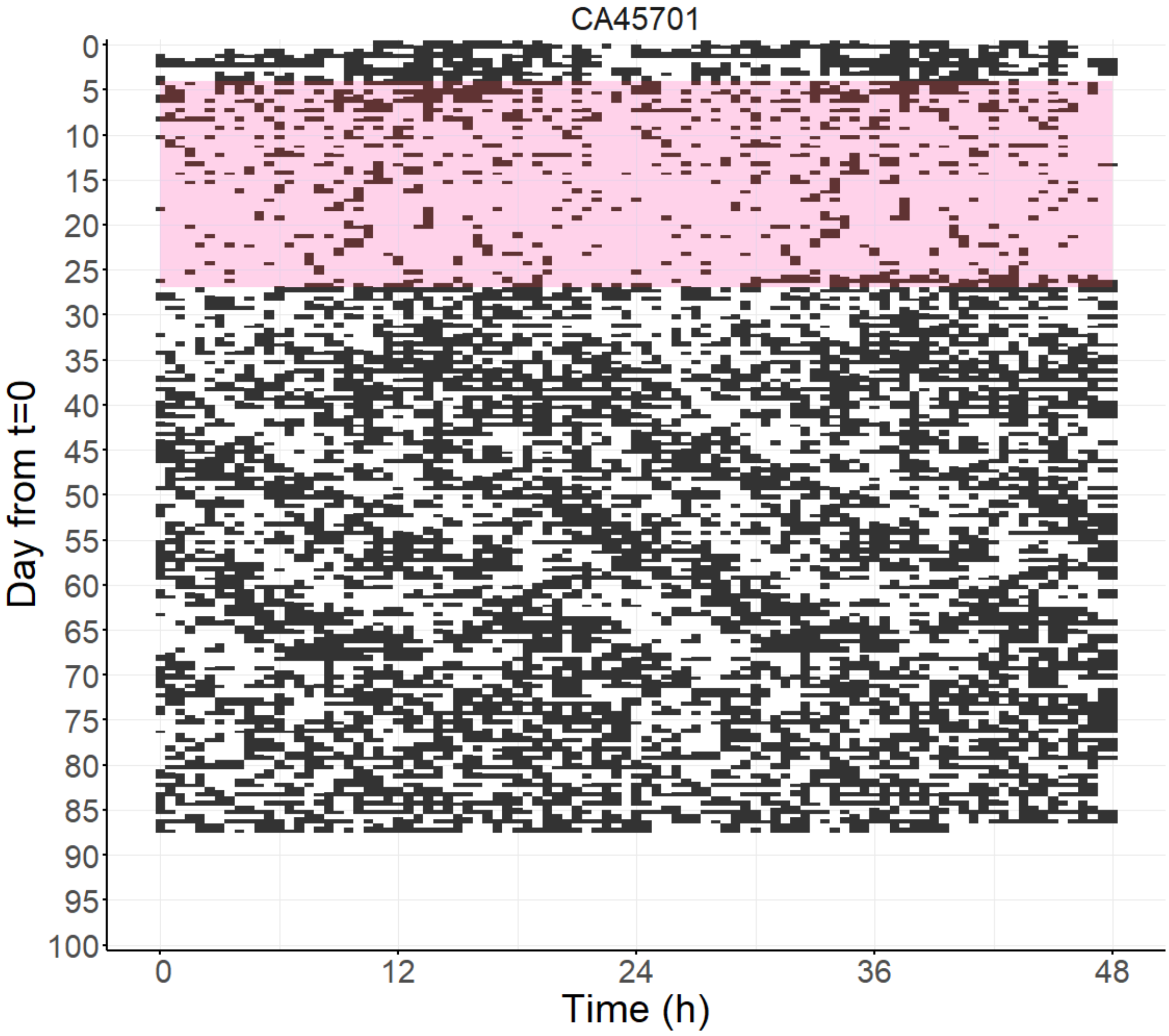

Figure 25: ***Rhythmicity in activity across the 2022 season*** *in female goose CA45701. Day t = 0 was on 2022-05-24 11:45:00. Double-plotted actogram with the nesting phase in pink. The nesting phase was based on accelerometer and GPS data from neckband transmitters. In double-plotted actograms, the x-axis displays two consecutive days, and these consecutive days are also shown from top to bottom on the y-axis. Activity is shown as black bars, while transparency indicates inactivity.*

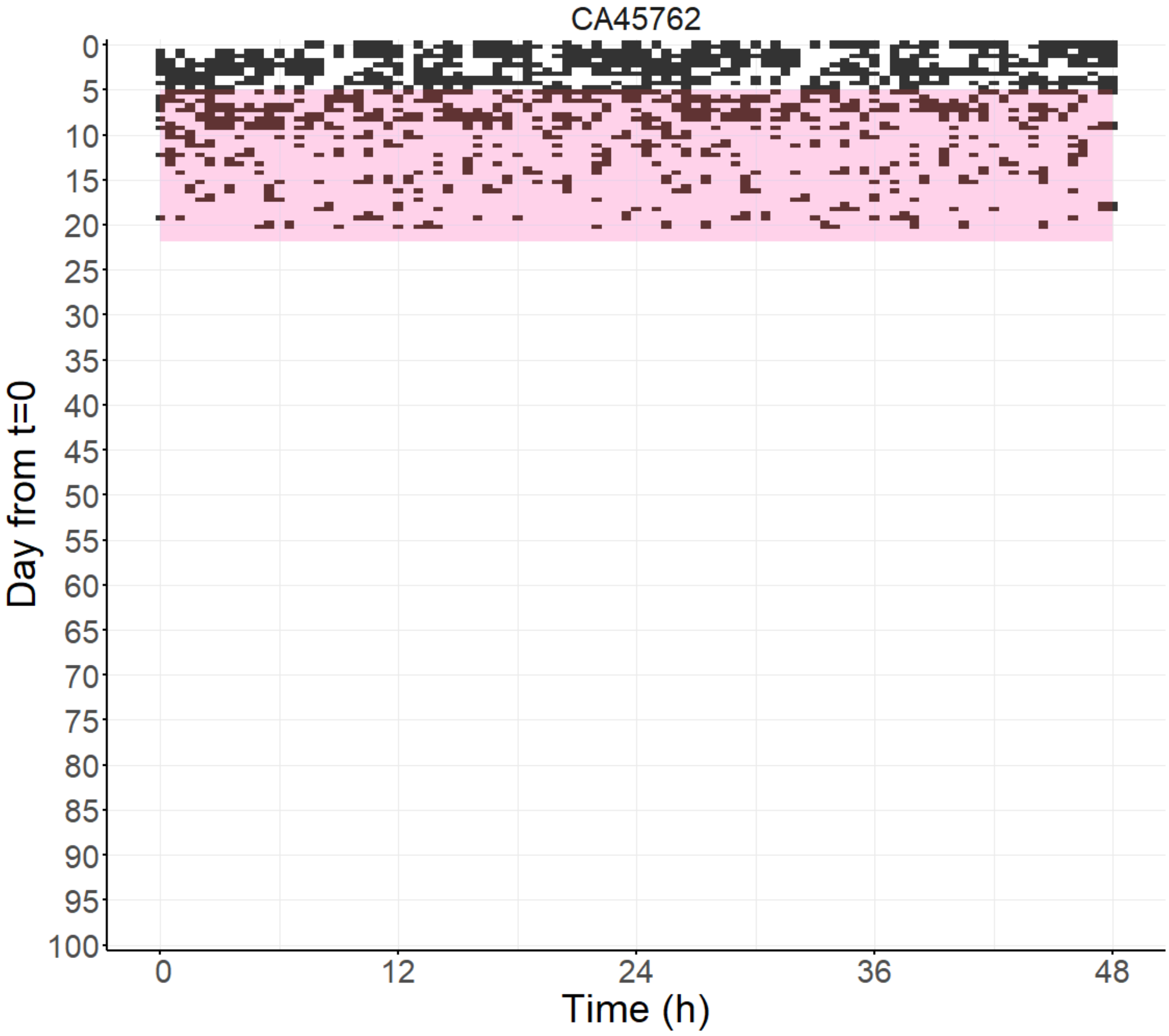

Figure 26: ***Rhythmicity in activity across the 2022 season*** *in female goose CA45762. Day t = 0 was on 2022-05-23 08:09:35. Double-plotted actogram with the nesting phase in pink. The nesting phase was based on accelerometer and GPS data from neckband transmitters. In double-plotted actograms, the x-axis displays two consecutive days, and these consecutive days are also shown from top to bottom on the y-axis. Activity is shown as black bars, while transparency indicates inactivity.*

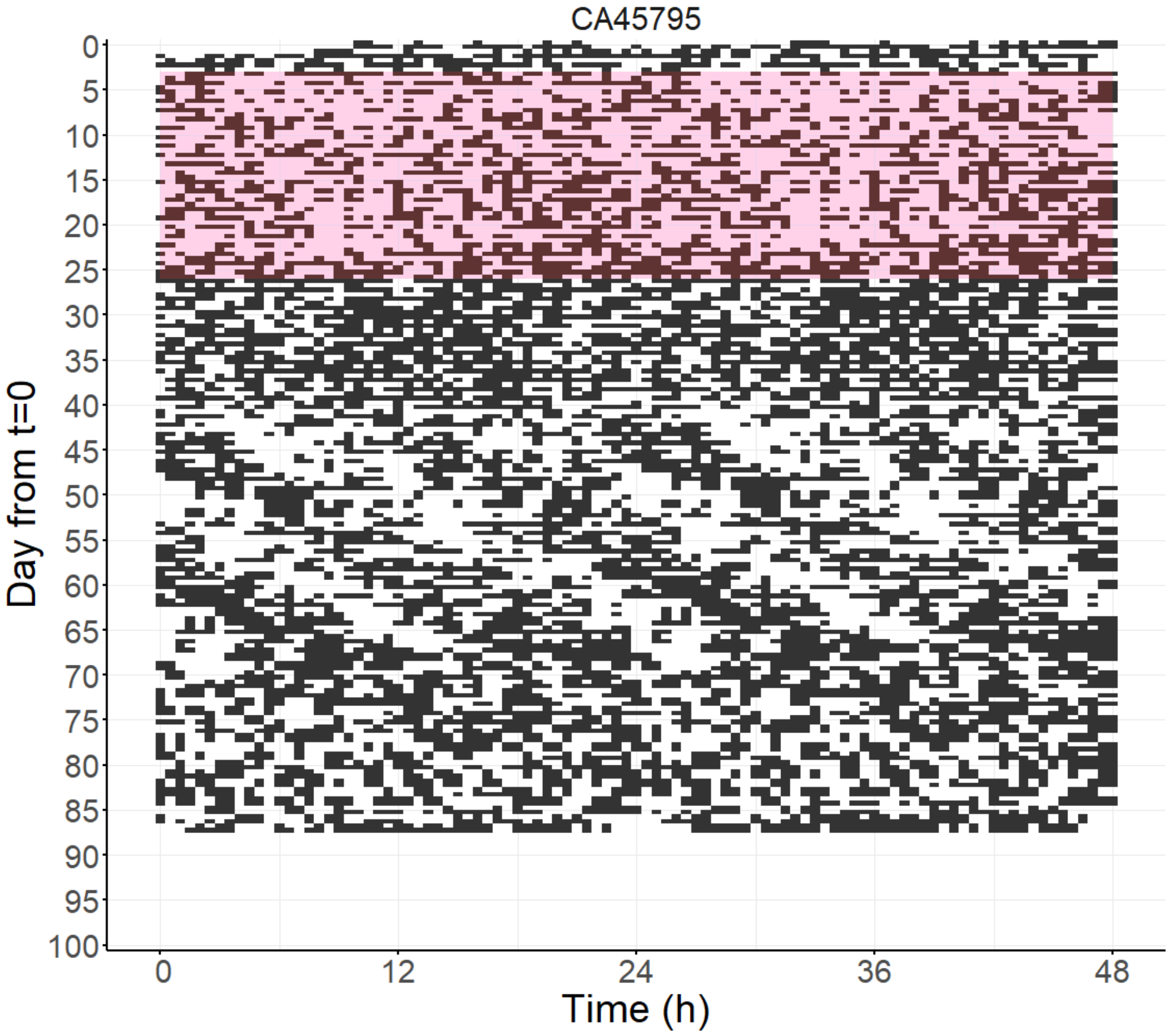

Figure 27: ***Rhythmicity in activity across the 2022 season*** *in male goose CA45795. Day t = 0 was on 2022-05-23 10:37:43. Double-plotted actogram with the nesting phase in pink. The nesting phase was based on accelerometer and GPS data from neckband transmitters. In double-plotted actograms, the x-axis displays two consecutive days, and these consecutive days are also shown from top to bottom on the y-axis. Activity is shown as black bars, while transparency indicates inactivity.*

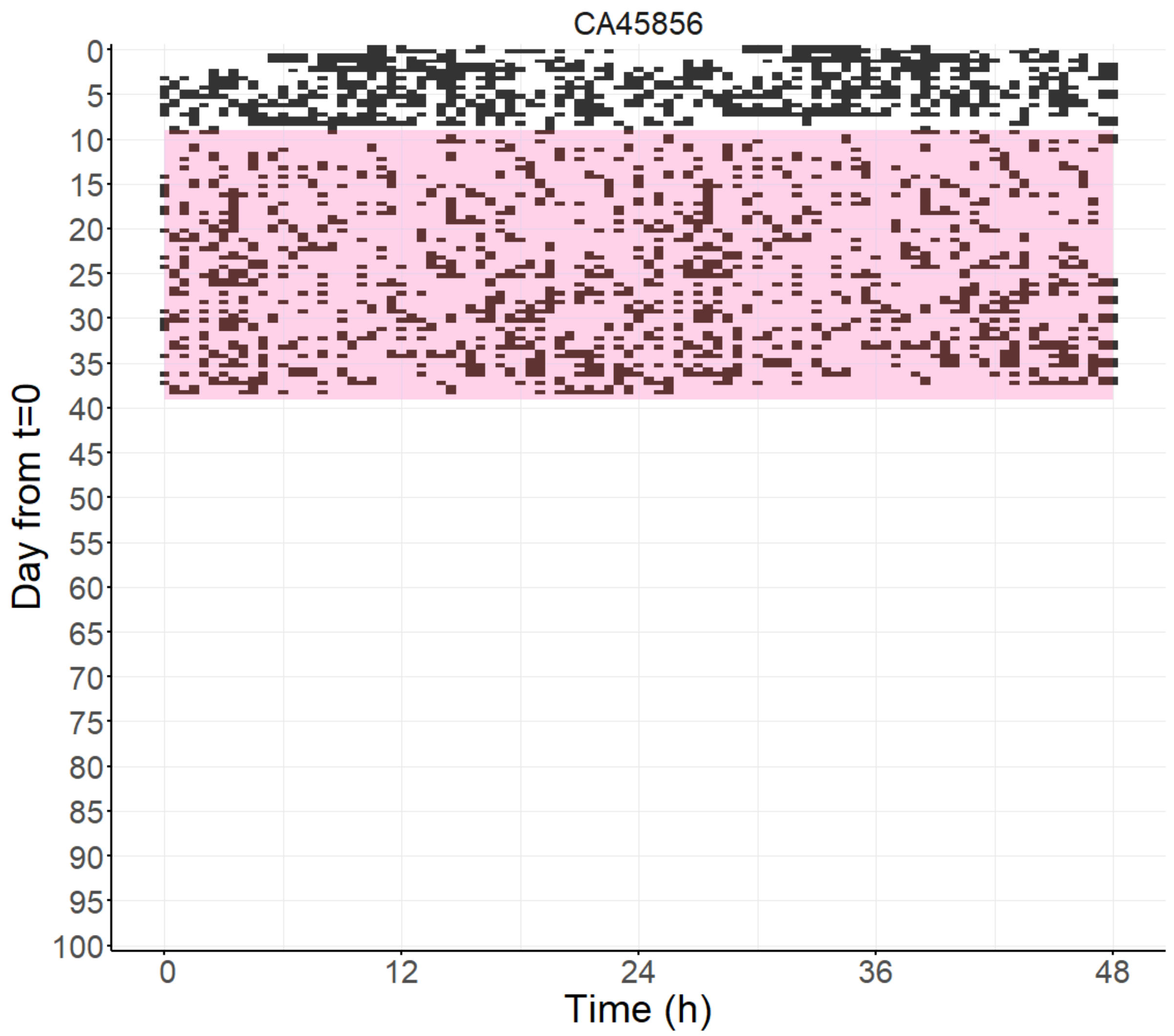

*Figure 28: **Rhythmicity in activity across the 2022 season** in male goose CA45856. Day t = 0 was on 2022-05-21 11:23:13. Double-plotted actogram with the nesting phase in pink. The nesting phase was based on accelerometer and GPS data from neckband transmitters. In double-plotted actograms, the x-axis displays two consecutive days, and these consecutive days are also shown from top to bottom on the y-axis. Activity is shown as black bars, while transparency indicates inactivity.*

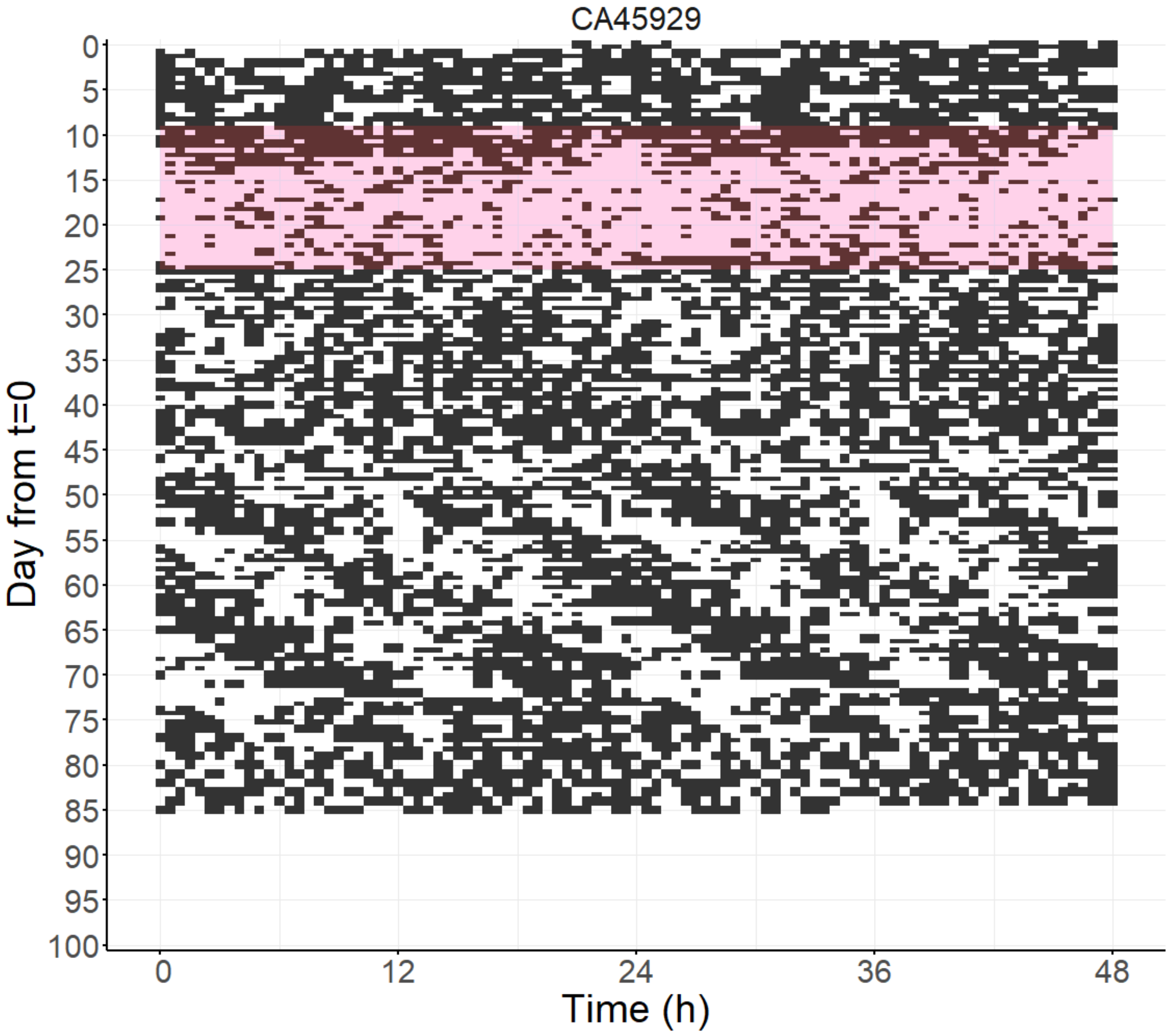

Figure 29: ***Rhythmicity in activity across the 2022 season*** *in female goose CA45929. Day t = 0 was on 2022-05-20 21:42:46. Double-plotted actogram with the nesting phase in pink. The nesting phase was based on accelerometer and GPS data from neckband transmitters. In double-plotted actograms, the x-axis displays two consecutive days, and these consecutive days are also shown from top to bottom on the y-axis. Activity is shown as black bars, while transparency indicates inactivity.*

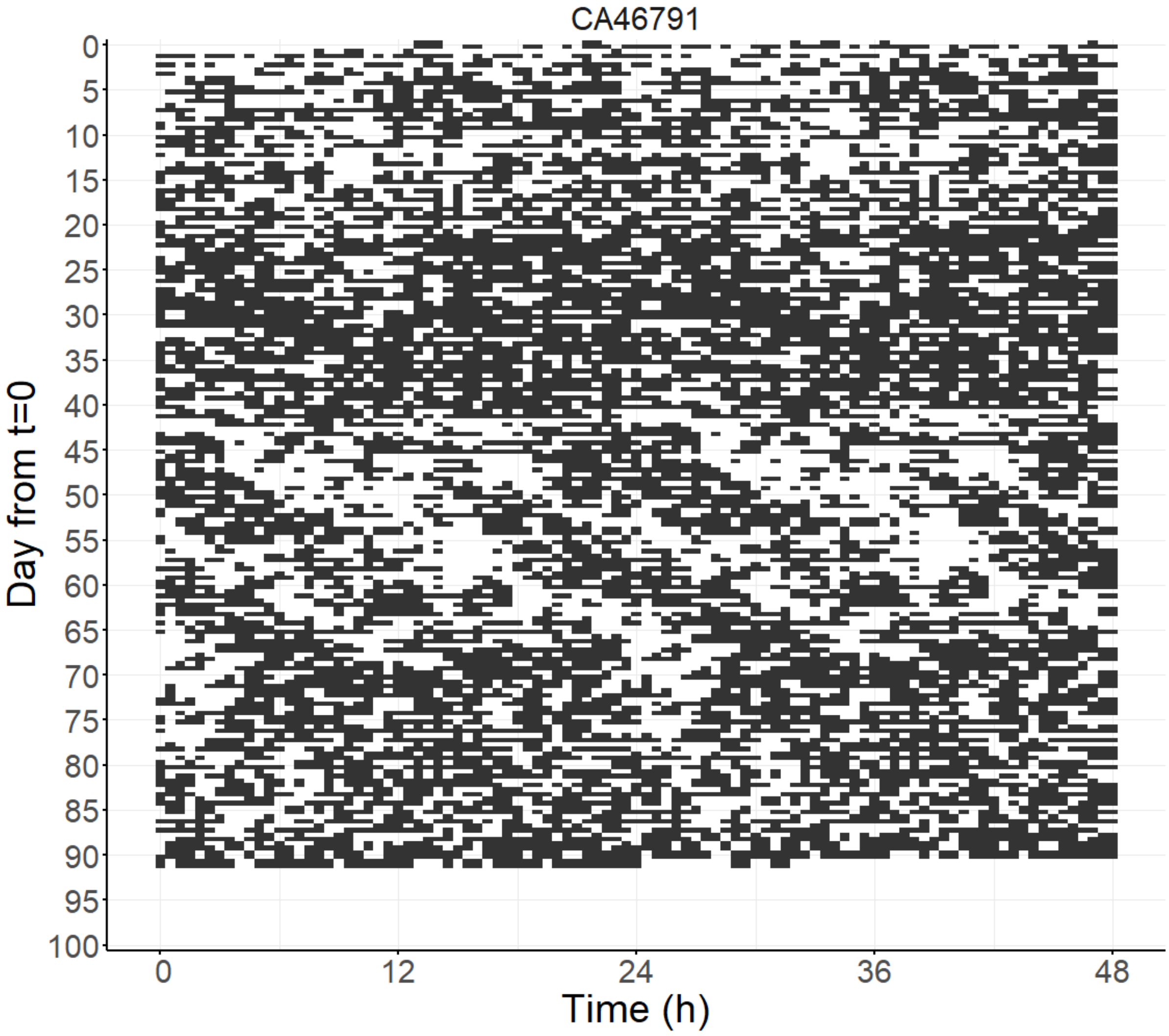

Figure 30: ***Rhythmicity in activity across the 2022 season*** *in male goose CA46791. Day t = 0 was on 2022-05-20 13:37:49. We had no observations on the nesting, gosling or molt phase of this goose. In double-plotted actograms, the x-axis displays two consecutive days, and these consecutive days are also shown from top to bottom on the y-axis. Activity is shown as black bars, while transparency indicates inactivity.*

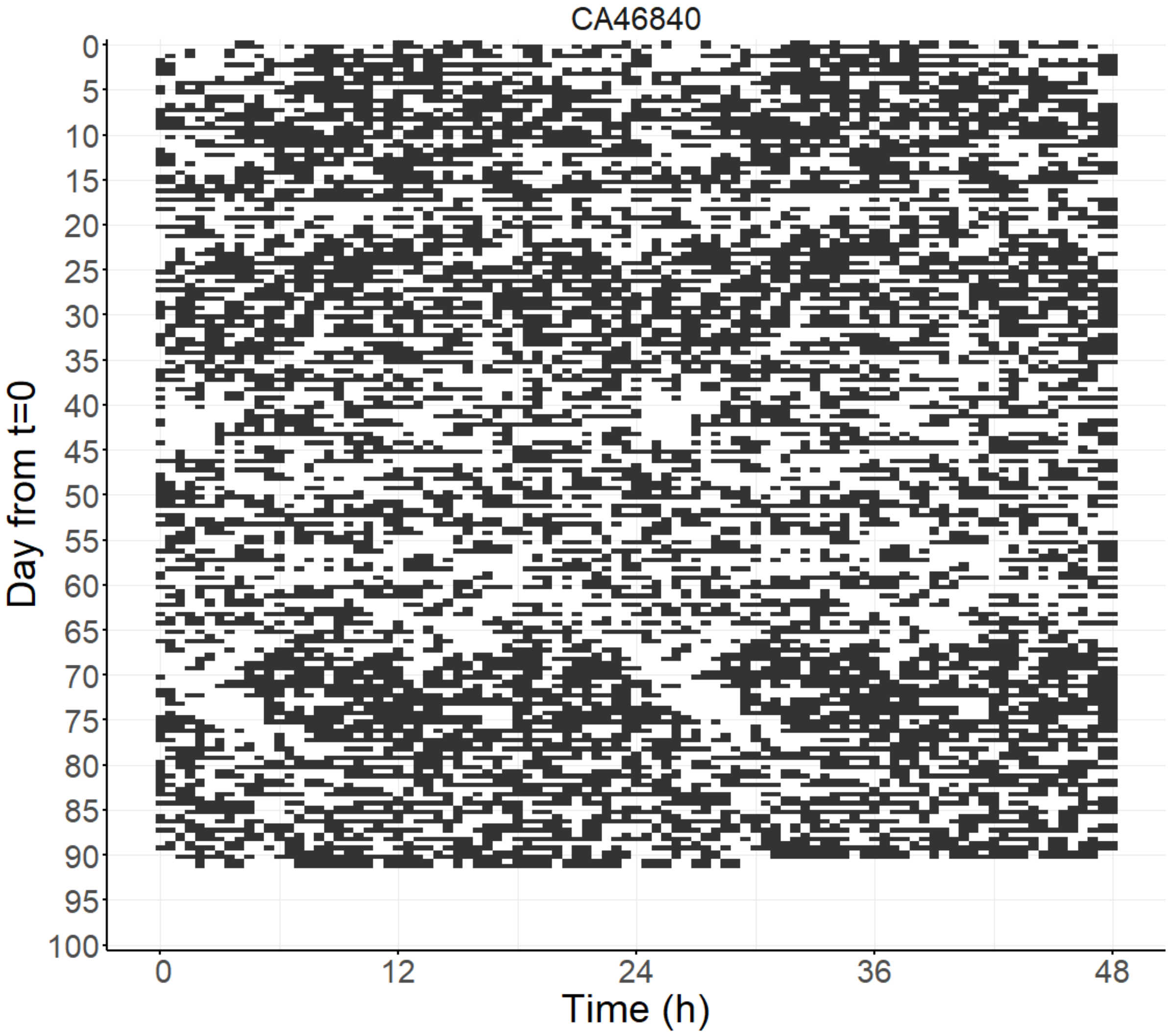

Figure 31: ***Rhythmicity in activity across the 2022 season*** *in male goose CA46840. Day t = 0 was on 2022-05-20 04:44:05. We had no observations on the nesting, gosling or molt phase of this goose. In double-plotted actograms, the x-axis displays two consecutive days, and these consecutive days are also shown from top to bottom on the y-axis. Activity is shown as black bars, while transparency indicates inactivity.*

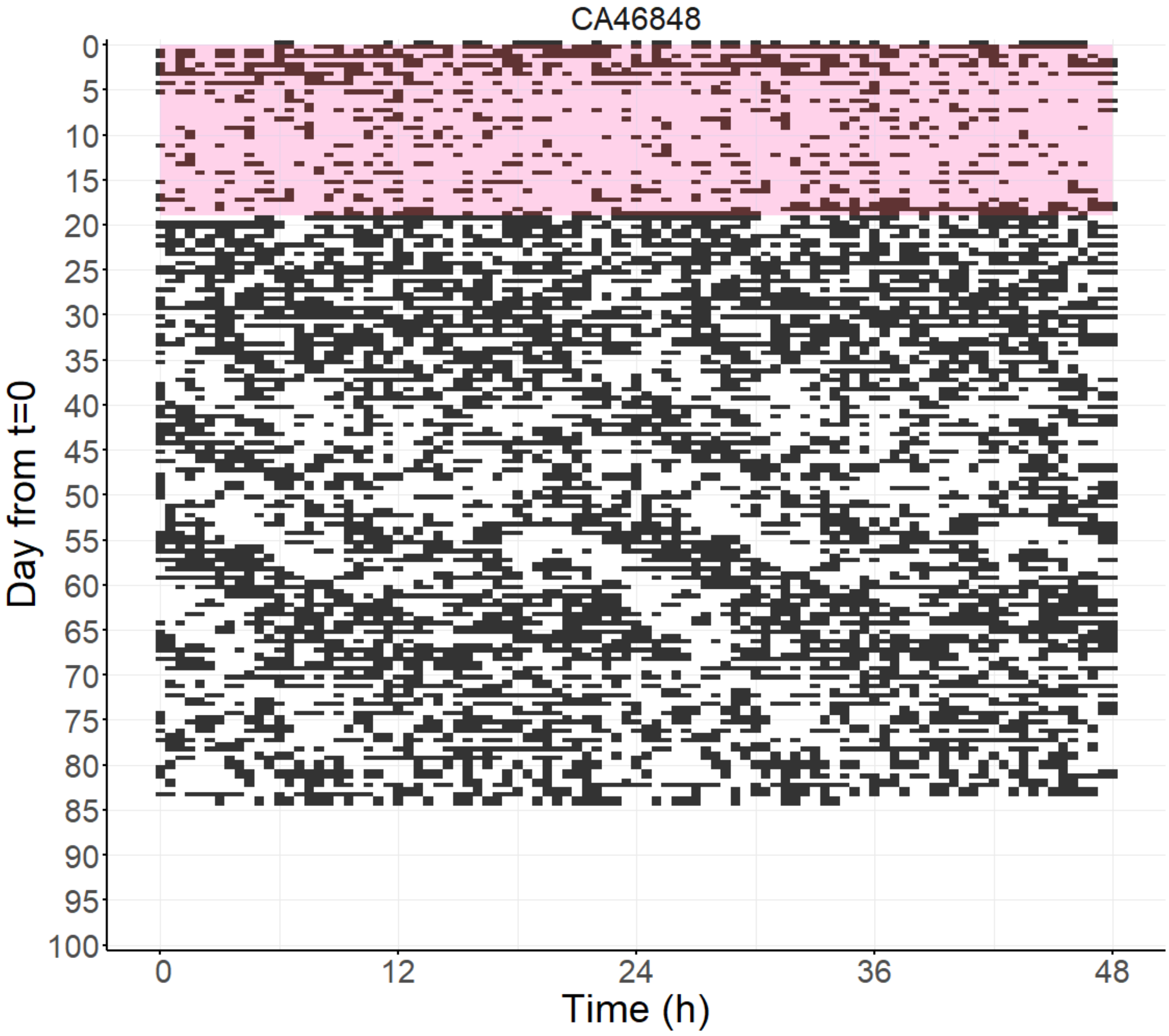

Figure 32: ***Rhythmicity in activity across the 2022 season*** *in female goose CA46848. Day t = 0 was on 2022-05-26 06:51:08. Double-plotted actogram with the nesting phase in pink. The nesting phase was based on accelerometer and GPS data from neckband transmitters. In double-plotted actograms, the x-axis displays two consecutive days, and these consecutive days are also shown from top to bottom on the y-axis. Activity is shown as black bars, while transparency indicates inactivity.*

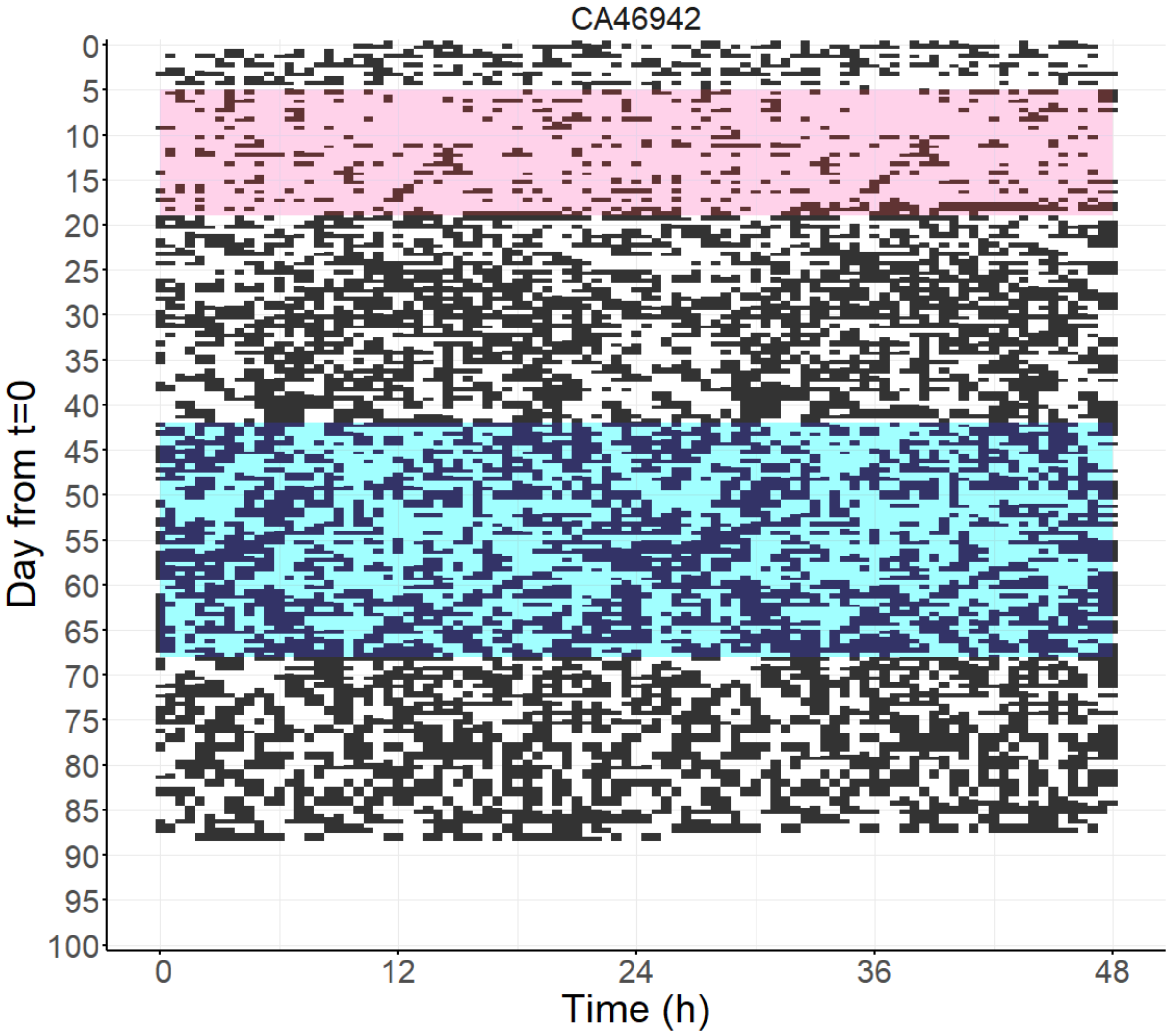

Figure 33: ***Rhythmicity in activity across the 2022 season*** *in male goose CA46942. Day t = 0 was on 2022-05-23 10:30:13. Double-plotted actogram with the nesting phase in pink and the molt phase in light blue. The nesting phase was based on accelerometer and GPS data from neckband transmitters, while the molt phase was based on observations. In double-plotted actograms, the x-axis displays two consecutive days, and these consecutive days are also shown from top to bottom on the y-axis. Activity is shown as black bars, while transparency indicates inactivity.*

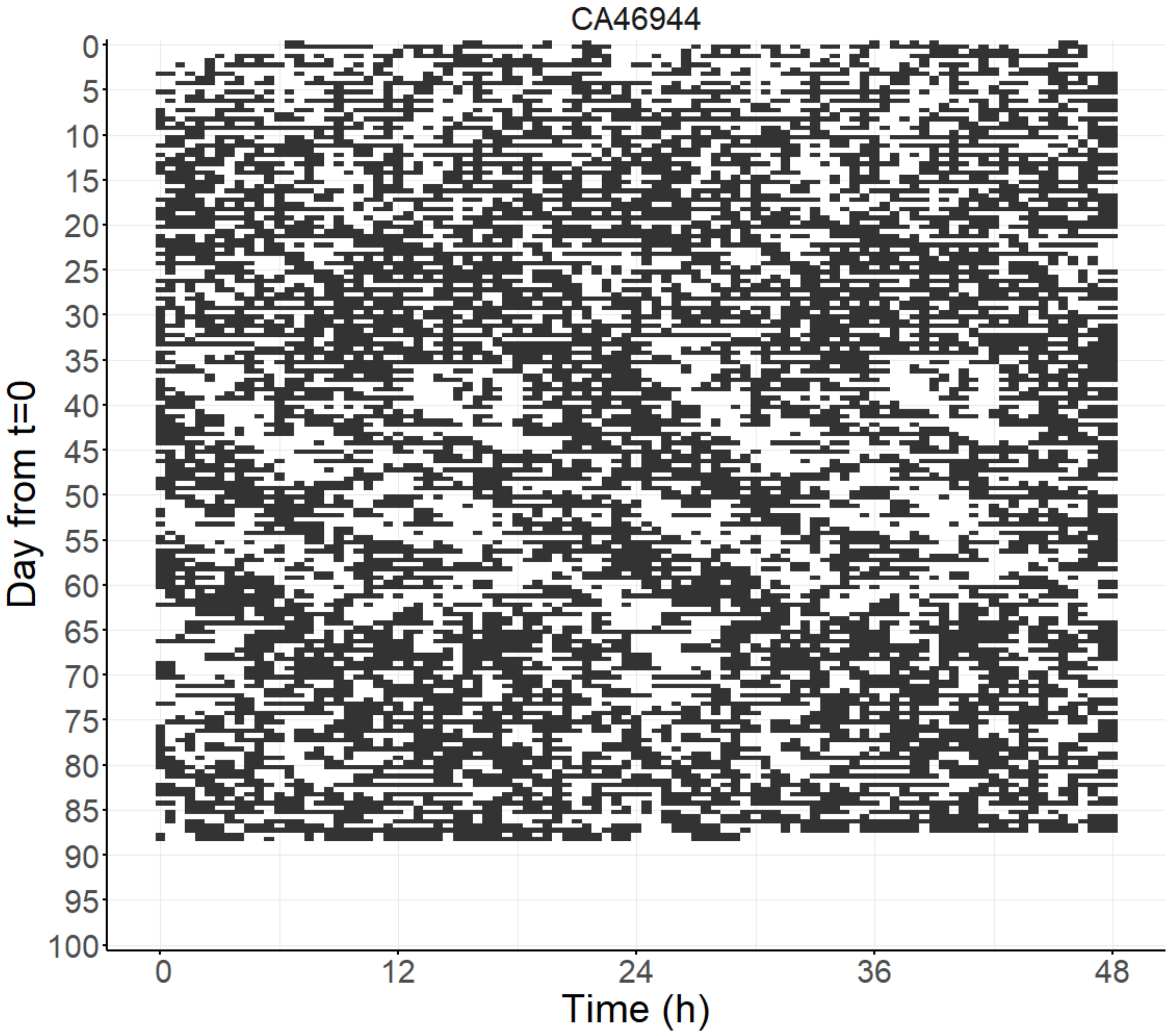

Figure 34: ***Rhythmicity in activity across the 2022 season*** *in male goose CA46944. Day t = 0 was on 2022-05-23 07:22:08. We had no observations on the nesting, gosling or molt phase of this goose. In double-plotted actograms, the x-axis displays two consecutive days, and these consecutive days are also shown from top to bottom on the y-axis. Activity is shown as black bars, while transparency indicates inactivity.*

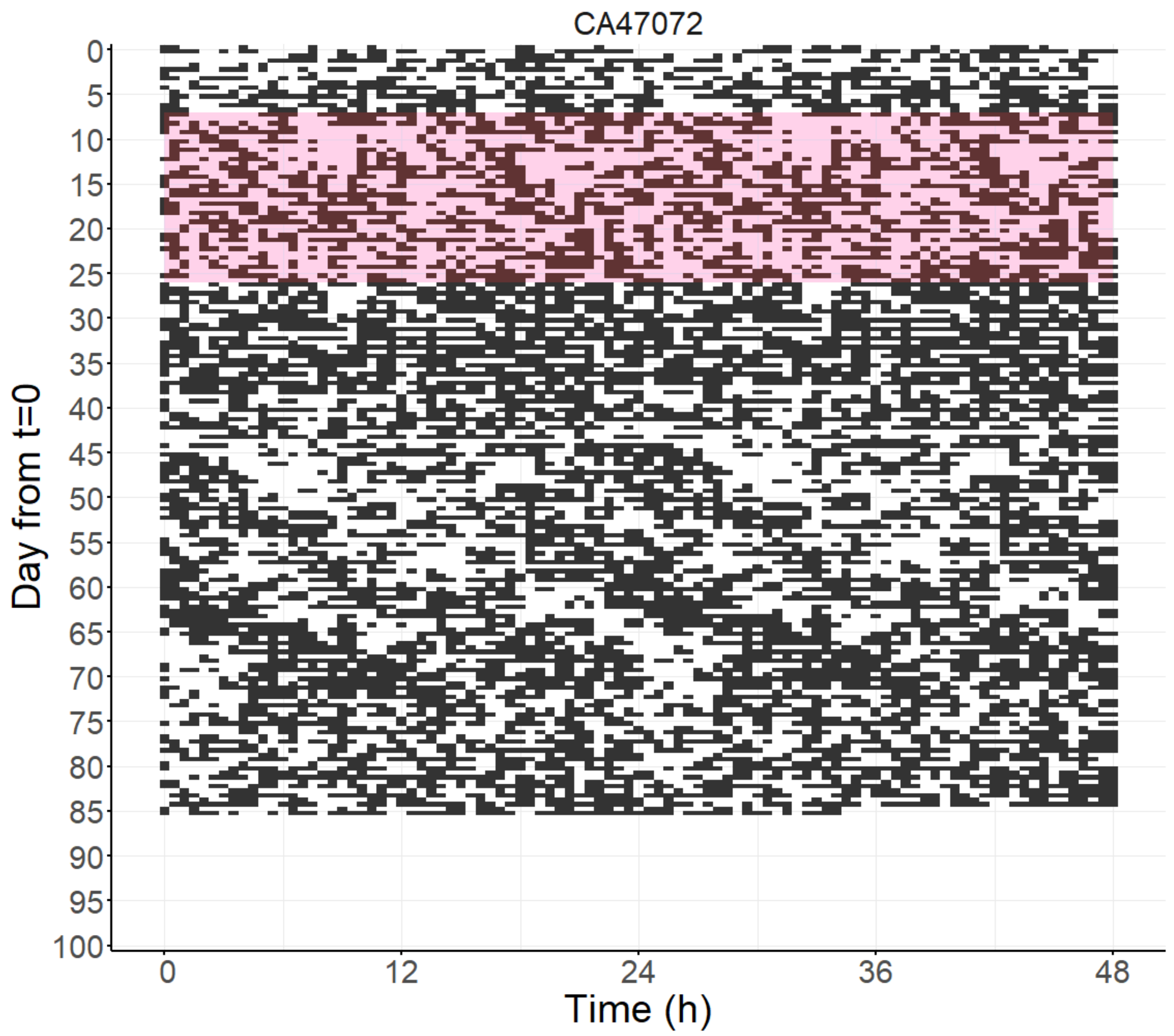

Figure 35: ***Rhythmicity in activity across the 2022 season*** *in male goose CA47072. Day t = 0 was on 2022-05-20 00:48:35. Double-plotted actogram with the nesting phase in pink. The nesting phase was based on accelerometer and GPS data from neckband transmitters. In double-plotted actograms, the x-axis displays two consecutive days, and these consecutive days are also shown from top to bottom on the y-axis. Activity is shown as black bars, while transparency indicates inactivity.*

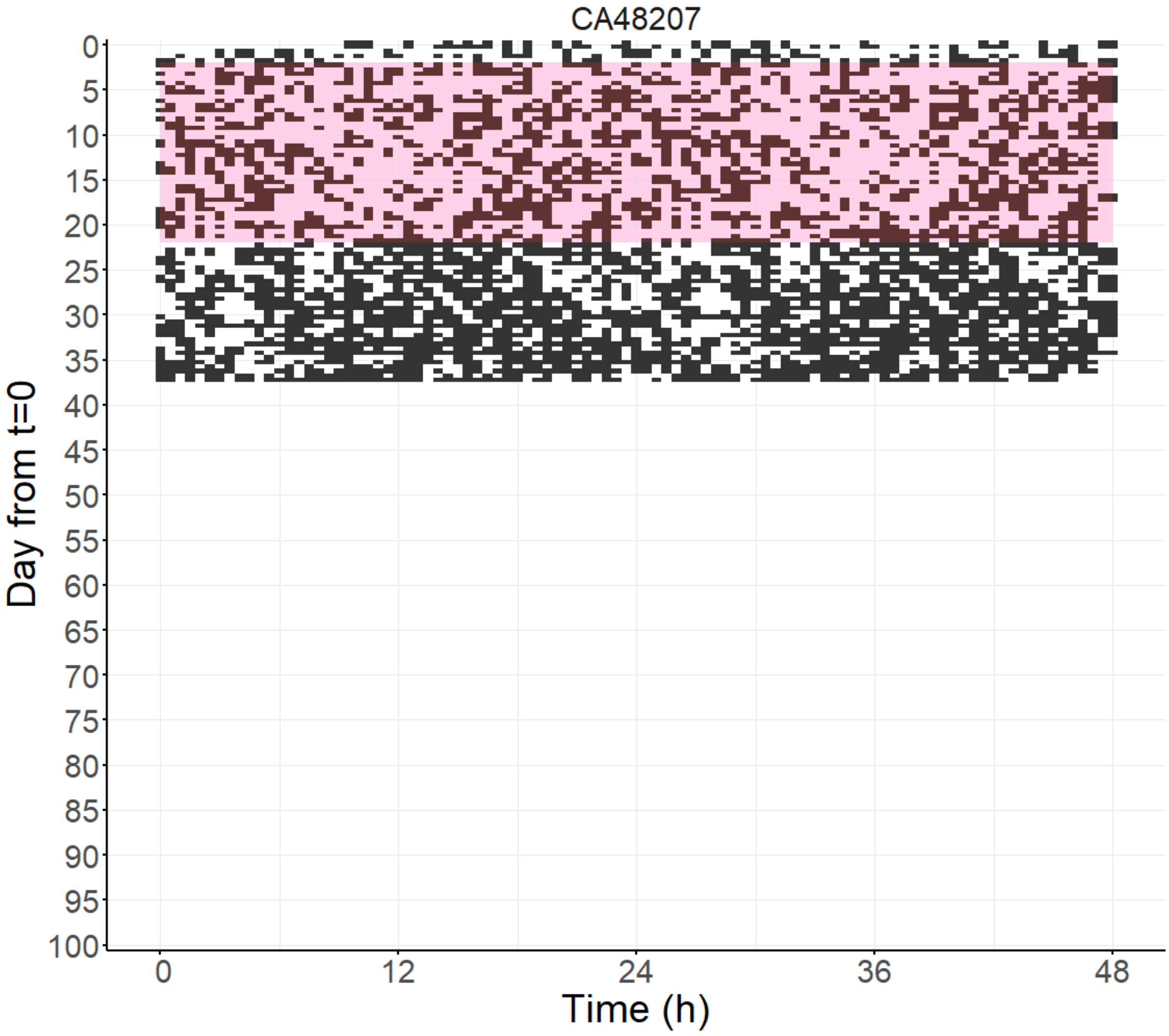

Figure 36: ***Rhythmicity in activity across the 2022 season*** *in male goose CA48207. Day t = 0 was on 2022-05-23 10:09:23. Double-plotted actogram with the nesting phase in pink. The nesting phase was based on accelerometer and GPS data from neckband transmitters. In double-plotted actograms, the x-axis displays two consecutive days, and these consecutive days are also shown from top to bottom on the y-axis. Activity is shown as black bars, while transparency indicates inactivity.*

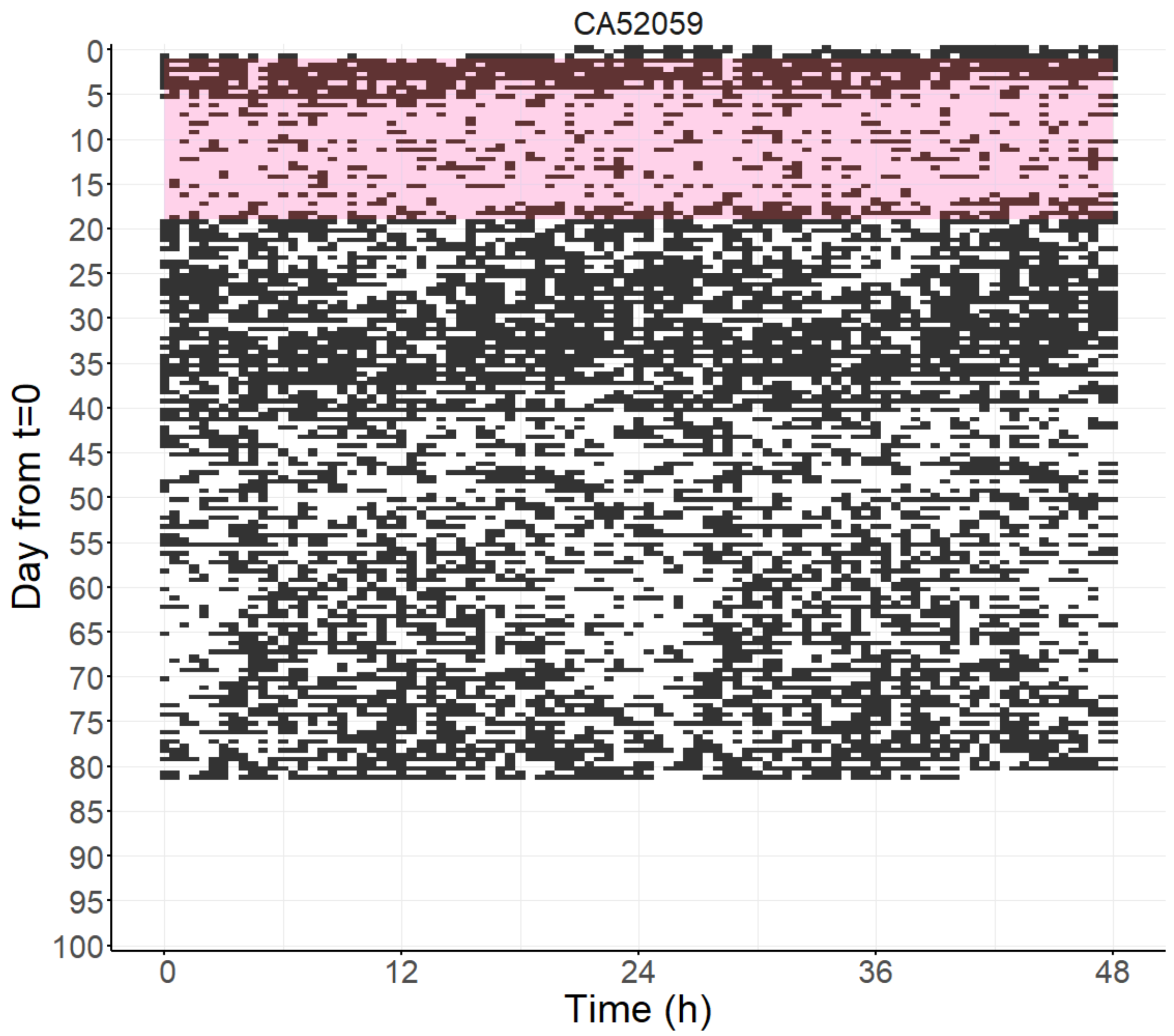

Figure 37: ***Rhythmicity in activity across the 2022 season*** *in female goose CA52059. Day t = 0 was on 2022-05-25 21:58:00. Double-plotted actogram with the nesting phase in pink. The nesting phase was based on accelerometer and GPS data from neckband transmitters. In double-plotted actograms, the x-axis displays two consecutive days, and these consecutive days are also shown from top to bottom on the y-axis. Activity is shown as black bars, while transparency indicates inactivity.*

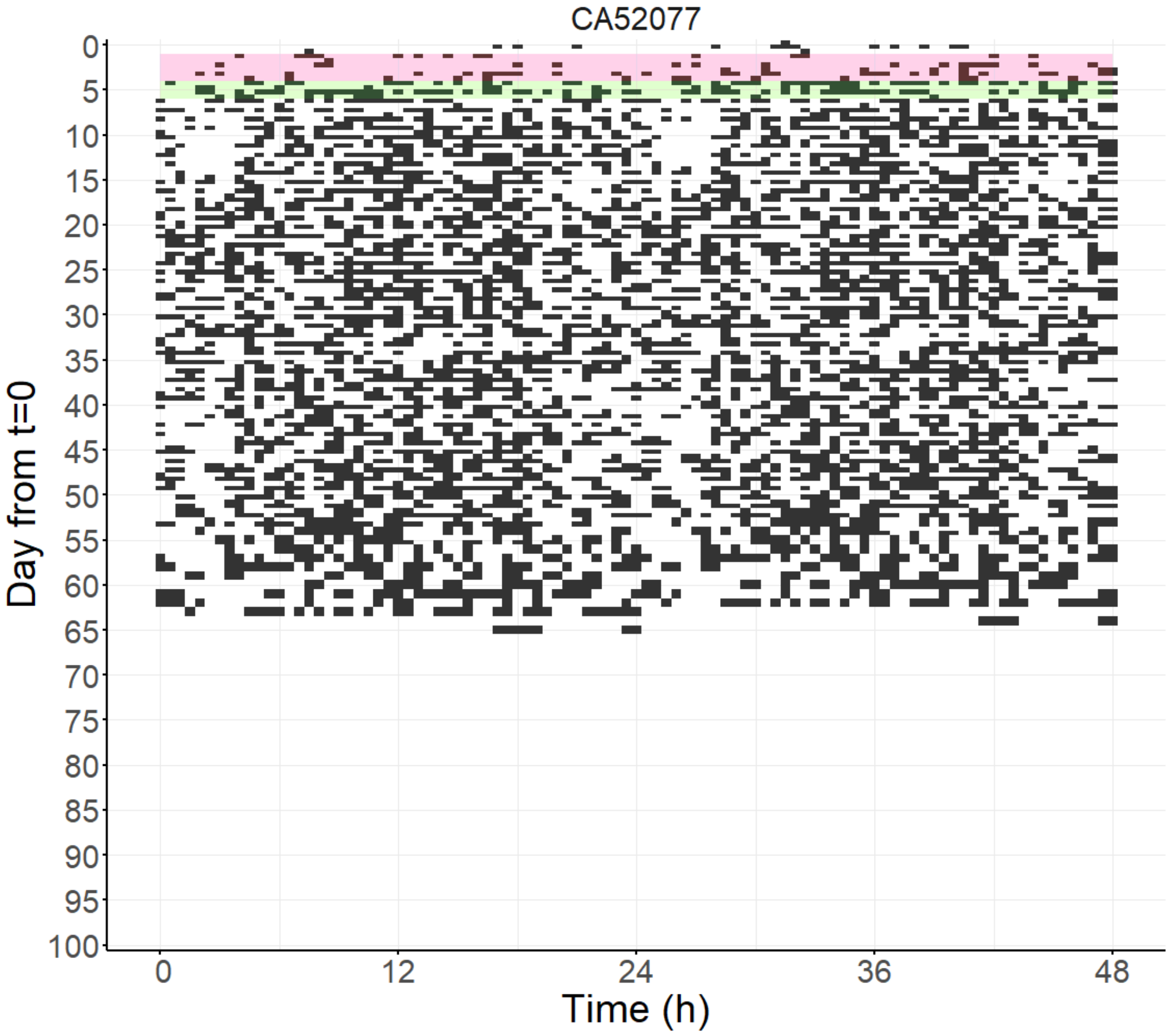

Figure 38: ***Rhythmicity in activity across the 2022 season*** *in female goose CA52077. Day t = 0 was on 2022-06-22 16:52:08. Double-plotted actogram with the nesting phase in pink, and the gosling phase in green. The nesting phase was based on accelerometer and GPS data from neckband transmitters, while the gosling phase was based on observations. In double-plotted actograms, the x-axis displays two consecutive days, and these consecutive days are also shown from top to bottom on the y-axis. Activity is shown as black bars, while transparency indicates inactivity.*

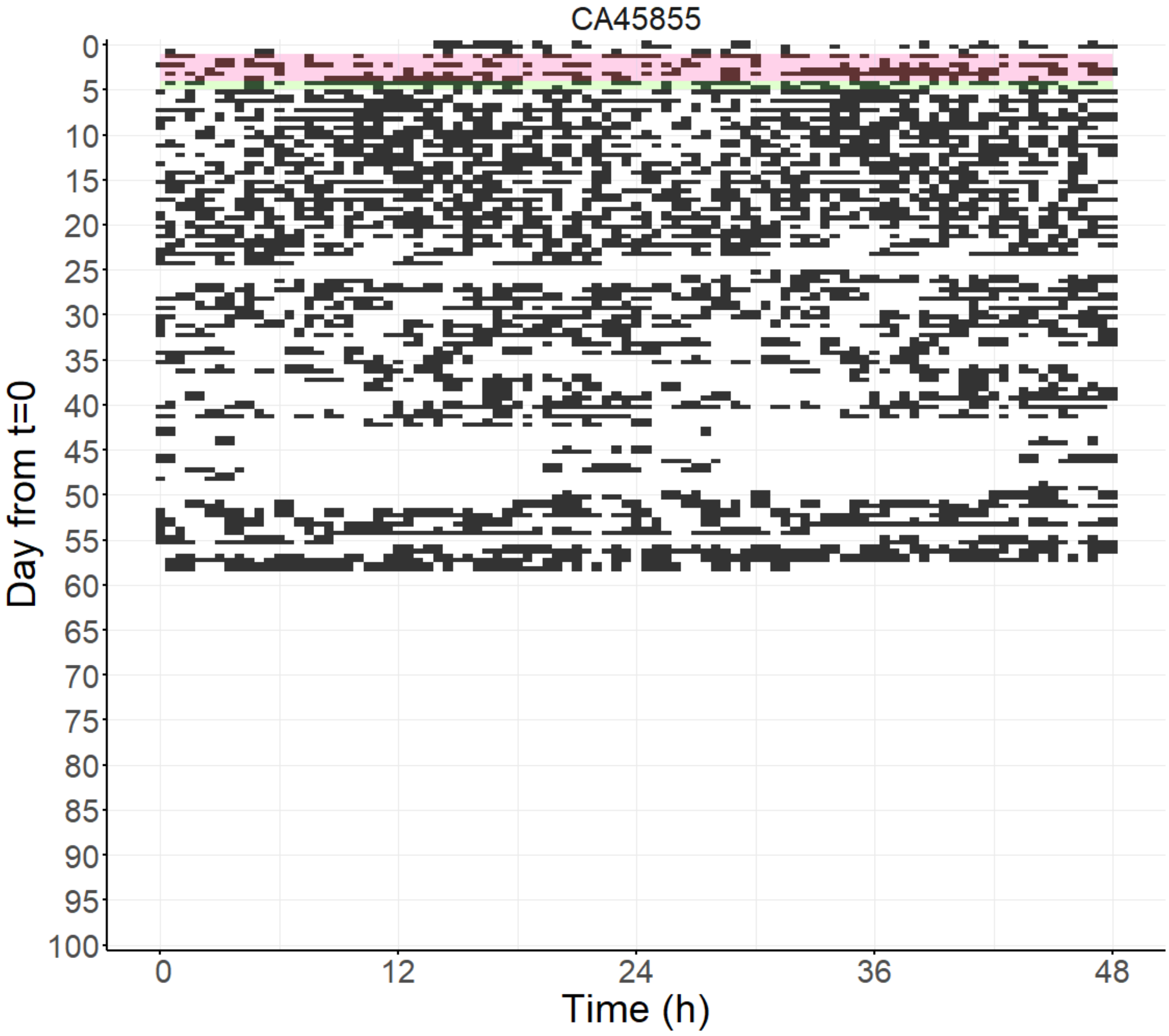

Figure 39: ***Rhythmicity in activity across the 2022 season*** *in male goose CA45855. Day t = 0 was on 2022-06-22 14:43:05. Double-plotted actogram with the nesting phase in pink, and the gosling phase in green. The nesting phase was based on accelerometer and GPS data from neckband transmitters, while the gosling phase was based on observations. In double-plotted actograms, the x-axis displays two consecutive days, and these consecutive days are also shown from top to bottom on the y-axis. Activity is shown as black bars, while transparency indicates inactivity.*

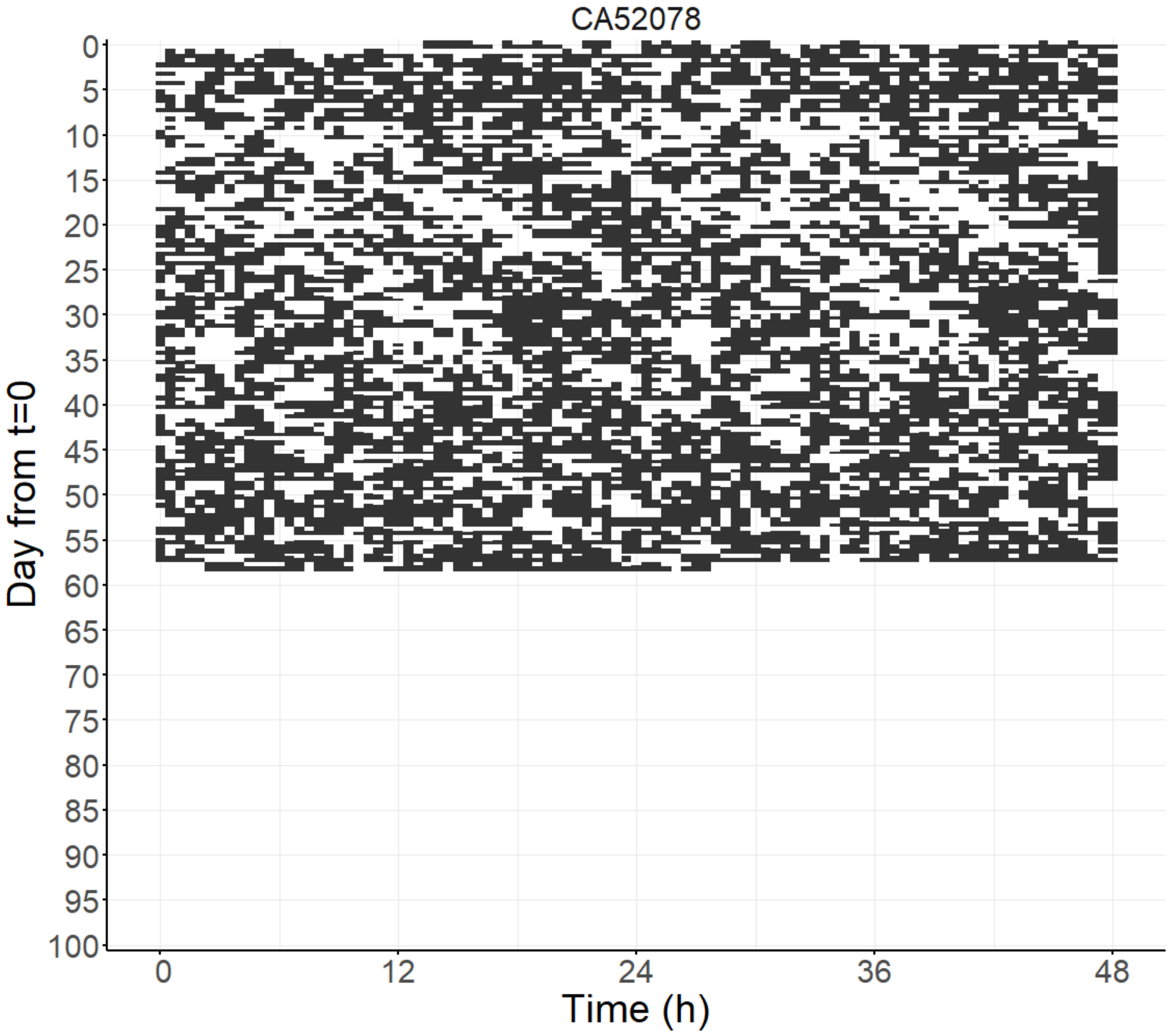

Figure 40: ***Rhythmicity in activity across the 2022 season*** *in female goose CA52078. Day t = 0 was on 2022-06-28 14:28:25. We had no observations on the nesting, gosling or molt phase of this goose. In double-plotted actograms, the x-axis displays two consecutive days, and these consecutive days are also shown from top to bottom on the y-axis. Activity is shown as black bars, while transparency indicates inactivity.*

Seasonal rhythmicity in activity: Periodograms

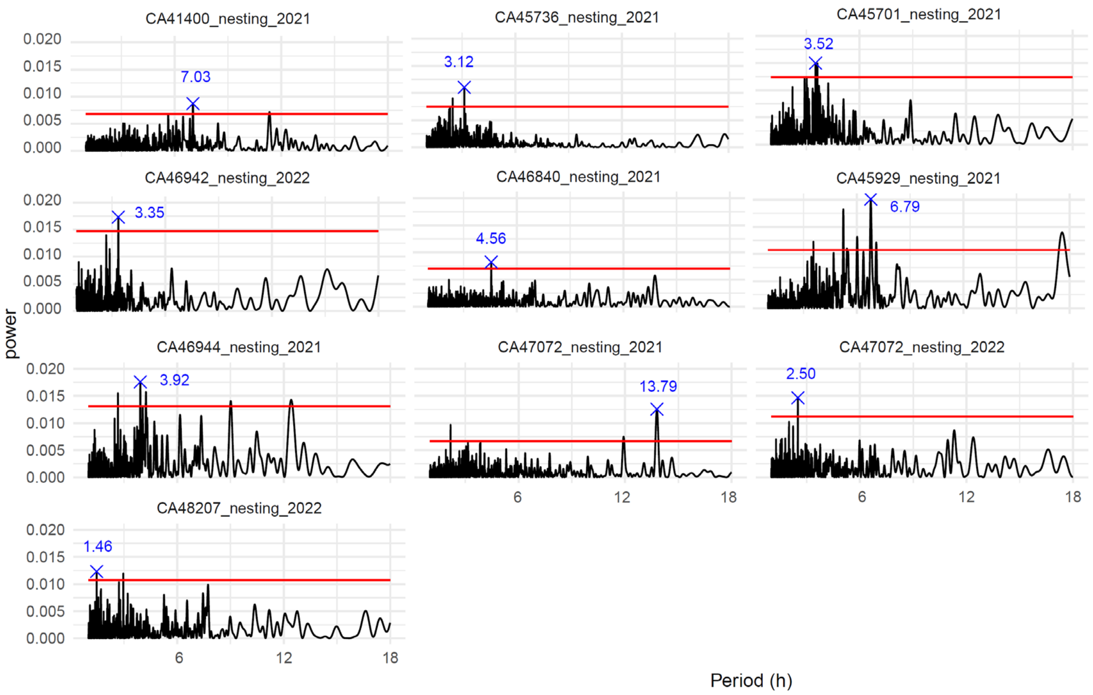

Figure 41: Lomb-Scargle periodograms showing ***ultradian peak period in activity during the nesting phase*** *for individual geese. The analysis identified periodicity between 1 and 18 hours. The headers indicate goose ID, year and breeding stage.*

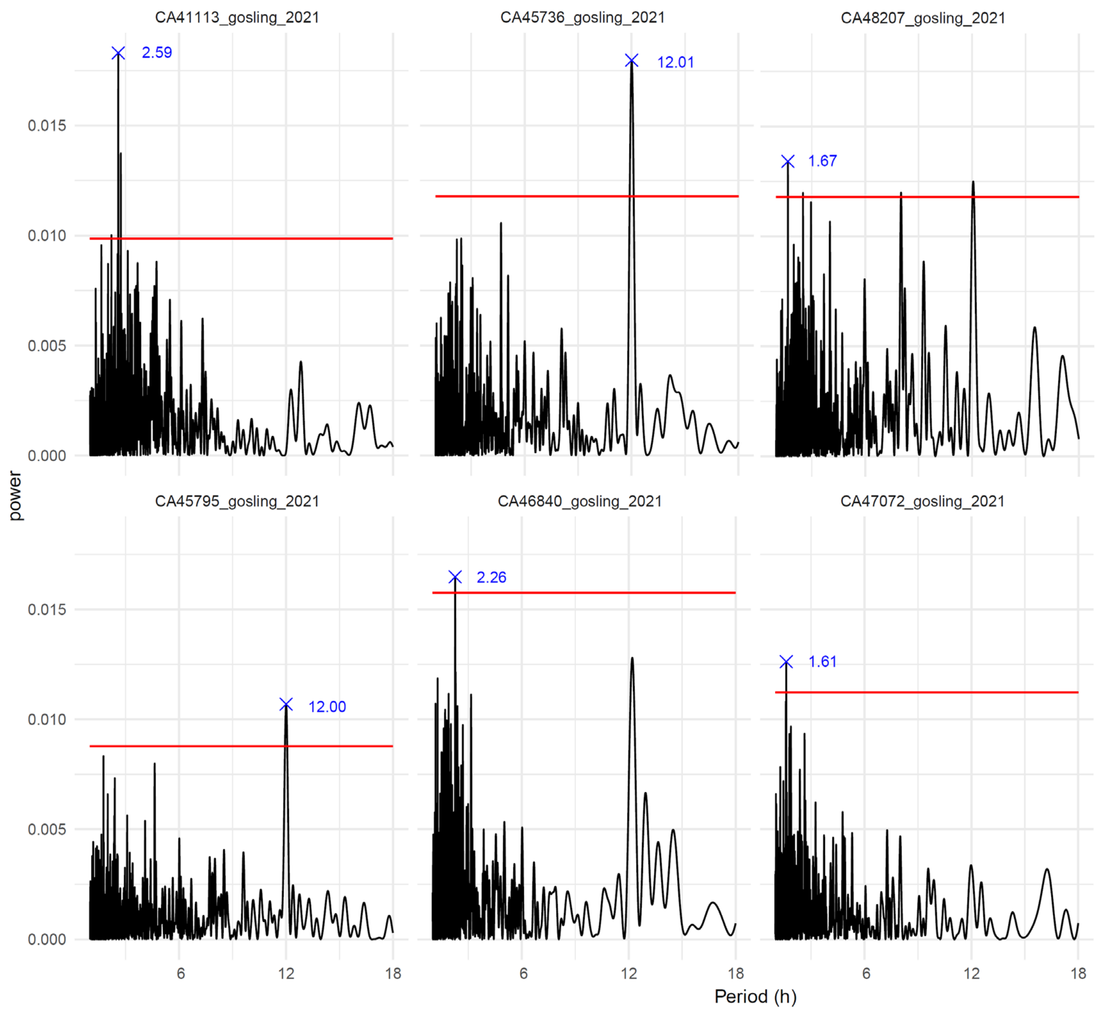

Figure 42: Lomb-Scargle periodograms showing ***ultradian peak period in activity during the gosling phase*** *for individual geese. The analysis identified periodicity between 1 and 18 hours. The headers indicate goose ID, year and breeding stage.*

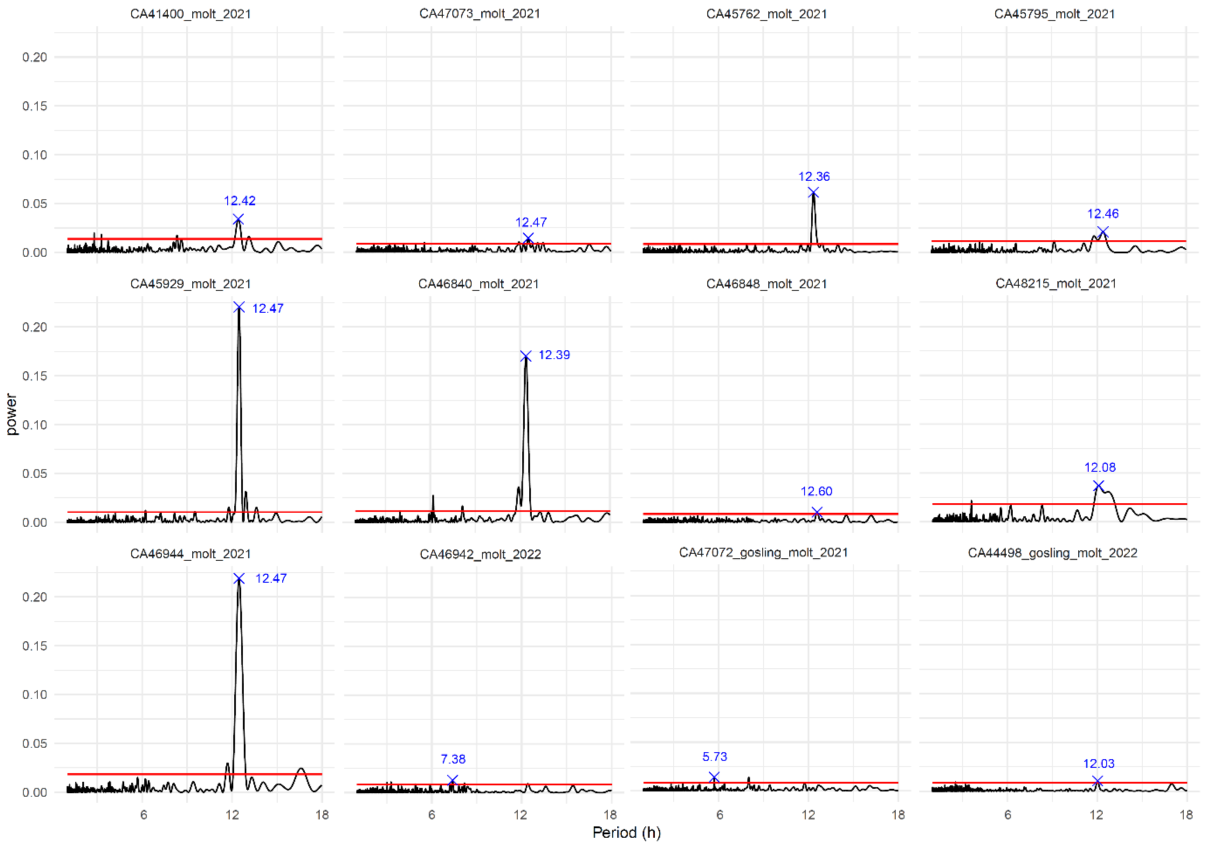

Figure 43: Lomb-Scargle periodograms showing ***ultradian peak period in activity during the molt phase*** *for individual geese. We pooled two goslings & molt phases with the molt phase data to increase sample size. The analysis identified periodicity between 1 and 18 hours. The headers indicate goose ID, year and breeding stage.*

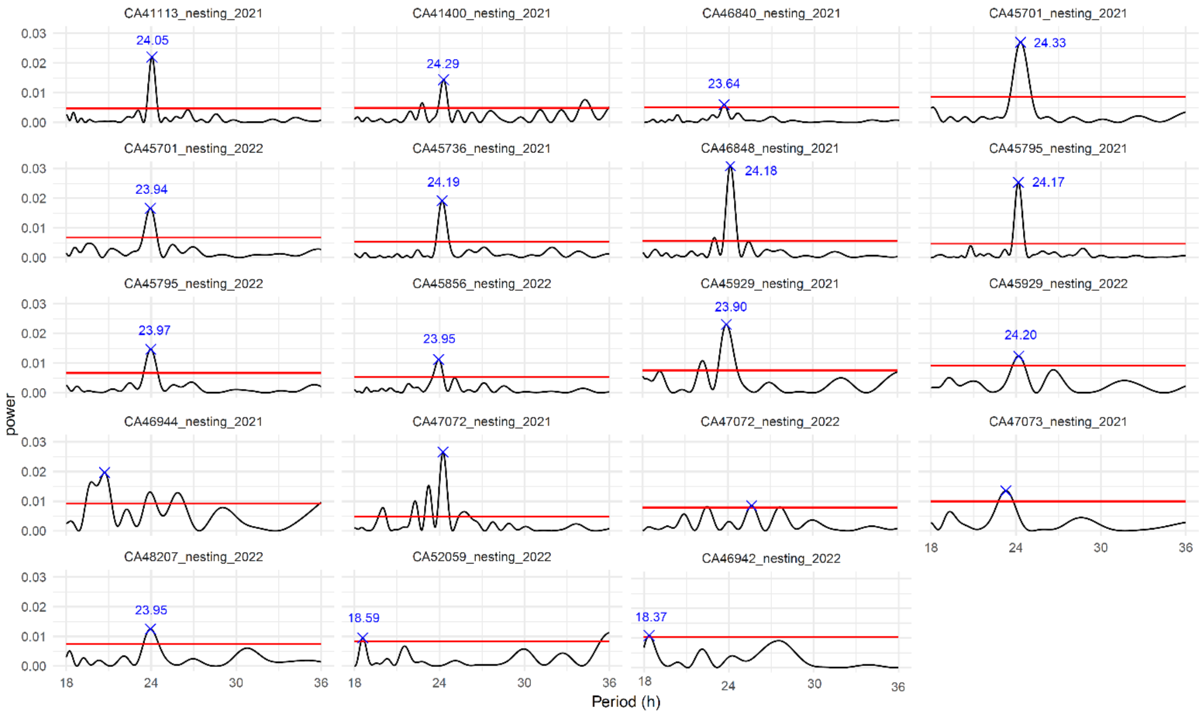

Figure 44: Lomb-Scargle periodograms showing ***diel peak period in activity during the nesting phase*** *for individual geese. The analysis identified periodicity between 18 and 36 hours. The headers indicate goose ID, year and breeding stage.*

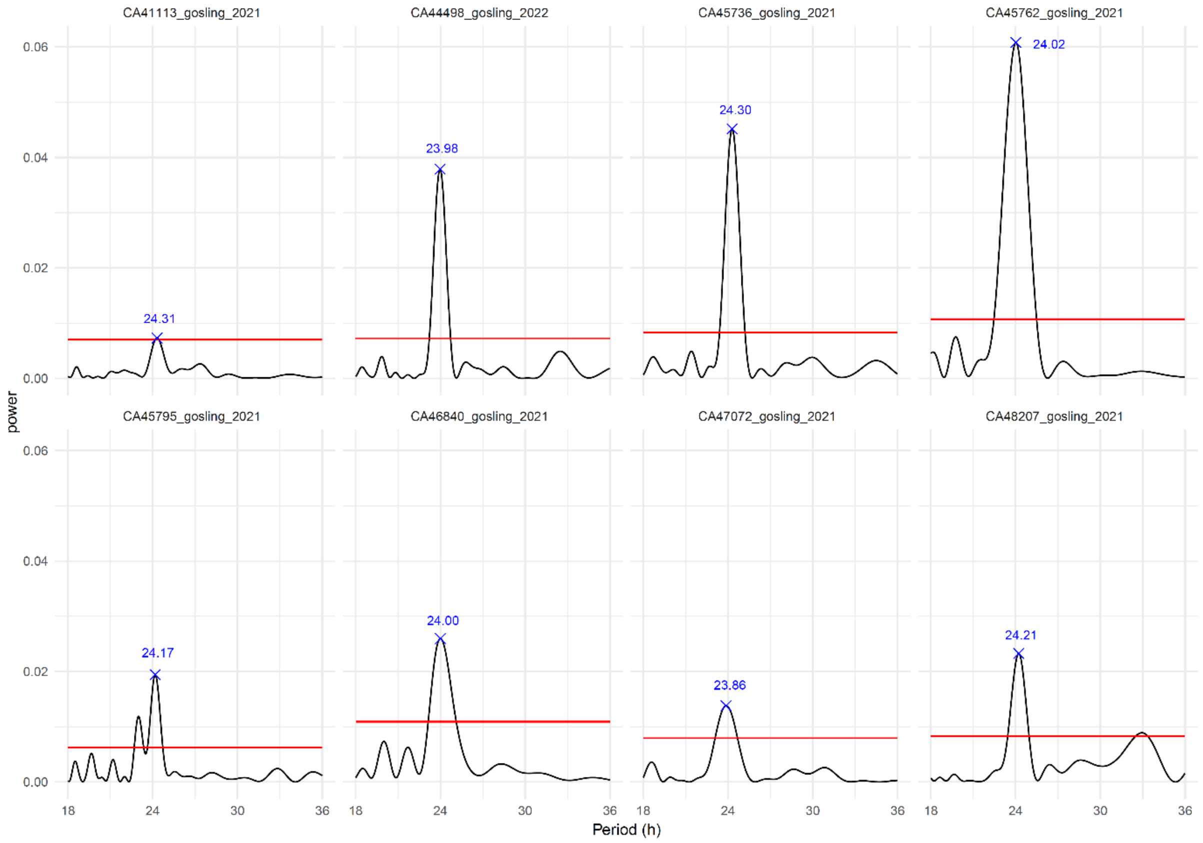

*Figure 45: Lomb-Scargle periodograms showing **diel peak period in activity during the gosling phase** for individual geese. The analysis identified periodicity between 18 and 36 hours. The headers indicate goose ID, year and breeding stage.*

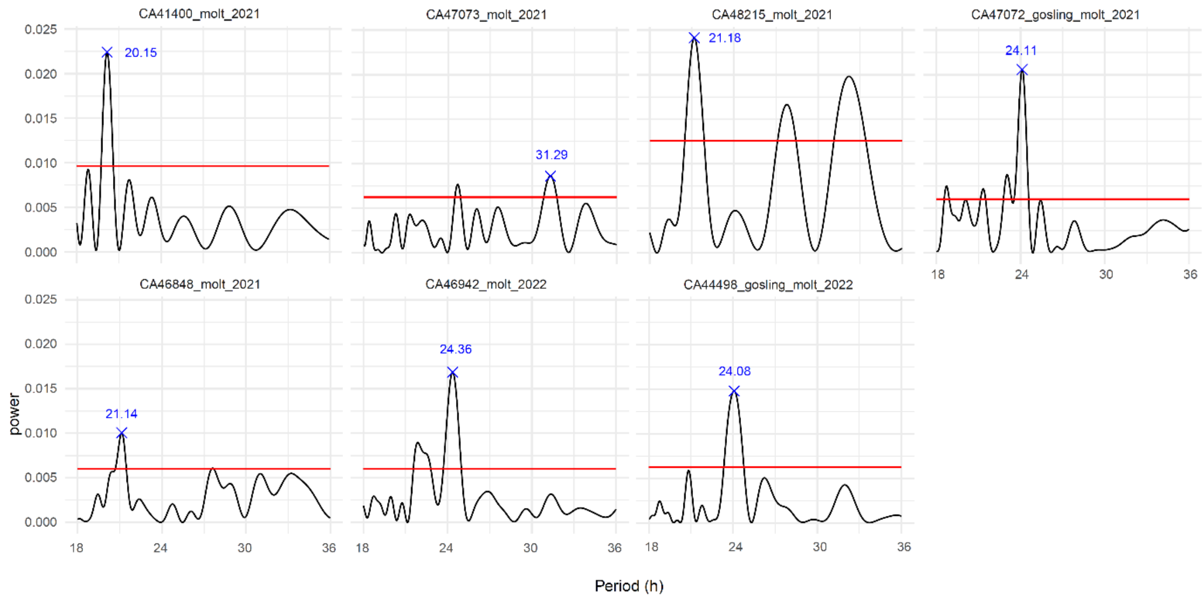

*Figure 46: Lomb-Scargle periodograms showing **diel peak period in activity during the molt phase** for individual geese. We pooled two goslings & molt phases with the molt phase data to increase sample size. The analysis identified periodicity between 18 and 36 hours. The headers indicate goose ID, year and breeding stage.*

Tides in Ny-Ålesund

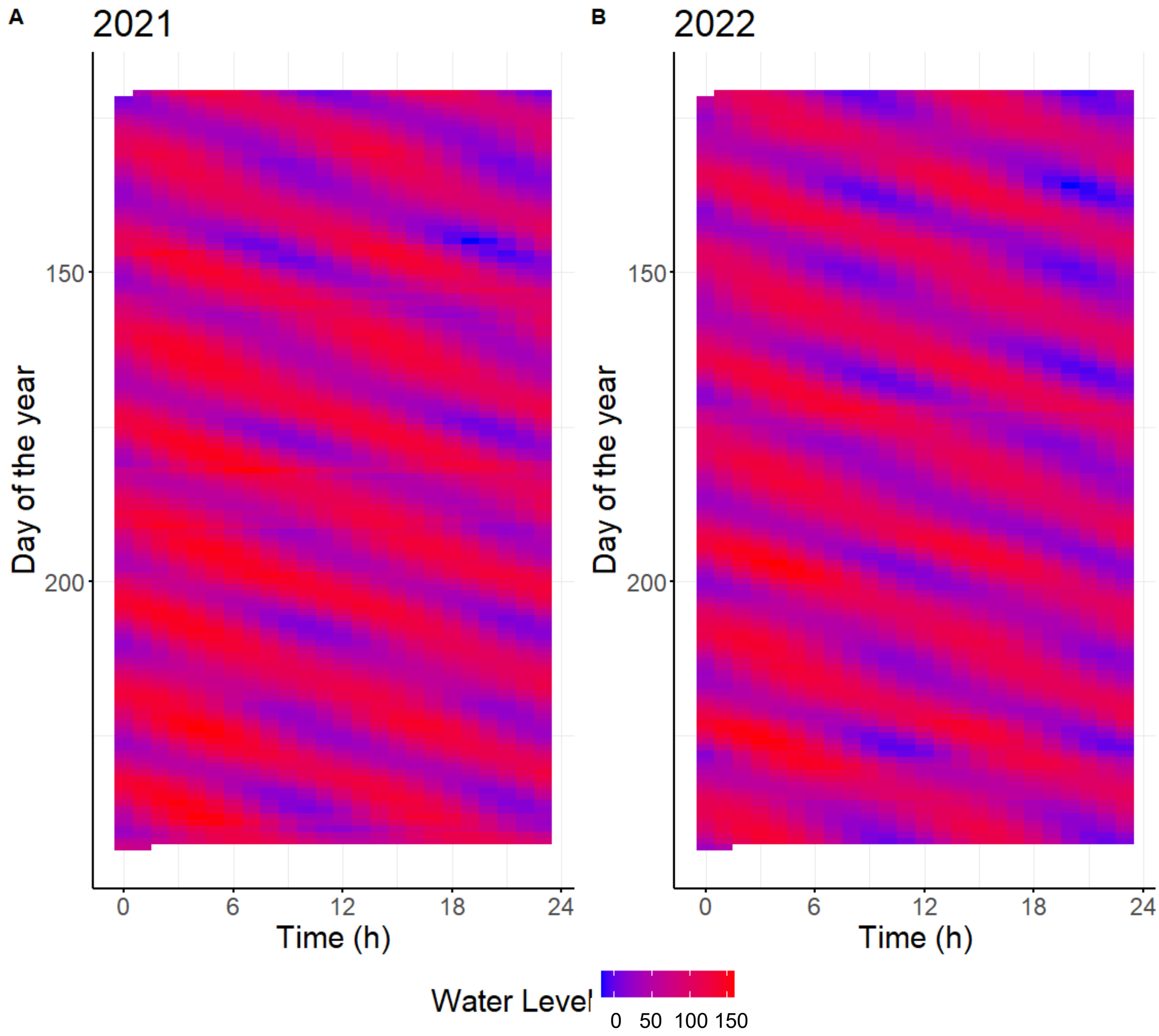

Figure 47: Heatmap plot of water level observations collected every 10 minutes over 24 hours by Statens kartverk sjø (Norwegian Hydrographic Service, www.kartverket.no) in Ny-Ålesund. The graphs show the two study years, A) 2021 and B) 2022, from the beginning of May (1st of May is Julian date 121 of the year) until the end of August (31st of August is Julian date 243 day of the year). Ny-Ålesund has a semi-daily tide with a period of ~12.4 hours.

Post hoc analysis on locations of geese during the gosling and molt stages

The graphs below (Figure 48) show the locations of geese during the gosling and molt phases. Colors indicate if coordinates were either outside, i.e. in the intertidal area/at sea (blue), or inside the 25-meter buffer, which was placed on land (pink). The graph title refers to the individual metal ring ID of each goose, the year and the breeding stage. Kartdata © Norsk Polarinstitutt.

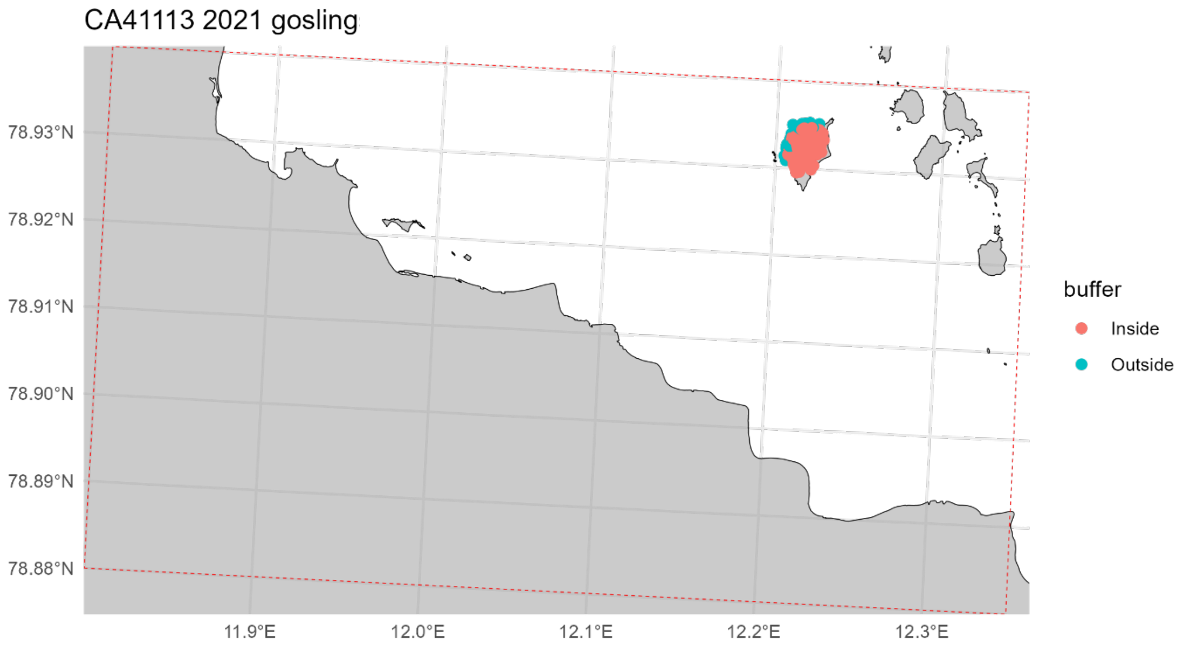

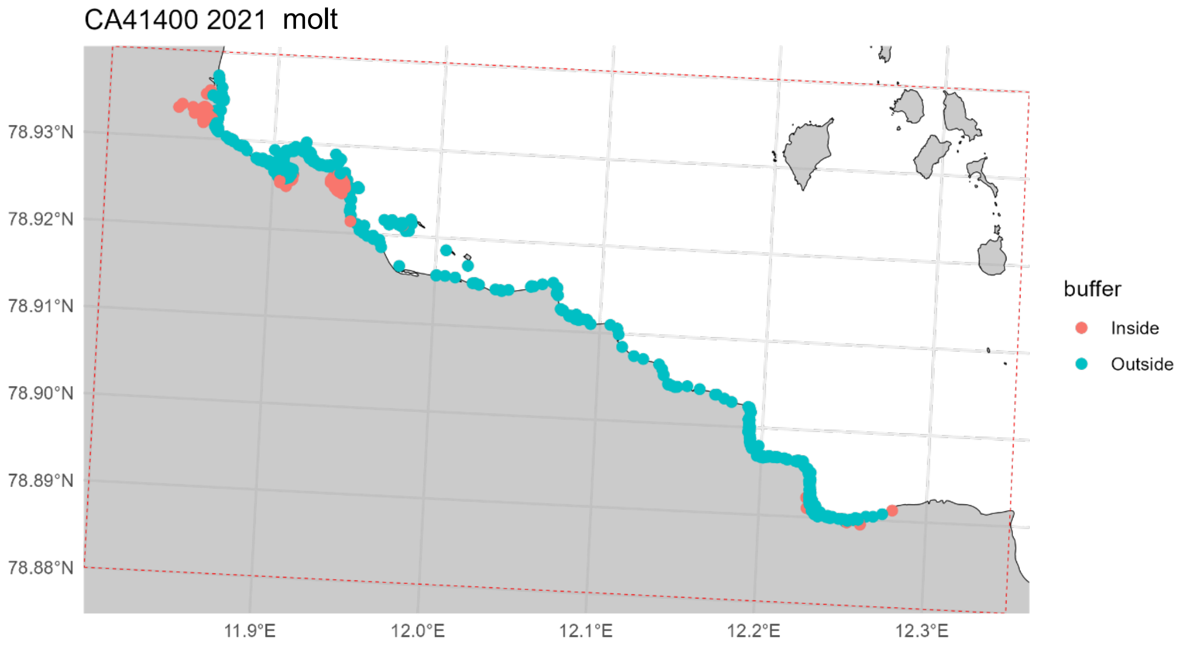
CA41400 2021 molt
78.93°N
78.92°N
78.91°N
78.90°N
78.89°N
78.88°N
11.9°E
12.0°E
12.1°E
12.2°E
12.3°E
buffer
Inside
Outside

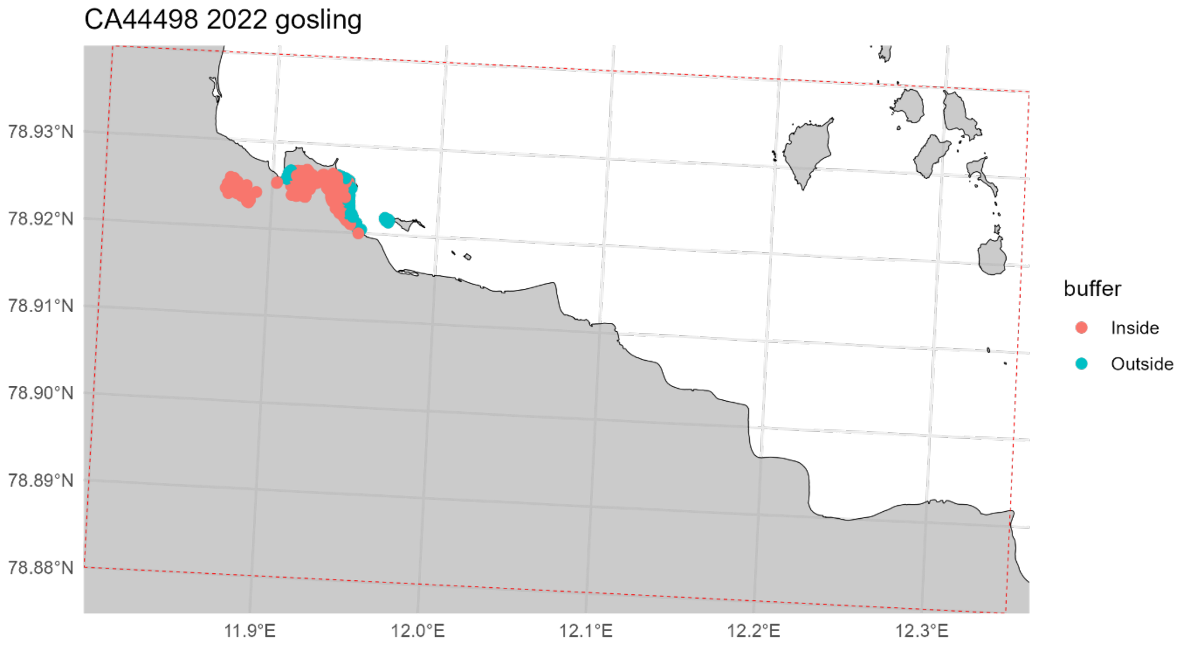
CA44498 2022 gosling
78.93°N
78.92°N
78.91°N
78.90°N
78.89°N
78.88°N
11.9°E
12.0°E
12.1°E
12.2°E
12.3°E
buffer
Inside
Outside

CA44498 2022 gosling & molt

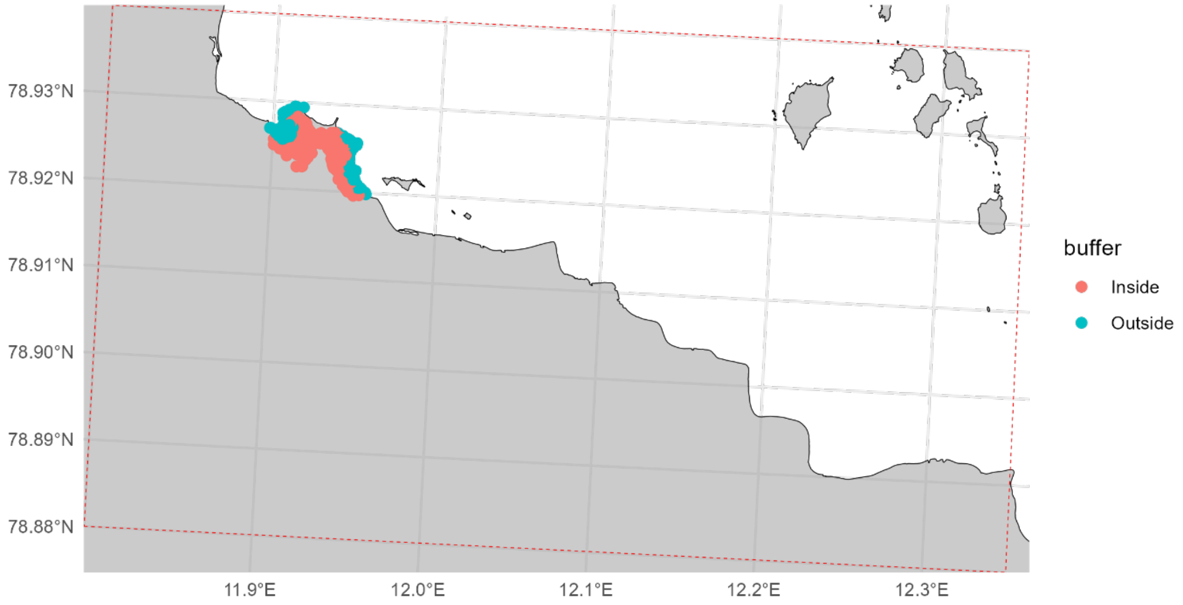

CA45736 2021 gosling

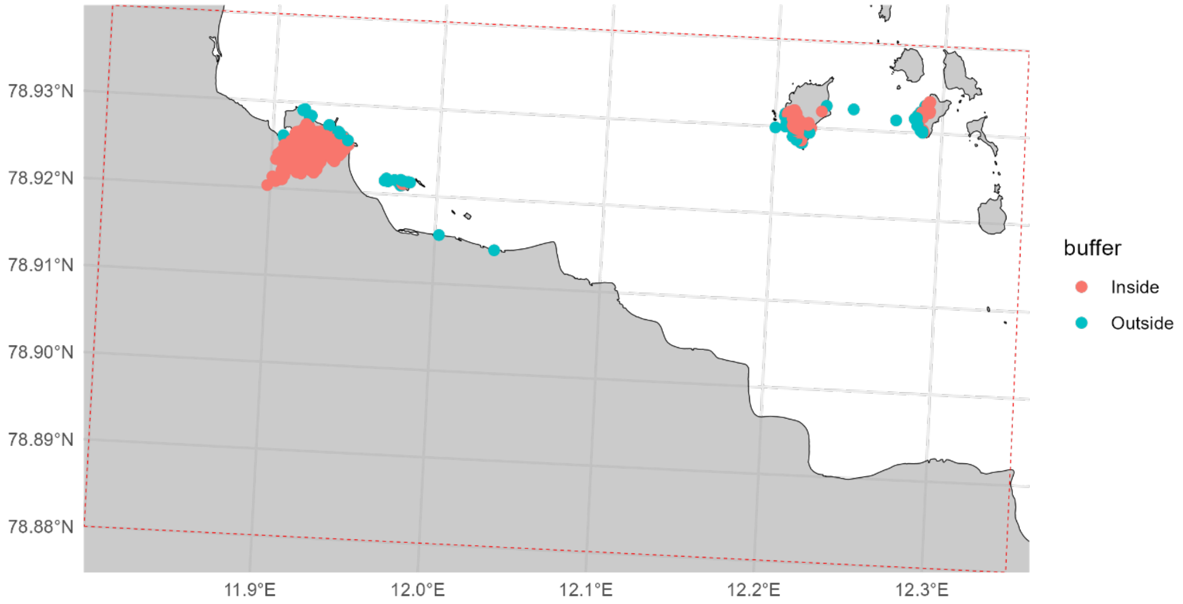

CA45762 2021 gosling

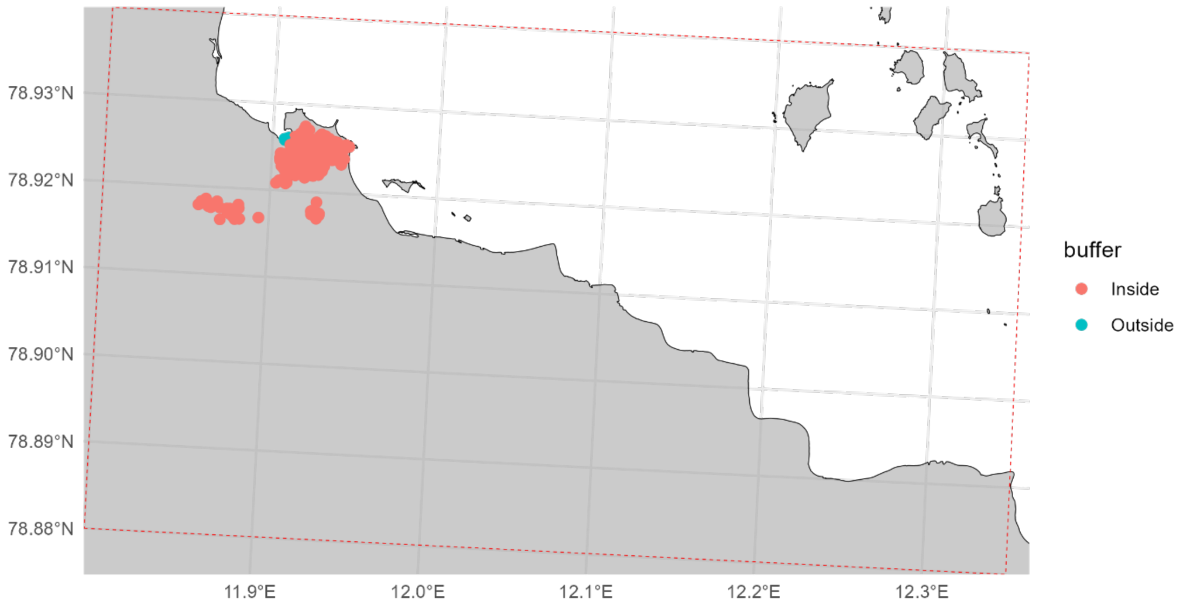

CA45762 2021 molt

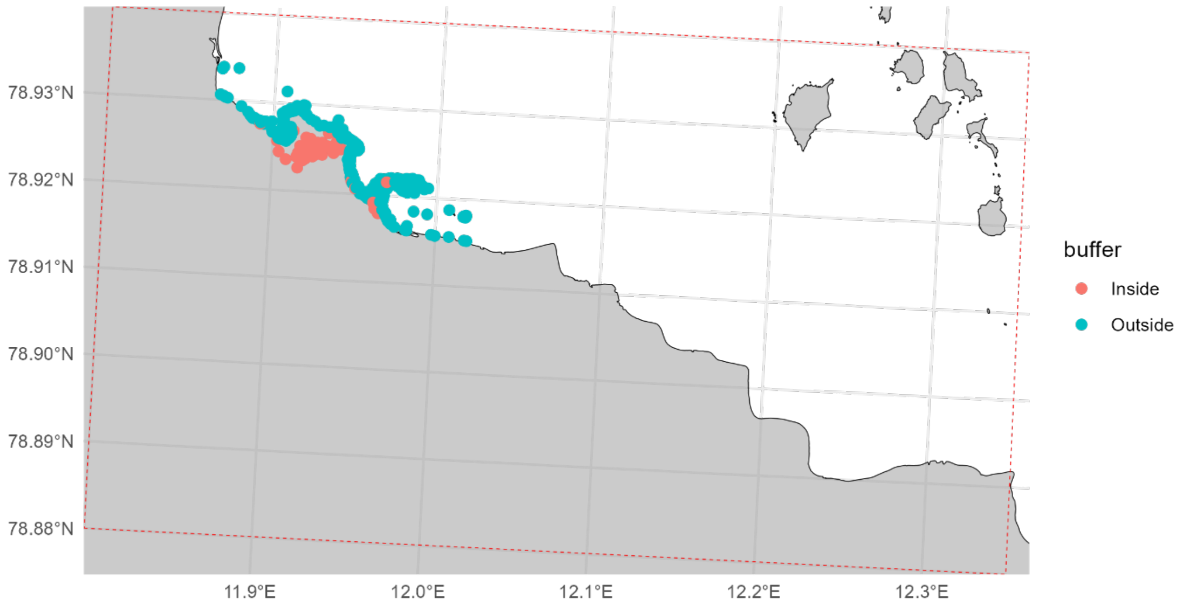

CA45795 2021 gosling

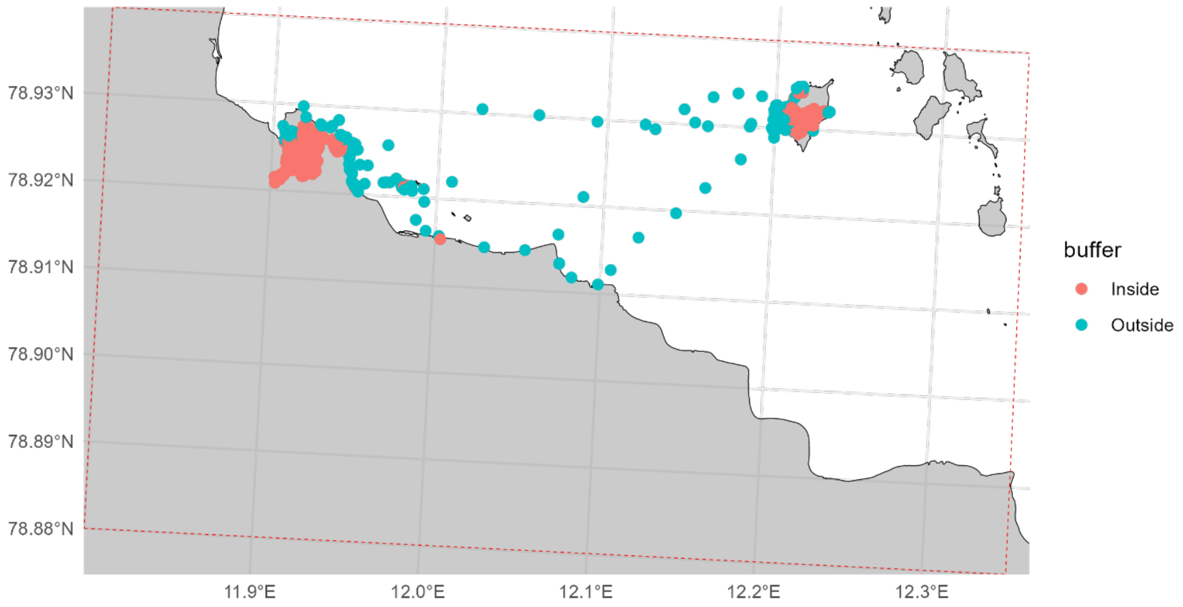

CA45795 2021 molt

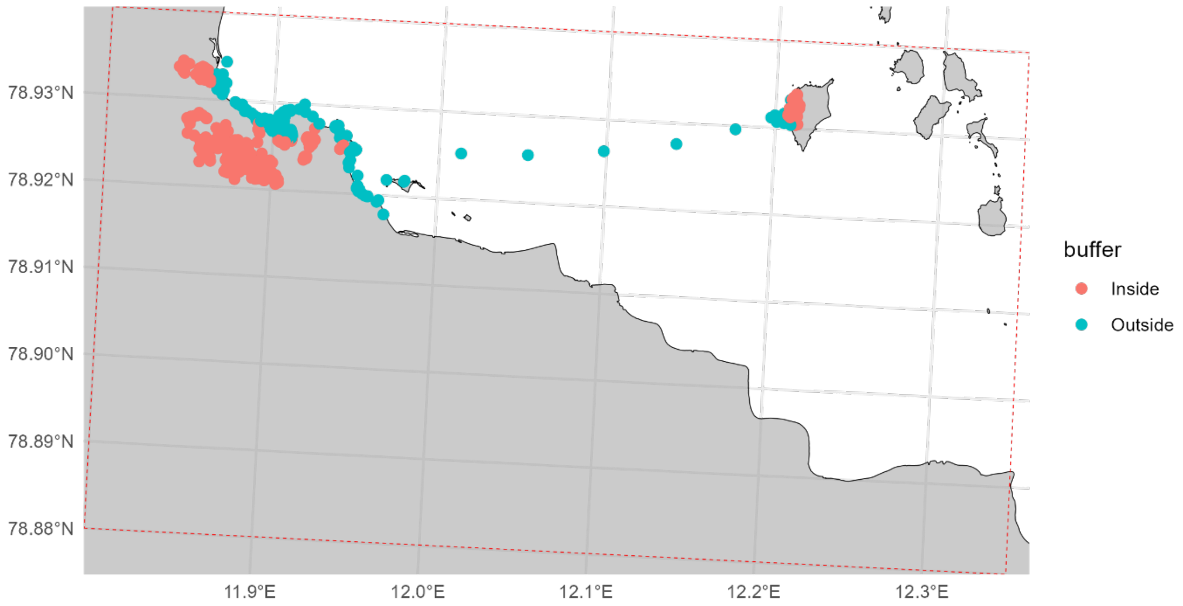

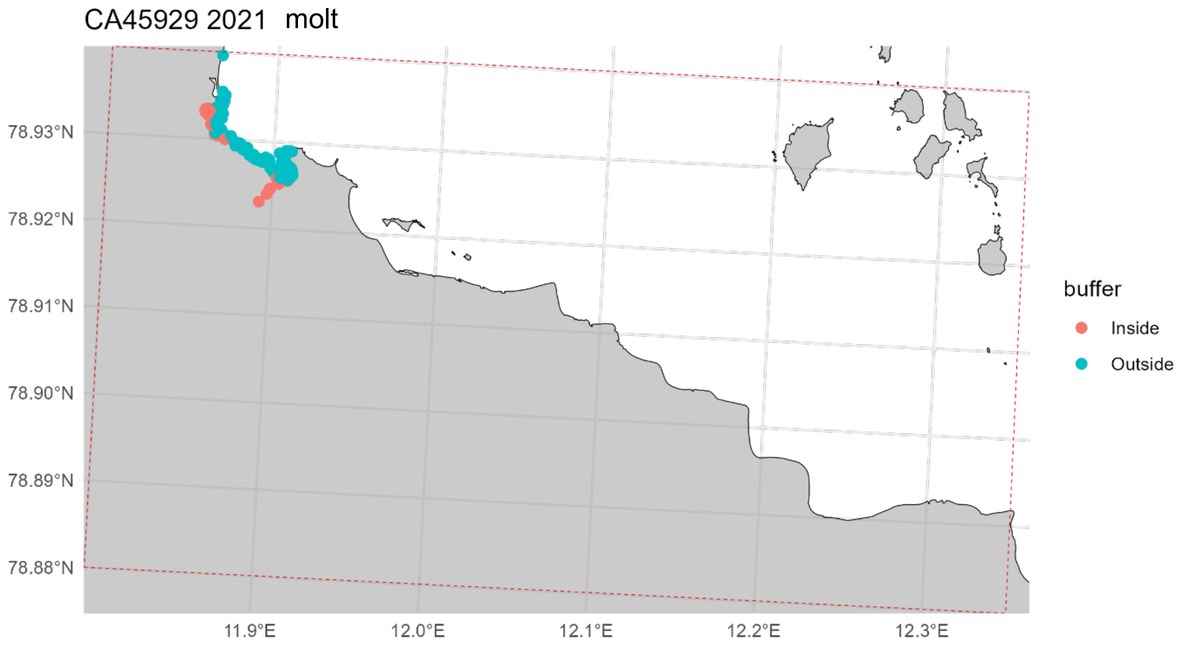
CA45929 2021 molt
78.93°N
78.92°N
78.91°N
78.90°N
78.89°N
78.88°N
11.9°E
12.0°E
12.1°E
12.2°E
12.3°E
buffer
Inside
Outside

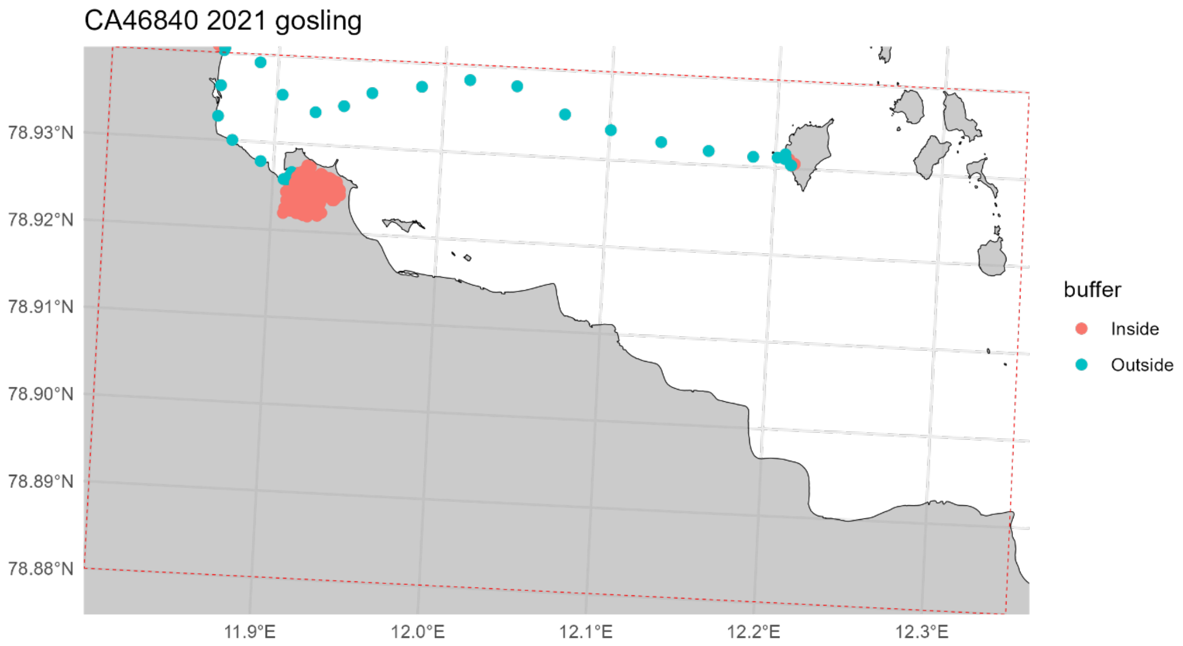
CA46840 2021 gosling
78.93°N
78.92°N
78.91°N
78.90°N
78.89°N
78.88°N
11.9°E
12.0°E
12.1°E
12.2°E
12.3°E
buffer
Inside
Outside

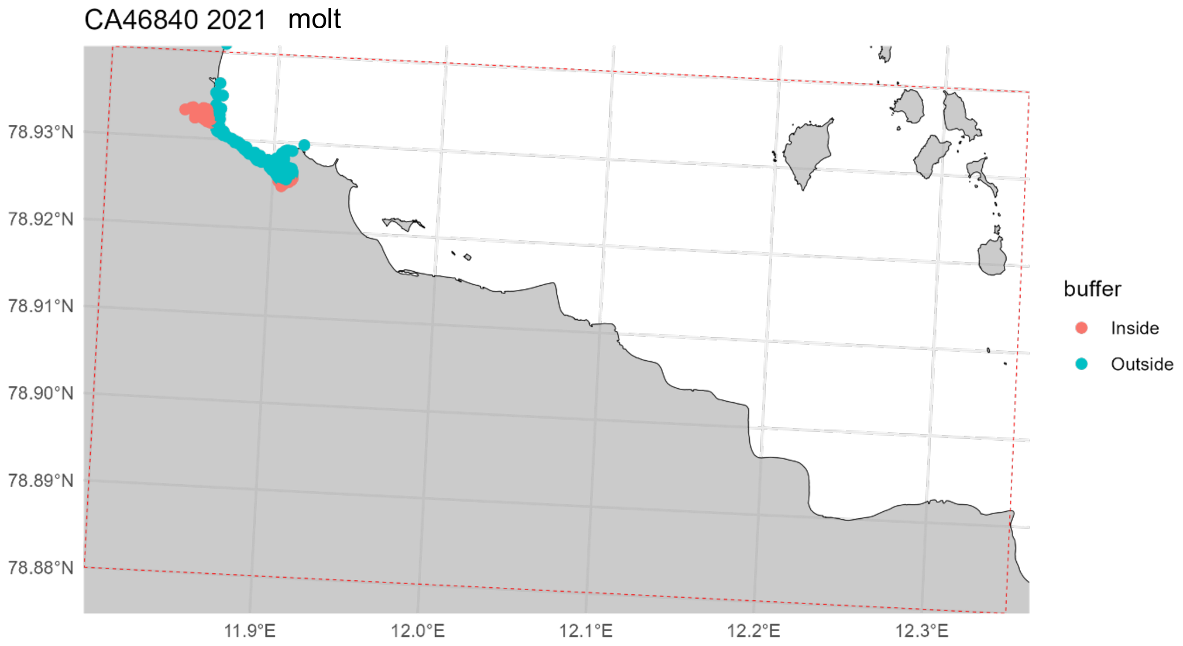
CA46840 2021 molt
78.93°N
78.92°N
78.91°N
78.90°N
78.89°N
78.88°N
11.9°E
12.0°E
12.1°E
12.2°E
12.3°E
buffer
Inside
Outside

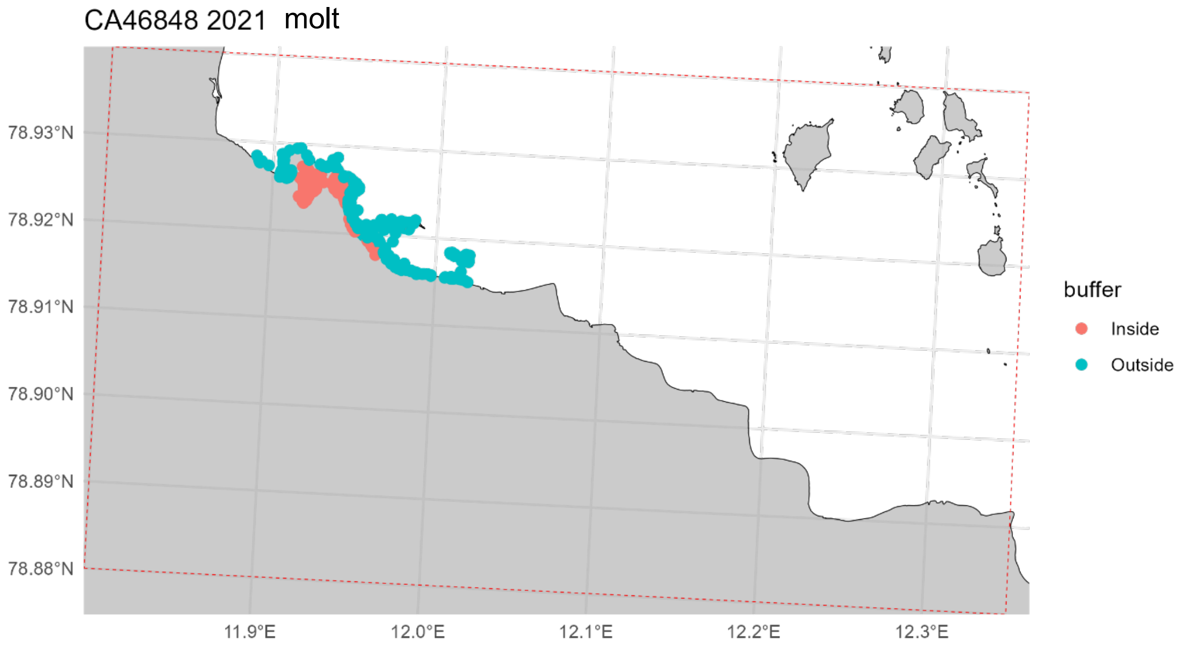
CA46848 2021 molt
78.93°N
78.92°N
78.91°N
78.90°N
78.89°N
78.88°N
11.9°E
12.0°E
12.1°E
12.2°E
12.3°E
buffer
Inside
Outside

CA46942 2022 molt

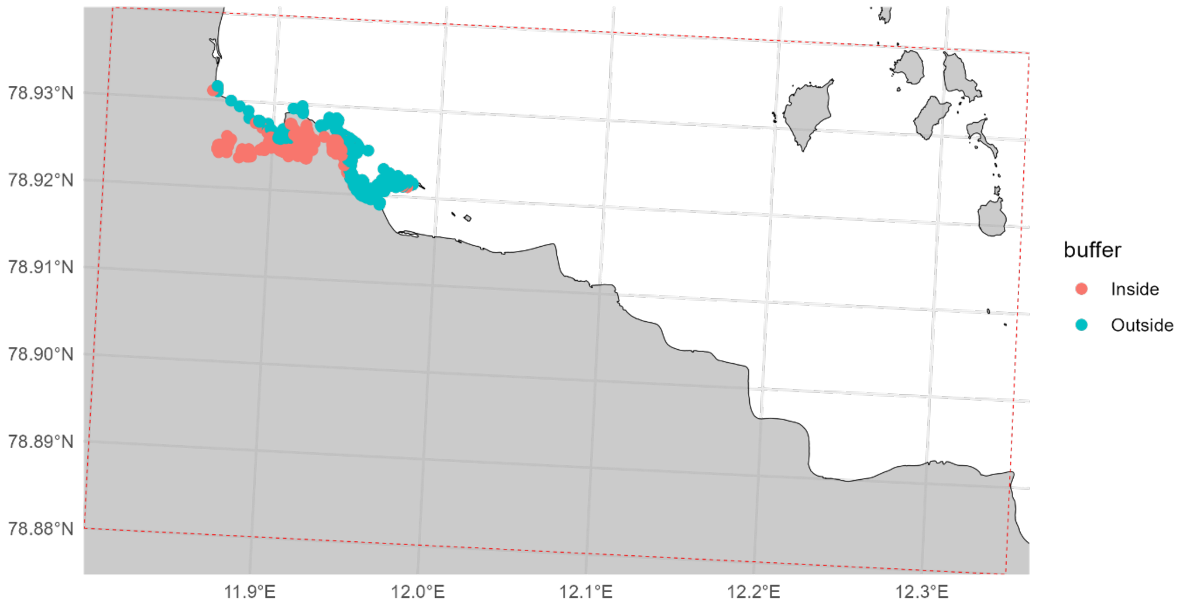

CA46944 2021 molt

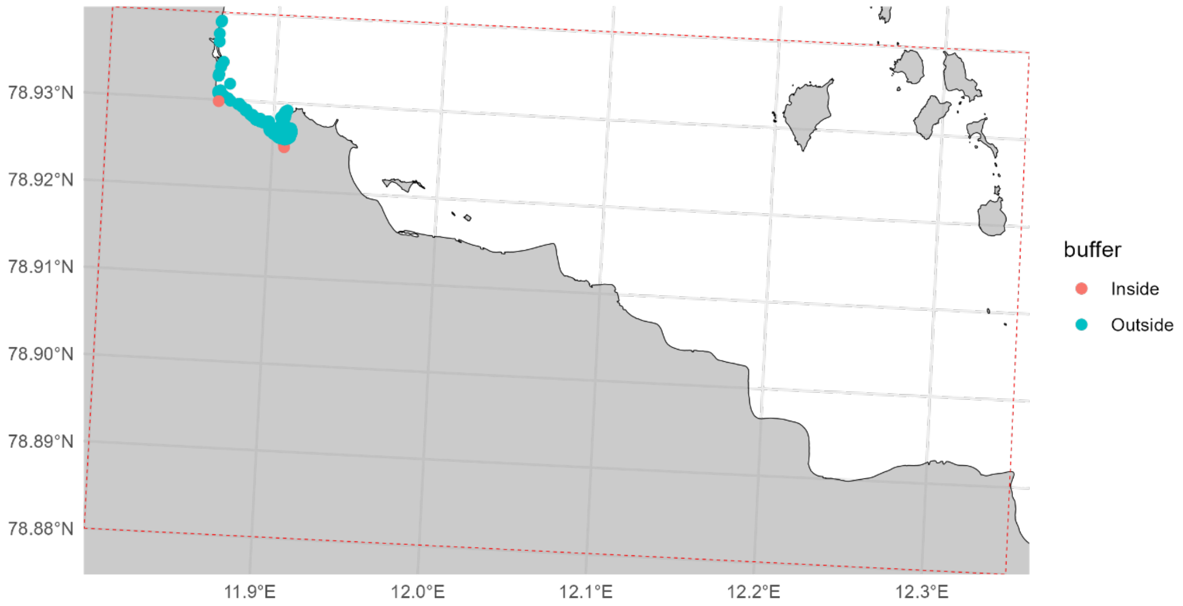

CA47072 2021 gosling

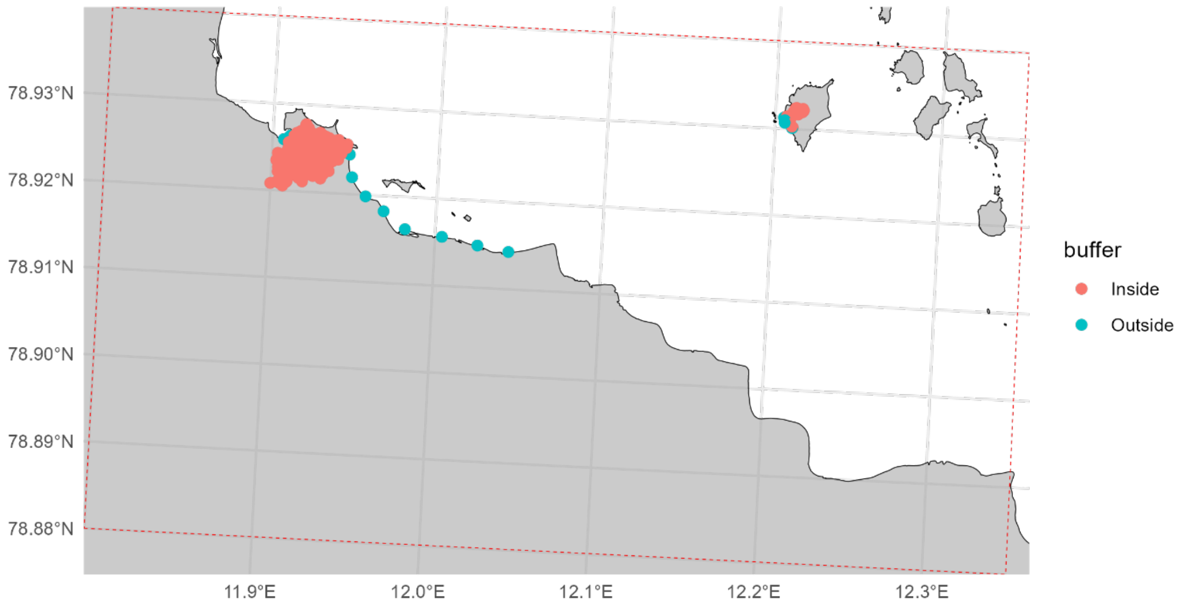

CA47072 2021 gosling & molt

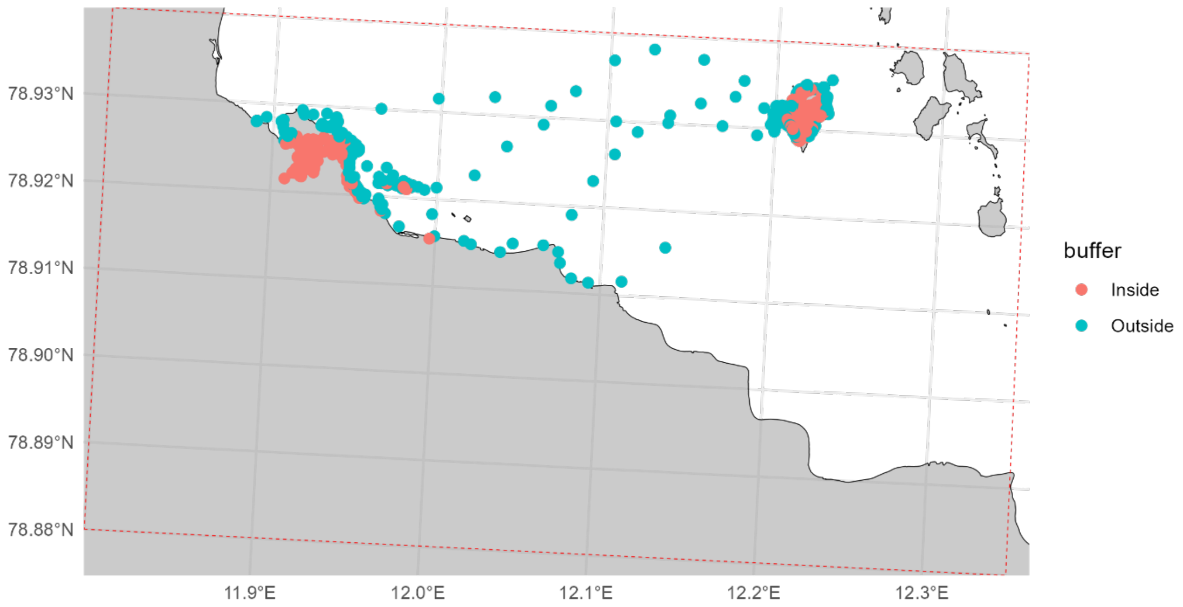

CA47073 2021 molt

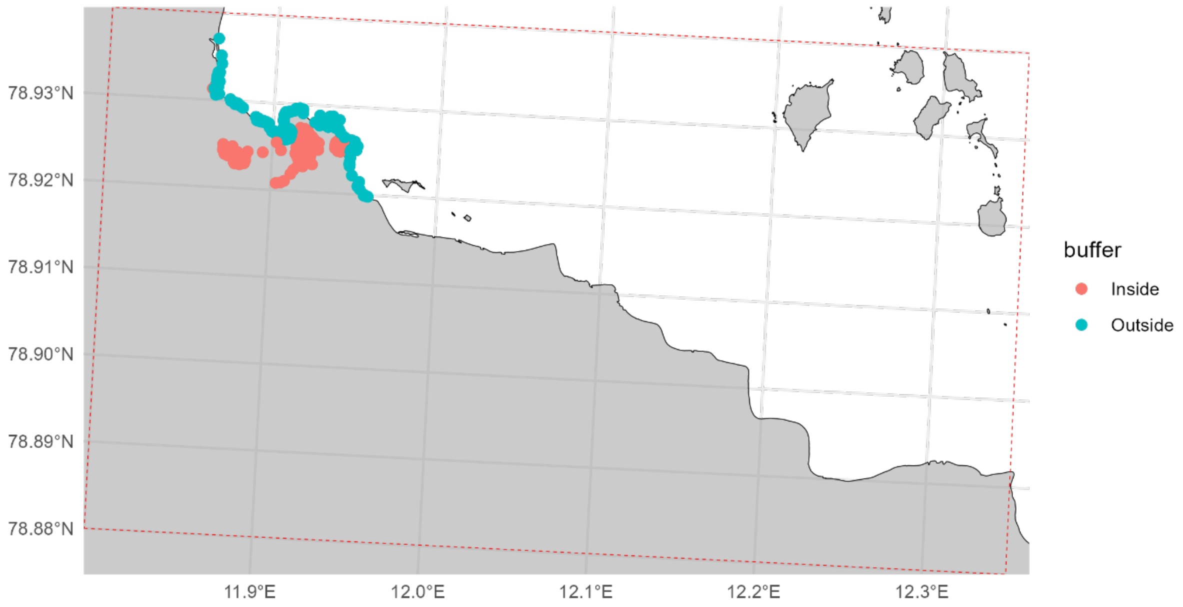

CA48207 2021 gosling

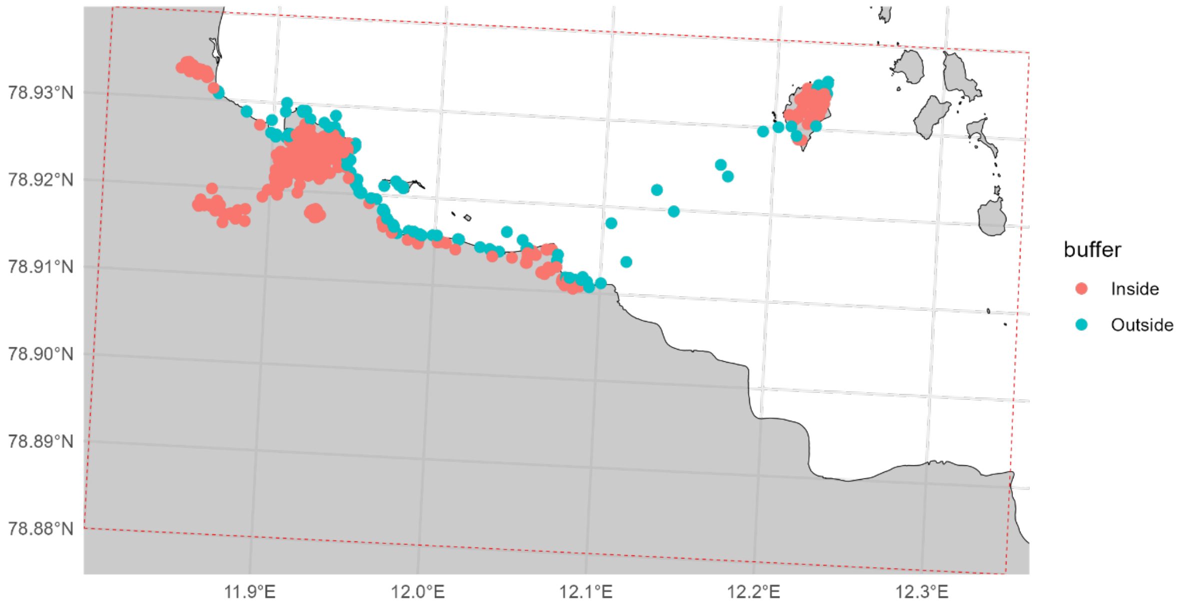

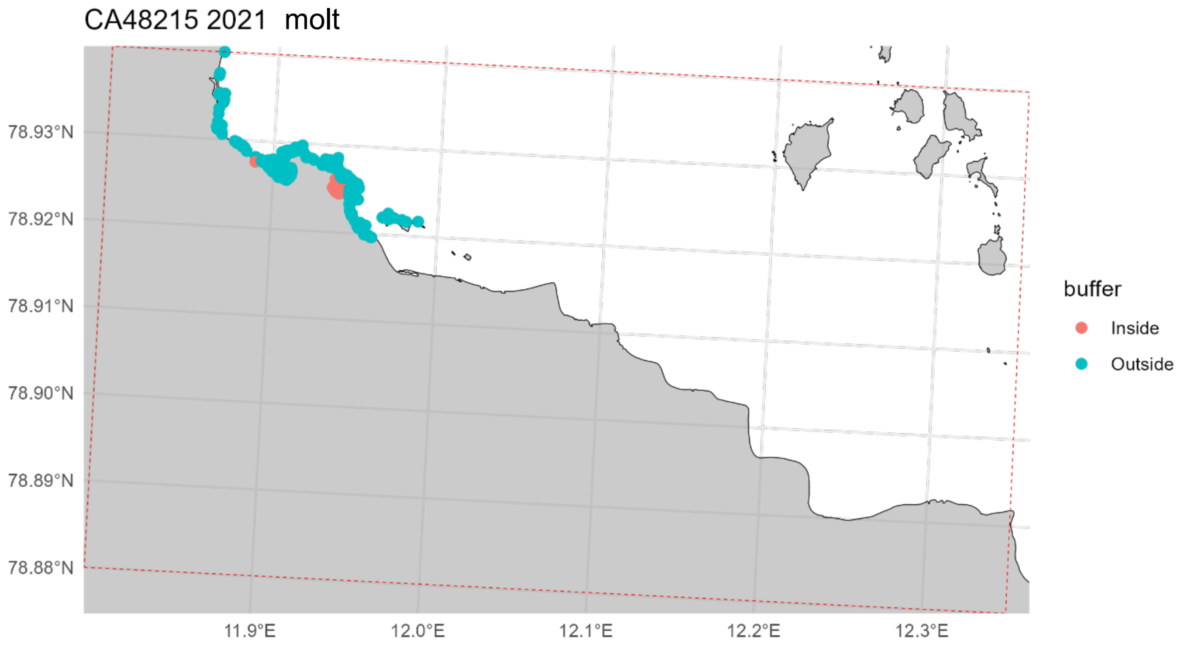
CA48215 2021 molt
78.93°N
78.92°N
78.91°N
78.90°N
78.89°N
78.88°N
11.9°E
12.0°E
12.1°E
12.2°E
12.3°E
buffer
Inside
Outside
